# Lymphoid gene expression supports neuroprotective microglia function

**DOI:** 10.1038/s41586-025-09662-z

**Published:** 2025-11-05

**Authors:** Pinar Ayata, Jessica M. Crowley, Matthew F. Challman, Vinaya Sahasrabuddhe, Maud Gratuze, Sebastian Werneburg, Diogo Ribeiro, Emma C. Hays, Violeta Durán-Laforet, Travis E. Faust, Philip Hwang, Francisco Mendes Lopes, Chrysa Nikopoulou, Sarah Buchholz, Robert E. Murphy, Taoyu Mei, Anna A. Pimenova, Carmen Romero-Molina, Francesca Garretti, Tulsi A. Patel, Claudia De Sanctis, Angie V. Ramirez Jimenez, Megan Crow, Felix D. Weiss, Jason D. Ulrich, Edoardo Marcora, John W. Murray, Felix Meissner, Andreas Beyer, Dan Hasson, John F. Crary, Dorothy P. Schafer, David M. Holtzman, Alison M. Goate, Alexander Tarakhovsky, Anne Schaefer

**Affiliations:** 1 Nash Family Department of Neuroscience, Department of Psychiatry, Friedman Brain Institute, Icahn School of Medicine at Mount Sinai, New York, NY USA; 2 Ronald M. Loeb Center for Alzheimer’s Disease, Center for Glial Biology, Icahn School of Medicine at Mount Sinai, New York, NY USA; 3 Neuroscience Initiative, Advanced Science Research Center, Graduate Program in Biology, Graduate Program in Biochemistry, Graduate Program in Neuroscience, The City University of New York Graduate Center, New York, NY USA; 4 Department of Neurology, Hope Center for Neurological Disorders, Charles F. and Joanne Knight Alzheimer’s Disease Research Center, Washington University School of Medicine, St. Louis, MO USA; 5 Institute of Neurophysiopathology (INP), University of Aix-Marseille, CNRS UMR 7051, Marseille, France; 6 Department of Neurobiology, Brudnick Neuropsychiatric Research Institute, University of Massachusetts Chan Medical School, Worcester, MA USA; 7 Department of Ophthalmology and Visual Sciences, Kellogg Eye Center, Michigan Neuroscience Institute, Department of Molecular & Integrative Physiology, University of Michigan, Michigan Medicine, Ann Arbor, MI USA; 8 Max Planck Institute for Biology of Ageing, Cologne, Germany; 9 Cologne Excellence Cluster for Aging and Aging-Associated Diseases (CECAD), University of Cologne, Cologne, Germany; 10 Center for Molecular Medicine Cologne, Faculty of Medicine and University Hospital Cologne, Institute for Genetics, Faculty of Mathematics and Natural Sciences, University of Cologne, Cologne, Germany; 11 Department of Genetics & Genomic Sciences, Icahn School of Medicine at Mount Sinai, New York, NY USA; 12 Department of Pathology, Department of Artificial Intelligence & Human Health, Neuropathology Brain Bank and Research CoRE, Icahn School of Medicine at Mount Sinai, New York, NY USA; 13 Bioinformatics for Next-Generation Sequencing (BiNGS) Core, Tisch Cancer Institute, Department of Oncological Sciences, Icahn School of Medicine at Mount Sinai, New York, NY USA; 14 Department of Human Genetics, Genentech Inc., South San Francisco, CA USA; 15 Institute of Innate Immunity, Department for Systems Immunology and Proteomics, Medical Faculty, University of Bonn, Bonn, Germany; 16 German Rheumatology Research Center (DRFZ), A Leibniz Institute, Berlin, Germany; 17 Columbia Center for Human Development, Center for Stem Cell Therapies, Department of Medicine, Columbia University Vagelos College of Physicians and Surgeons, New York, NY USA; 18 Laboratory of Immune Cell Epigenetics and Signaling, The Rockefeller University, New York, NY USA

**Keywords:** Neuroimmunology, Alzheimer's disease, Microglia, Epigenetics in the nervous system, Molecular neuroscience

## Abstract

Microglia, the innate immune cells of the brain, play a defining role in the progression of Alzheimer’s disease (AD)^1^. The microglial response to amyloid plaques in AD can range from neuroprotective to neurotoxic^2^. Here we show that the protective function of microglia is governed by the transcription factor PU.1, which becomes downregulated following microglial contact with plaques. Lowering PU.1 expression in microglia reduces the severity of amyloid disease pathology in mice and is linked to the expression of immunoregulatory lymphoid receptor proteins, particularly CD28, a surface receptor that is critical for T cell activation^3,4^. Microglia-specific deficiency in CD28, which is expressed by a small subset of plaque-associated PU.1^low^ microglia, promotes a broad inflammatory microglial state that is associated with increased amyloid plaque load. Our findings indicate that PU.1^low^ CD28-expressing microglia may operate as suppressive microglia that mitigate the progression of AD by reducing the severity of neuroinflammation. This role of CD28 and potentially other lymphoid co-stimulatory and co-inhibitory receptor proteins in governing microglial responses in AD points to possible immunotherapy approaches for treating the disease by promoting protective microglial functions.

## Abstract (web summary)

PU.1^low^ CD28-expressing microglia may act as suppressive cells in Alzheimer’s disease, mitigating its progression by reducing neuroinflammation and amyloid plaque load, indicating potential immunotherapeutic approaches for treatment.

## Main

Alzheimer’s disease is associated with diverse phenotypic changes in microglia^5,6^, resulting in distinct microglial states, including protective (aimed at amyloid clearance and neuroprotection^7–9^) and harmful (associated with microglia-driven neuroinflammation and toxicity)^10,11^. These opposing states are accompanied by broad changes in gene expression^12–17^, suggesting that defined transcriptional programs in microglia may govern their neuroprotective or neurotoxic functions. Here we present evidence that the neuroprotective state of microglia is governed by reduced expression of PU.1, a non-canonical pioneer transcription factor that functions as a master regulator of myeloid and lymphoid lineage differentiation^18–20^. PU.1 regulates lineage-specific gene expression in a dose-dependent manner^21–23^. More recently, PU.1 abundance in microglia has been linked to AD risk. Human genetic data show that a common variant, located within the 3′ untranslated region of the PU.1-encoding gene *SPI1* and associated with reduced PU.1 expression in myeloid cells, correlates with delayed disease onset and reduced severity^24^.

**Fig. 1:**
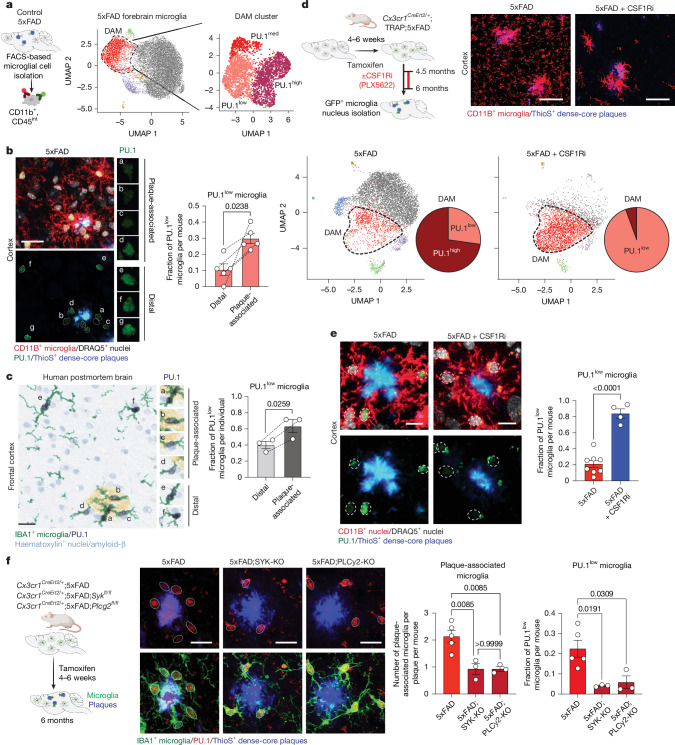
Plaque-associated microglia include PU.1^low^ subpopulation. **a**, Uniform manifold approximation and projections (UMAPs) of ex vivo isolated forebrain microglia from 8-month-old wild-type and 5xFAD mice (two males per group) show distinct PU.1 expression states (PU.1^low^, PU.1^med^ and PU.1^high^) among DAM^12^ (red). **b**,**c**, PU.1 protein is reduced in a subset of plaque-associated microglia in the cortex of 6-month-old 5xFAD mice (*n* = 5; one female and four males) (**b**) and in the frontal cortex of individuals with AD (79–91 years; *n* = 3; one female and two males) (**c**). Bar graphs show the proportion of PU.1^low^ cells in plaque-associated microglia versus distal microglia, analysed using a paired two-tailed *t*-test. **d**,**e**, Plaque-associated PU.1^low^ microglia are resistant to CSF1R inhibition (CSF1Ri). **d**, UMAP visualizations of lineage-traced cortical microglia (TRAP^28^) from 6-month-old control (one female) or CSF1Ri-fed 5xFAD mice (two males), analysed using single-nucleus sequencing (DAM outlined with dotted line). The pie chart shows PU.1^low^ fraction within DAM. **e**, Reduced PU.1 protein expression in CSF1Ri-resistant cortical microglia in the cortices of 6-month-old CSF1Ri (*n* = 4; one female and three males) versus control diet-fed (*n* = 8 mice; six females and two males) 5xFAD mice. Bar graph shows the proportion of PU.1^low^ microglia, analysed using an unpaired two-tailed *t*-test. **f**, Microglial SYK/PLCγ2 signalling promotes the plaque-associated PU.1^low^ state. Representative images and quantification of PU.1 expression in cortical microglia from 6-month-old 5xFAD;*Cx3cr1*^*CreErt2/+*^ (*n* = 5; three females and two males), 5xFAD–SYK-KO (5xFAD;*Cx3cr1*^*CreErt2/+*^*;Syk*^*fl/fl*^; *n* = 3; one female and two males) and 5xFAD–PLCγ2-KO mice (5xFAD;*Cx3cr1*^*CreErt2/+*^*;Plcg2*^*fl/fl*^; *n* = 3; two females and one male). Bar graphs show plaque-normalized microglial numbers and PU.1^low^ microglia fractions, as determined by ordinary one-way ANOVA with multiple comparisons. Microglial nuclei identified using Imaris (**b**,**f**) and CellProfiler (**e**) in dotted circles. Bar graphs show the mean ± s.e.m. with individual points. Scale bars, 20 μm (**b**,**c**,**f**), 40 μm (**d**), 10 μm (**e**). Illustrations in **a**, **d** and **f** were created using BioRender (https://biorender.com). Source data

**Extended Data Fig. 1:**
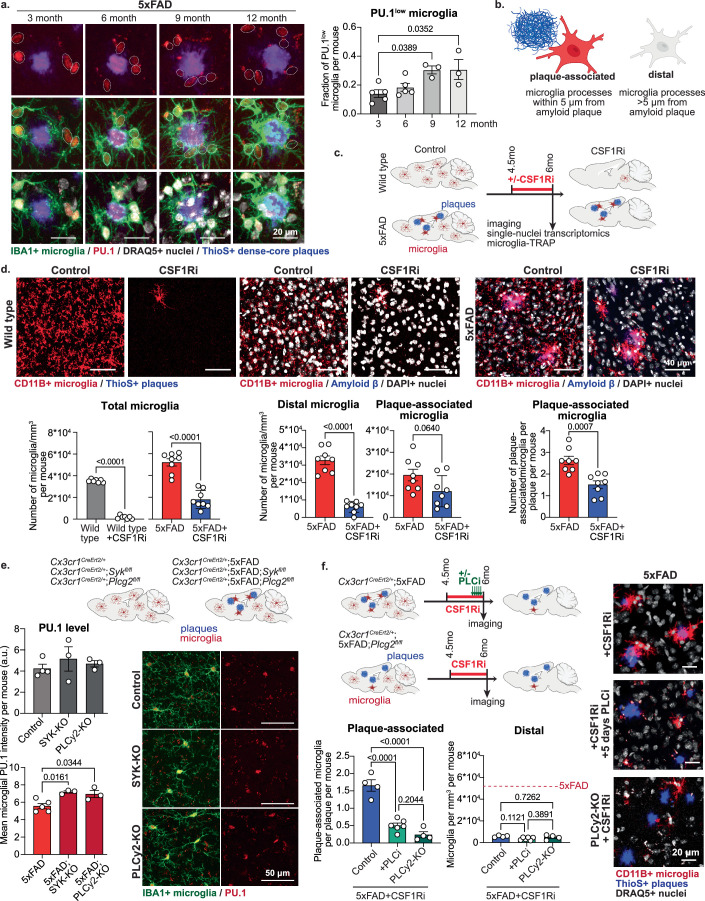
Downregulation of PU.1 is plaque signal-driven and associated with CSF1R-independent microglia survival (related to Fig. 1). (**a**) Increased PU.1^low^ microglia abundance during AD disease progression. PU.1 protein expression in cortical microglia is shown. Scale bar: 20 μm. Microglial nuclei, as identified by Imaris, are indicated in dotted circles. The bar graphs show the size of PU.1^low^ microglia subpopulation in the cortex of 3-month-old (n = 5 mice, 2♀, 3♂), 6-month-old (n = 5 mice, 3♀, 2♂), 9-month-old (n = 3 mice, 3♀), and 12-month-old (n = 3 mice, 1♀, 2♂) 5xFAD mice—ordinary one-way ANOVA with multiple comparisons. (**b**) Spatial definition of plaque-associated microglia (closest microglia processes within <5 μm of ThioS+ plaque surface) versus distal microglia. (**c**) Strategy for microglia depletion using CSF1R inhibitor (CSF1Ri, PLX5622, 1200 ppm diet) for ~6 weeks starting at 4.5 months of age in control and 5xFAD mice. (**d**) CSF1R-independent microglia survival at the plaque. Representative immunofluorescence images are shown. CSF1Ri treatment ablates >80% of distal microglia while >60% of plaque-associated microglia remain. Bar graphs show: Left: the total number of all microglia per mm^3^ in the cortex of 6-month-old control diet- and CSF1Ri diet-fed wild-type (left) or 5xFAD mice (right). Middle: the number of distal (left) and plaque-associated (right) microglia per mm^3^. Right: the average number of plaque-associated microglia normalized to plaque numbers in control or CSF1Ri-treated 5xFAD mice. Wild-type mice: n = 7/group, 4♀, 3♂. 5xFAD mice: n = 8 mice/group, 5♀, 3♂—unpaired two-tailed t-test. (**e**) SYK/PLCγ2 are required for PU.1^low^ microglia at the plaque. Representative immunofluorescence images are shown. Bar graphs show the average PU.1 protein intensity in microglia in the cortex of 6-month-old control (*Cx3cr1*^*CreErt2/+*^, n = 4 mice, 2♀, 2♂), SYK-KO (*Cx3cr1*^*CreErt2/+*^*; Syk*^*fl/fl*^, n = 3 mice, 2♀, 1♂), and PLCγ2-KO mice (*Cx3cr1*^*CreErt2/+*^*; Plcg2*^*fl/fl*^, n = 3 mice, 3♂), as well as in 5xFAD (5xFAD; *Cx3cr1*^*CreErt2/+*^, n = 5 mice, 3♀, 2♂), 5xFAD-SYK-KO (5xFAD; *Cx3cr1*^*CreErt2/+*^*; Syk*^*fl/fl*^, n = 3 mice, 1♀, 2♂), and 5xFAD; PLCγ2-KO mice (5xFAD; *Cx3cr1*^*CreErt2/+*^*; Plcg2*^*fl/fl*^, n = 3 mice, 2♀, 1♂)—ordinary one-way ANOVA with multiple comparisons. (**f**) CSF1R-independent survival of plaque-associated PU.1^low^ microglia depends on PLCγ2 activity. Bar graph shows the number of plaque-associated microglia per plaque and number of distal microglia per mm^3^ in CSF1Ri-treated 5xFAD (n = 4 mice, 2♀, 2♂), CSF1Ri- and PLCi-treated 5xFAD (pharmacological PLC inhibition: PLC inhibitor, 8.7 mg/kg U73122, n = 6 mice, 2♀, 4♂) and CSF1Ri-treated 5xFAD; PLCγ2-KO mice (genetic microglia-specific PLCγ2 deletion: n = 4 mice, 3♀, 1♂)—ordinary one-way ANOVA with multiple comparisons. The dotted line indicates the expected number of distal microglia in the cortex of 5xFAD mice (without CSF1Ri treatment). Bar graphs with individual data points show mean ± SEM. Illustrations in **b**, **c**, **e** and **f** were created using BioRender (https://biorender.com). Source data

We found that PU.1 expression in microglia is regulated not only genetically but also epigenetically by the microenvironment. Using the amyloid-based 5xFAD mouse model of the disease^25^, we identified a distinct subpopulation of microglia, among the previously described disease-associated microglia (DAM)^12^ or neurodegenerative microglia^13^, that displays lower PU.1/*Spi1* expression compared with the rest of the population (Fig. 1a, Supplementary Fig. 1a–h and Supplementary Table 1). These PU.1^low^ microglia preferentially co-localized with amyloid plaques, in both 5xFAD mice (Fig. 1b and Supplementary Fig. 1i) and individuals with AD (Fig. 1c and Supplementary Table 2), and increase in numbers with disease progression (Extended Data Fig. 1a). Unlike non-plaque-associated (distal microglia; Extended Data Fig. 1b) or PU.1^high^ plaque-associated microglia, this subpopulation appears independent of the activity of the essential microglial survival receptor CSF1R^26,27^ (Fig. 1d,e and Extended Data Fig. 1b–d). The CSF1R-independent survival of PU.1^low^ microglia at plaques may reflect either their non-microglial origin or the engagement of alternative compensatory survival pathways. Lineage-tracing experiments using a microglia-specific genetic approach (translating ribosome affinity purification (TRAP) mice^28^; Fig. 1d, Supplementary Fig. 2 and Supplementary Table 3) confirmed that the plaque-associated CSF1R-independent PU.1^low^ cells, despite showing low *Csf1r* gene expression (Supplementary Figs. 1h and 2f), are bona fide microglia.

The PU.1^low^ microglial population at amyloid plaques may be established either through a pre-existing PU.1^low^ microglial subpopulation that is recruited specifically to plaques or by the downregulation of PU.1 expression in microglia in response to plaque-associated cues. Our data support the latter mechanism. Microglial accumulation at plaques and plaque-driven morphological and transcriptional responses depend on microglial surface receptor-mediated signalling^8,12,13,29^, which involves activation of the tyrosine kinase (SYK)^9,30^ and one of its key downstream targets (PLCγ2)^31,32^. Microglia-specific inactivation of either SYK (5xFAD;*Cx3cr1*^*CreERT2/+*^*;Syk*^*fl/fl*^; referred to as 5xFAD;SYK-KO) or PLCγ2 (5xFAD;*Cx3cr1*^*CreERT2/+*^*;Plcg2*^*fl/fl*^; referred to as 5xFAD;PLCγ2-KO), which impairs microglial engagement with the plaques^9,30,32^ (Fig. 1f), also significantly reduces the number of PU.1^low^ microglia (Fig. 1f and Extended Data Fig. 1e) and averts their CSF1R-independent survival at the plaques (Extended Data Fig. 1f).

**Extended Data Fig. 2:**
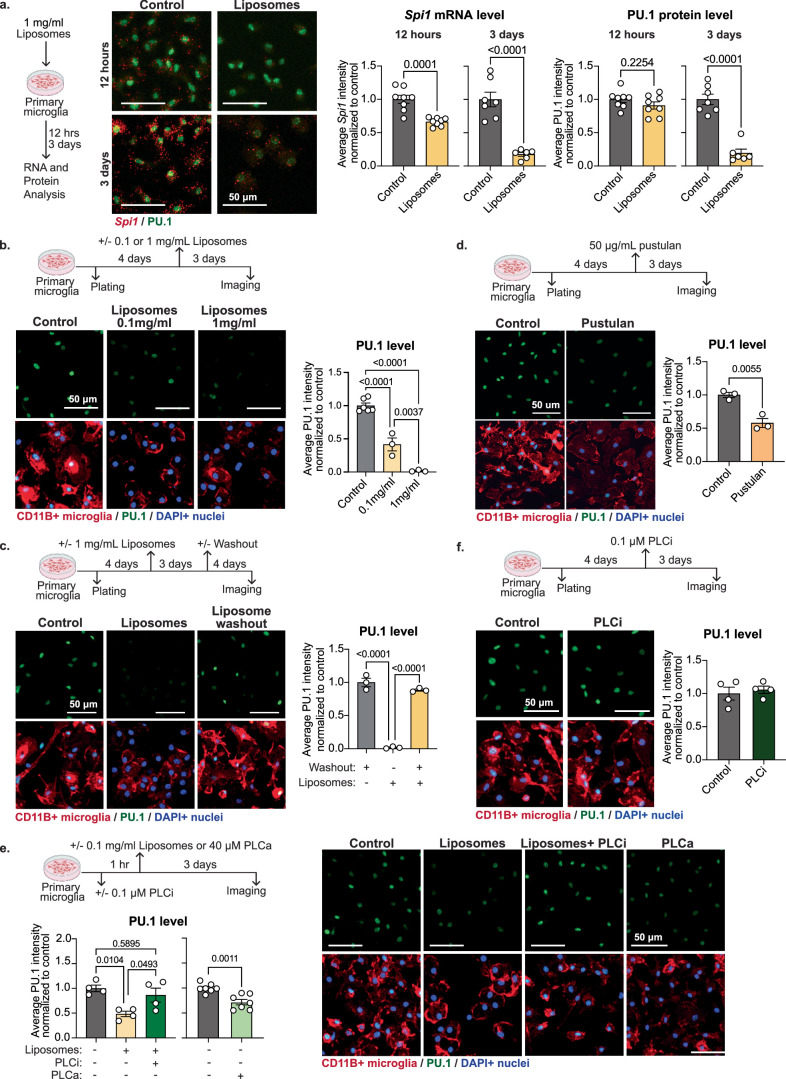
Signal-induced downregulation of PU.1 in microglia (related to Fig. 1). (**a**) Experimental design (left), representative images (middle), and quantification (right) of *Spi1* mRNA (measured by single-molecule in situ hybridization) and PU.1 protein expression (measured by immunofluorescence) in cultured primary microglia treated with vehicle or 1 mg/ml of phosphatidylserine/phosphatidylcholine (PS/PC) liposomes for either 12 h or 3 days. *Spi1* mRNA: red, PU.1 protein: green. Scale bar: 50 μm. The bar graphs show *Spi1* mRNA expression (left) and PU.1 protein expression (right) normalized to the mean of the control for each time point—an unpaired two-tailed t-test was used. Quantification shown is from 2 independent experiments (12 h: experiment 1: n = 4 wells/group, experiment 2: n = 4 wells/group; total: n = 8 wells/group. 3 days: experiment 1: n = 4 wells/group, experiment 2: n = 3 wells/control, n = 2 wells/liposome-treated; total: n = 7 wells/control group, n = 6 wells/liposome-treated group). (**b**) Experimental design (top left), representative immunofluorescence images (bottom left), and quantification (right) of PU.1 intensity in cultured primary microglia treated with vehicle or PS/PC liposomes at 0.1 or 1 mg/ml for 3 days. Scale bar: 50 μm. The bar graph shows quantification of PU.1 protein expression in microglia nuclei with treatment of vehicle control (n = 6 wells), 0.1 mg/ml PS/PC liposomes (n = 3 wells), and 1 mg/ml PS/PC (n = 3 wells), normalized to the mean of vehicle control—ordinary one-way ANOVA with multiple comparisons. The experiment was independently repeated. (**c**) Liposome-induced PU.1 downregulation is reversible. Experimental design (top left), representative immunofluorescence images (bottom left), and quantification (right) of PU.1 levels in cultured primary microglia treated with vehicle, PS/PC liposomes at 1 mg/ml for 3 days, and received washout (4 days). CD11B+ microglia: red, PU.1: green, DAPI+ nuclei: blue. Scale bar: 50 μm. The bar graph shows PU.1 levels in microglial nuclei in vehicle-treated, 3-day liposome-treated (1 mg/ml), or liposome-treated followed by washout (n = 3 wells/group)—ordinary one-way ANOVA with multiple comparisons. (**d**) CLEC7A agonist-induced PU.1 downregulation. Experimental design (top), representative immunofluorescence images (bottom left), and quantification (bottom right) of PU.1 expression in cultured primary microglia treated with vehicle or CLEC7A agonist, 50 μg/mL pustulan for 3 days—unpaired two-tailed t-test (n = 3 wells/group). (**e**) Experimental design (top left), representative immunofluorescence images of PU.1 expression (right), and quantification (bottom left) of cultured primary microglia treated with vehicle or 0.1 mg/ml liposomes for 3 days following a 1-h pre-treatment of PLC inhibitor (PLCi, 0.1 μM U73122) or vehicle DMSO, or only PLC activator (PLCa, 40 μM *m*-3M3FBS). CD11B+ microglia: red, PU.1: green, DAPI+ nuclei: blue. Scale bar: 50 μm. The bar graphs show quantification of PU.1 expression in microglia nuclei normalized to vehicle control. Left for PLCi: n = 4 wells/group—ordinary one-way ANOVA with multiple comparisons. The PLCi experiment was independently repeated. The PLCa experiment: Data are pooled from two independent experiments (experiment 1: n = 3/group; experiment 2: n = 4/group)—unpaired two-tailed t-test. (**f**) PLC inhibitor treatment alone does not change baseline PU.1 expression. Experimental design (top), representative immunofluorescence images (bottom left), and quantification (bottom right) of PU.1 expression in cultured primary microglia treated with vehicle (n = 4 wells) or PLC inhibitor (PLCi, 0.1 μM U73122) for 3 days—an unpaired two-tailed t-test was used (n = 4 wells/group). Bar graphs with individual data points show mean ± SEM. Illustrations in **a**–**f** were created using BioRender (https://biorender.com). Source data

Activation of SYK–PLCγ2-dependent signalling pathways in plaque-associated microglia occurs following the engagement of surface receptor proteins, including TREM2, CLEC7A and FcγR^33^. To confirm that PU.1 downregulation is driven by surface receptor-mediated signalling, we exposed ex vivo isolated microglia to phosphatidylserine/phosphatidylcholine (PS/PC)-containing liposomes^31^ or β-1,6-glucan pustulan^34^, which engage TREM2 and CLEC7A, respectively. Both ligands induced PU.1 downregulation (Extended Data Fig. 2a–d), which was attenuated by pharmacologic inhibition of downstream phospholipase C (PLC) activity^35^ (Extended Data Fig. 2e,f). Conversely, activation of PLC^36^ was sufficient to downregulate PU.1 even in the absence of external ligands (Extended Data Fig. 2e), confirming that activation of the SYK–PLCγ2 signalling pathways triggered by plaque-associated cues can regulate PU.1 expression in microglia.

**Fig. 2:**
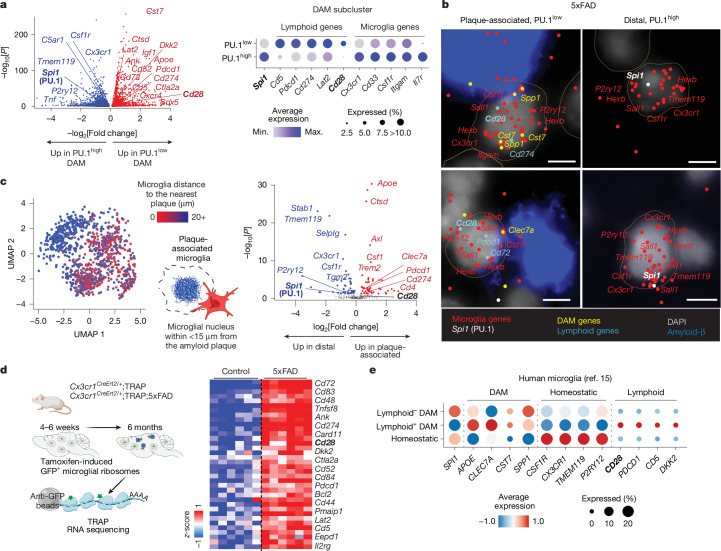
Upregulation of lymphoid genes in PU.1^low^ plaque-associated microglia. **a**, The volcano plot shows genes upregulated in PU.1^low^ (red) versus PU.1^high^ (blue) DAM in 8-month-old wild-type and 5xFAD mice (two males per group), as determined by Wilcoxon rank-sum test. The dot plot highlights selected myeloid/microglia-associated and lymphoid-lineage-associated genes in PU.1^low^ and PU.1^high^ DAM. **b**,**c**, PU.1^low^ lymphoid gene-expressing cortical microglia localize near amyloid plaques. Independently repeated twice. **b**, Representative MERFISH images of plaque-associated and distal microglia from an 8-month-old male 5xFAD brain. **c**, UMAP of spatial microglia distribution to the nearest plaque using MERFISH. The volcano plot shows differentially expressed genes in plaque-associated microglia (red; less than 15 μm to plaques) versus distal microglia (blue; greater than 15 μm to plaques), as determined by Wilcoxon rank-sum test. **d**, Lymphoid protein-encoding RNAs associate with ribosomes in lineage-traced microglia. The heat map (*z*-score) shows selected lymphoid lineage genes identified using microglia-specific TRAP sequencing^28^ from the cortices of 6-month-old 5xFAD mice (*Cx3cr1*^*CreErt2/+*^*;*5xFAD;*Eef1a1*^*LSL.eGFPL10a/+*^) versus control (*Cx3cr1*^*CreErt2/+*^*;Eef1a1*^*LSL.eGFPL10a/+*^ mice; *n* = 6 per group; three females and three males), as determined using DESeq2. Each column represents an individual mouse. **e**, PU.1^low^ lymphoid gene-expressing microglia are present in individuals with AD. The dot plots show scaled average expression of selected genes in human microglia^15^ organized into homeostatic, lymphoid^+^ DAM and lymphoid^–^ DAM clusters. Scale bar, 5 μm. Illustrations in **c** and **d** were created using BioRender (https://biorender.com). Max., maximum; Min., minimum.

**Extended Data Fig. 3:**
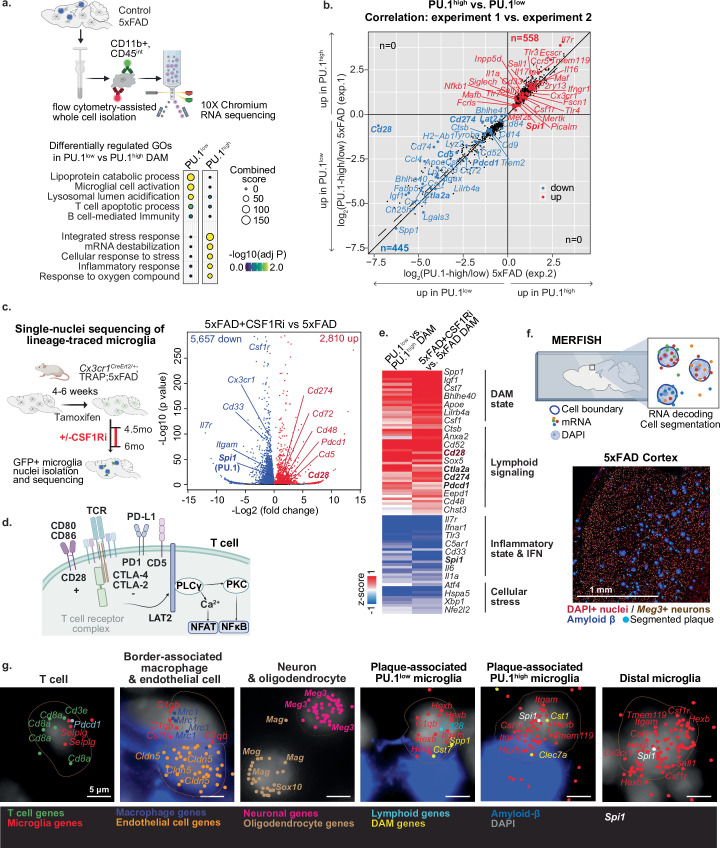
PU.1^low^ plaque-associated microglia express lymphoid genes (related to Fig. 2). (**a, b**) PU.1^low^ plaque-associated microglia show lymphoid gene expression. Single-cell mRNA sequencing of forebrain microglia from 8-month-old wild-type (2♂) and 5xFAD mice (2♂) from Figs. 1a and 2a, Supplementary Fig. 1, and Supplementary Table 1. (**a**) Top: Isolation of microglia by fluorescence-activated cell sorting (FACS) followed by single-cell mRNA sequencing. Bottom: The balloon plot shows differentially regulated gene ontologies (GOs, Fisher’s exact test) in the PU.1^low^ and PU.1^high^ cortical disease-associated microglia (DAM) subpopulations. (**b**) Scatter plot shows the correlation of upregulated genes in cortical PU.1^high^ (red) vs. PU.1^low^ microglia (blue) between two independent experiments (n = 1 mouse/group per experiment) using FindMarkers() analysis in Seurat—Wilcoxon rank sum test. 558 genes are significantly upregulated and 445 genes are significantly downregulated in PU.1^low^ microglia in two independent experiments, while 0 genes are regulated in opposing directions. (**c**) Plaque-associated, CSF1R-independent PU.1^low^ microglia show lymphoid gene expression. Single-nuclei sequencing of cortical microglia from 6-month-old control (♀) and two CSF1Ri-treated 5xFAD mice (2♂ pooled). Left: Isolation of lineage-traced microglial nuclei from the cortex by fluorescence-activated nuclear sorting (FANS) followed by single-nuclei sequencing. Right: The volcano plot shows the differentially up- (red) and down- (blue) regulated genes in cortical microglia control and CSF1Ri-treated mice from Fig. 1d by FindMarkers() function in Seurat—Wilcoxon rank sum test. Please also see Supplementary Fig. 2 and Supplementary Table 3. (**d**) PU.1^low^ microglia-expressed lymphoid-associated signaling molecules (ligands, surface receptors, and downstream signaling proteins) in T-cells are shown. Adapted from “T-cell and B-cell signaling” and “TCR downstream signaling” generated by BioRender.com. (**e**) Heatmap shows log2 (fold change) of selected differentially expressed genes in the PU.1^low^ DAM compared to the PU.1^high^ DAM, two independent experiments: (1) single-cell sequencing of forebrain microglia from wild-type and 5xFAD mice (Figs. 1a, 2a, Extended Data Fig. 3a) and (2) single-nuclei sequencing of cortical microglia from control diet- and CSF1Ri diet-fed 5xFAD mice (Fig. 1d, Extended Data Fig. 3c). The color indicates log2 (fold change). (**f**) Top: Schematic shows multiplexed error-robust fluorescent in situ hybridization (MERFISH) within the brain of the 5xFAD model (8-month-old, ♂). Bottom: The representative image shows the identification of cells and plaques in the cortex. DAPI+ nuclei: red, 6E10+ amyloid β plaques: blue, Meg3+ neurons: brown, segmented plaques: blue dots. Scale bar: 1 mm. (**g**) Representative images show different cell types as well as a PU.1^low^ plaque-associated, and PU.1^high^ distal microglia. Macrophage-identifying gene *Mrc1*: dark blue. Endothelial cell identifying gene *Cldn5*: orange. T cell identifying genes *Cd3e* and *Cd8a*: green. Microglia homeostatic genes *Cx3cr1, Tmem119, P2ry12, Csf1r, Hexb, Aif1, Tnf, Trem2, Itgam, C1qa, C1qb, C1qc, Sall1, Selplg*: red. Neuron-identifying gene *Meg3*: pink. Oligodendrocyte-identifying genes *Mog, Sox10*, and *Mag*: brown. Lymphoid genes *Pdcd1, Cd274, Cd28, Cd72, Tnfsf13b, Cd4:* cyan. DAM identifying genes *Itgax, Clec7a, Igf1, Cst7, Spp1*: yellow. *Spi1* (PU.1): white. Amyloid β (6E10): blue. DAPI+ nuclei: gray. Marker genes are shown in Supplementary Table 4. Illustrations in **a**, **c**,** d** and **f** were created using BioRender (https://biorender.com).

The PU.1^low^ plaque-associated microglia displayed gene expression patterns that, in addition to promoting lipid metabolism and lysosomal function^37–40^ (Extended Data Fig. 3a), showed a resemblance to lymphoid lineage cells (Fig. 2 and Extended Data Fig. 3). Microglial single-cell (Fig. 2a, Extended Data Fig. 3a,b and Supplementary Table 1), lineage-traced single-nucleus (Extended Data Fig. 3c–e and Supplementary Table 3) or spatial transcriptomics analyses (Fig. 2b,c, Extended Data Fig. 3f,g, Supplementary Fig. 3 and Supplementary Table 4) revealed that PU.1^low^ plaque-associated microglia express genes encoding immunoregulatory lymphoid receptors and signalling proteins, such as CD28, PD-1 (*Pdcd1*), PD-L1 (*Cd274*), CD5, CTLA-2A, CD52, LAT2 and CD72 (Extended Data Fig. 3d). The transcripts encoding these proteins are associated with ribosomes in 5xFAD microglia (Fig. 2d, Supplementary Fig. 4 and Supplementary Table 5), suggesting their translation and protein expression. Microglial lymphoid gene expression in PU.1^low^ disease-associated microglia (DAM) is not restricted to mice but is also observed in individuals with AD^15^ (Fig. 2e and Supplementary Fig. 5).

**Fig. 3:**
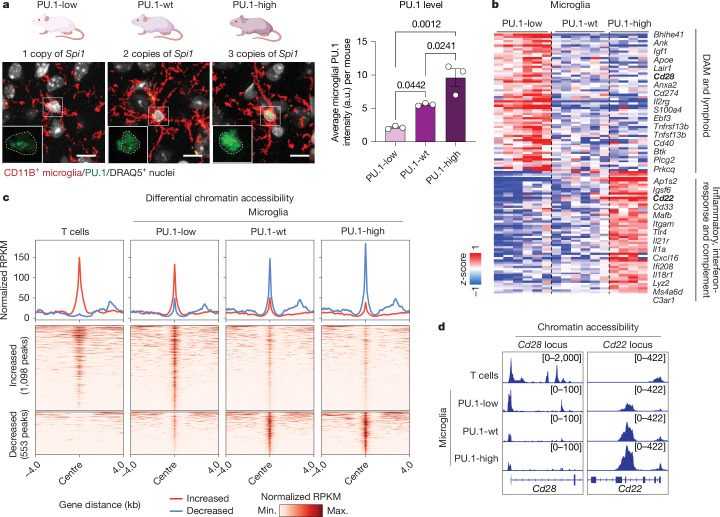
PU.1 levels regulate lymphoid gene expression in microglia. **a**, Microglial PU.1 protein expression (green) correlates with *Spi1* gene copy number in PU.1-low, PU.1-wt and PU.1-high mice. Bar graph shows the mean PU.1 intensity within the cortical microglia in 6-month-old control (*Cx3cr1*^*CreErt2/+*^; *n* = 3; one female and two males), PU.1-low (*Cx3cr1*^*CreErt2/+*^*;Spi1*^*fl/+*^; *n* = 3; one female and two males) and PU.1-high (*Cx3cr1*^*CreErt2/+*^*;R26*^*LSL.F-Spi1/+*^; *n* = 3 females) mice, as determined by ordinary one-way ANOVA with multiple comparisons. Microglial nuclei identified by CellProfiler are outlined (dotted circles). **b**, PU.1^low^ microglia upregulate lymphoid-lineage genes. The heat map shows differentially expressed genes (DESeq2; *z*-scored variance-stabilizing transformation (VST)) from cortical microglia-specific TRAP sequencing from 6-month-old PU.1-low (*n* = 6; four females and two males), PU.1-wt (*n* = 6; three females and three males) and PU.1-high (*n* = 4; three females and one male) mice. **c**,**d**, PU.1^low^ microglia display T-cell-like chromatin accessibility profiles in vivo. **c**, Profile plots (top) and heat maps (bottom) show normalized genome-wide chromatin accessibility by assay for transposase-accessible chromatin sequencing (ATAC-seq) (reads per kilobase per million mapped reads (RPKM)) from the microglia of PU.1-low, PU.1-wt and PU.1-high mice (9–10 months old; *n* = 3 males per genotype) and splenic T cells (*n* = 2 males) from wild-type mice. Data are centered on ±4 kb around differentially accessible (DESeq2) peaks in PU.1-low compared with wild-type microglia. Peaks are ranked by signal intensity using the same minimum and maximum ranges for plots and heat maps. **d**, Genome browser views (Integrative Genomics Viewer) of *Cd28* and *Cd22* gene loci in T cells versus PU.1-low, PU.1-wt and PU.1-high microglia. Bracketed numbers indicate the minimum and maximum *y*-axis values for each sample. Scale bar, 5 μm. Illustrations in **a** were created using BioRender (https://biorender.com). wt, wild type. Source data

**Extended Data Fig. 4:**
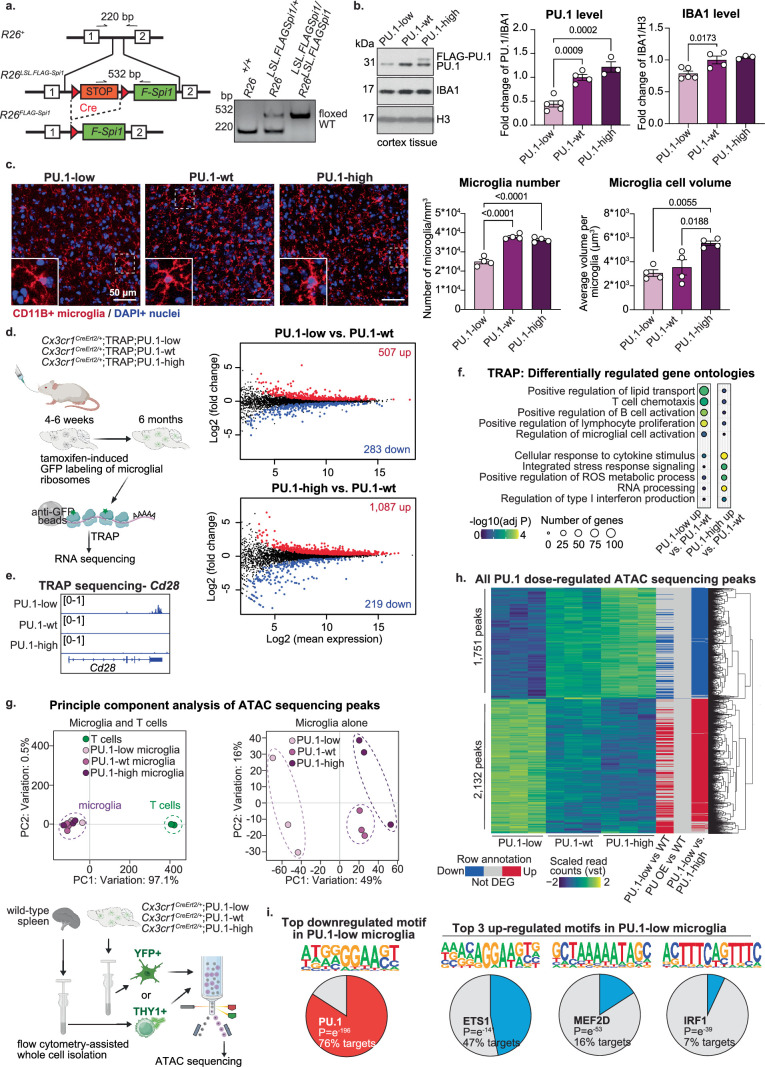
Generation of PU.1-low and PU.1-high mice (related to Fig. 3). (**a**) Gene targeting approach^110,111^ for the generation of the inducible PU.1-high *R26*^*LSL.F-Spi1/+*^ mice. Brackets indicate the two homology arms mediating recombination in embryonic stem cells. *F-Spi1* (green) encodes the FLAG-tagged PU.1 protein sequence, and *loxP* (red) sites are indicated by arrowheads. Cre-mediated removal of the *loxP-*flanked STOP codon leads to *F-Spi1* expression. Right: Representative genotyping gel images of *R26*^*+/+*^ wild-type, *R26*^*LSL.F-Spi1/+*^ heterozygous, and *R26*^*LSL.F-Spi1/LSL.F-Spi1*^ homozygous mice are shown. Independently repeated >800 times. (**b**) PU.1 protein expression in PU.1-low, PU.1-wild-type (PU.1-wt), and PU.1-high mice. Representative Western blot (left) and quantification of protein levels (right). Representative fluorescence-activated cell sorting (FACS) plots for microglia isolation are shown in Supplementary Fig. 6b. The upper band in PU.1-high mice indicates the FLAG-tagged PU.1 protein expressed from the *R26* locus. The bar graphs show PU.1 level normalized to microglial marker IBA1, IBA1 level normalized to Histone H3 in the cortex of 6-month-old PU.1-low (*Cx3cr1*^*CreErt2/+*^*;Spi1*^*fl/+*^, n = 5 mice, 3♀, 2♂), PU.1-wt (*Cx3cr1*^*CreErt2/+*^, n = 5 mice, 2♀, 3♂), and PU.1-high mice (*Cx3cr1*^*CreErt2/+*^*;R26*^*LSL.F-Spi1/+*^, n = 3 mice, 3♀)—ordinary one-way ANOVA with multiple comparisons. (**c**) PU.1-low and PU.1-high mice show changes in microglia numbers (lower in PU.1-low) and morphology (increased volume in PU.1-high) in the healthy brain. Representative immunofluorescence images (left) and quantification of microglia morphologies (right). CD11B+ microglia: red, DAPI+ nuclei: blue. The bar graphs show the number of microglia (left) and microglial cell size (volume, right) in the cortex of 6-month-old PU.1-low (n = 4 mice, 1♀, 3♂), PU.1-wt (n = 4 mice, 3♀, 1♂), and PU.1-high mice (n = 4 mice, 3♀, 1♂), as calculated by Imaris—ordinary one-way ANOVA with multiple comparisons. (**d-f**) PU.1-low and PU.1-high microglia show distinct changes in gene expression in the healthy brain, with PU.1-low inducing lymphoid and PU.1-high inducing pro-inflammatory and stress response patterns. Microglia-specific translating ribosome affinity purification (TRAP) sequencing from 6-month-old PU.1-low (n = 6 mice, 4♀, 2♂), PU.1-wt (n = 6 mice, 3♀, 3♂), and PU.1-high mice (n = 4 mice, 3♀, 1♂). (**d**) Experimental design (left) and MA plots (right, representing log-ratio (M) on the y-axis and mean average (A) on the x-axis) show gene expression changes (red, upregulated; blue, downregulated by DESeq2) shown in Supplementary Table 6. (**e**) Representative genome browser views (in Integrative Genomics Viewer, IGV) of normalized read counts of *Cd28* ribosome-associated mRNA in PU.1-low, PU.1-wt, and PU.1-high microglia. Numbers in brackets indicate the minimum and maximum values of the y-axis shown for each gene. (**f**) The balloon plot shows enrichment of gene ontologies (Fisher’s exact test) in differentially regulated genes associated with PU.1 level-dependent and independent gene sets by DESeq2. p-value (color) and combined score (size) are shown. (**g-i**) PU.1-low microglia show overall microglia-specific chromatin accessibility, with PU.1 expression levels governing distinct accessibility patterns. ATAC sequencing of microglia from PU.1-low, PU.1-wt, and PU.1-high microglia from the brain (n = 3 mice/group) and T-cells from wild-type spleen (n = 2 mice). 9- to 10-month-old ♂ mice were used. 50,000 cells used per sample. (**g**) Bottom: Isolation of PU.1-low, PU.1-wild-type (PU.1-wt), and PU.1-high microglia from the brain and wild-type T cells from the spleen by FACS, followed by ATAC sequencing. Top: Principal component analysis (PCA) of the 5,000 most differential ATAC sequencing peaks in microglia and T cells (left) or between microglia of the different PU.1 genotypes (right). (**h**) Heatmap shows all differentially up- and downregulated peaks (called by MACS2) between PU.1-low, PU.1-wt, and PU.1-high microglia identified by DESeq2 (PU.1-low vs. PU.1-wt, PU.1-high vs. PU.1-wt, and PU.1-low vs. PU.1-high). Colors in the main heatmap (left) show scaled read counts (variance stabilizing transformations, vst) per sample. Colors on the side heatmap (right) show significantly up or downregulated peaks between indicated samples shown in Supplementary Table 7. (**i**) HOMER DNA-motif enrichment analysis of peaks with reduced and increased accessibility in PU.1-low microglia identifies enrichment in peaks containing the PU.1 DNA binding motif (down), and ETS1, MEF2D, and IRF1 (up). Bar graphs with individual data points show mean ± SEM. Illustrations in **d** and **g** were created using BioRender (https://biorender.com). Source data

To test the causal role of PU.1 downregulation in mediating the transcriptional shift towards a lymphoid gene expression pattern, we engineered mice with genetically altered PU.1 expression levels in microglia. Microglia-specific Cre-dependent deletion of one *Spi1* allele^41^ (PU.1-low mice; *Cx3cr1*^*CreERT2/+*^*;Spi1*^*fl/+*^) resulted in an approximately 50% PU.1 reduction (Fig. 3a and Extended Data Fig. 4a–c). Conversely, PU.1-high mice were generated by Cre-dependent activation of an extra FLAG-tagged *Spi1* gene (*Cx3cr1*^*CreERT2/+*^*;Rosa26*^*LSL.FLAG-Spi1/+*^), showing an approximately 25% increase in PU.1 expression (Fig. 3a and Extended Data Fig. 4a–c). The changes in PU.1 expression levels were induced postnatally between 4 and 6 weeks to avoid developmental confounds.

**Extended Data Fig. 5:**
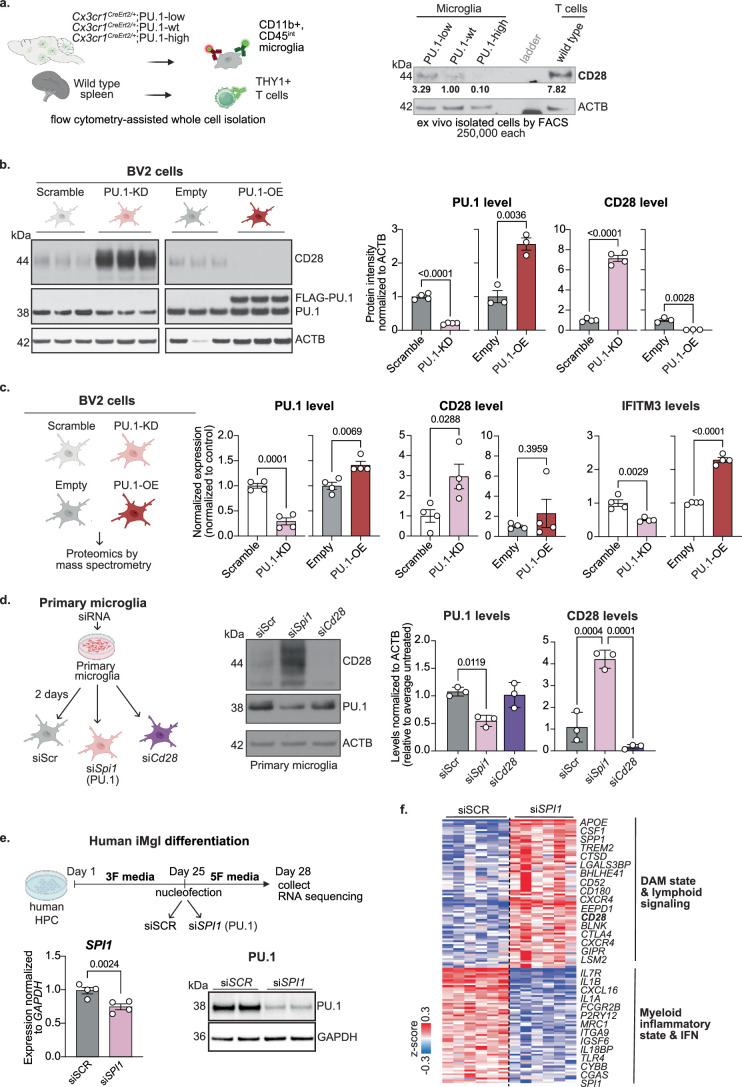
PU.1 regulates lymphoid gene expression in microglia in vivo and in vitro, in BV2 cells, in primary mouse microglia, and human iMgls (related to Fig. 3). (**a**) Microglia in vivo: Schematic showing microglia and T cell isolation (left) and Western blot with quantification for CD28 levels (normalized CD28 quantification shown below the blot) in ex vivo acutely isolated microglia from the forebrain of 12-month-old PU.1-low, PU.1-wild-type (PU.1-wt), and PU.1-high mice (n = 3 pooled mice, 1♀, 2♂ each) as well as T cells isolated from the spleen of a wild-type control (♂). Representative FACS plots for microglia isolation are shown in Supplementary Fig. 1c. Representative FACS plots for T cell isolation are shown in Supplementary Fig. 6b. (**b-c**) BV2 cells: control cells treated with scrambled small interfering (si) RNAs (scramble) and PU.1-knockdown (PU.1-KD) cells treated with siRNAs against *Spi1*, as well as control cells transfected with empty vector (empty) and PU.1-overexpressor (PU.1-OE) cells transfected with a vector containing the *Spi1* transgene from Pimenova et al.^39^. (**b**) Representative protein expression analysis using Western blot (left) with experimental design (top) and quantification (right) of CD28 and PU.1 levels in scramble and PU.1-KD cells (n = 4 wells/group), as well as in empty and PU.1-OE cells (n = 3 wells/group) are shown—unpaired two-tailed t-test was used. (**c**) Experimental design (left) and bar graphs (right) show protein levels (n = 4 wells/group) identified by proteomics analysis—unpaired two-tailed t-test. Differentially expressed proteins are shown in Supplementary Table 8. (**d**) Primary microglia: Experimental design (left), representative Western blot (middle), and quantification (right) of CD28 and PU.1 levels in primary microglia treated with siRNAs against *Spi1* (PU.1) or *Cd28* or control (scramble, Scr, n = 3 wells/group)—ordinary one-way ANOVA with multiple comparisons used. (**e-f**) Human iMgls: (**e**) Experimental design (top), RNA expression quantification by qPCR (bottom left), and representative protein expression analysis using Western blot (bottom right) of *SPI1*/PU.1 level in human hematopoietic progenitor cell-(HPC)-derived microglial-like cells (iMgls) treated with siRNAs against *SPI1*/PU.1 (si*SPI1*) or control scramble siRNA (siSCR). The bar graph shows qPCR-based quantification of *SPI1* normalized to *GAPDH* in siSCR and si*SPI1-*targeted iMgls (n = 4 independent experiments)—paired two-tailed t-test. Please see Table 2 for demographic information. (**f**) Heatmap shows z-scored vsts of the differentially regulated genes in siSCR and si*SPI1-*targeted iMgls identified by RNA sequencing and DESeq2 (n = 6 independent experiments), shown in Supplementary Table 9. Bar graphs with individual data points show mean ± SEM. Illustrations in **a**–**e** were created using BioRender (https://biorender.com). Source data

**Extended Data Fig. 6:**
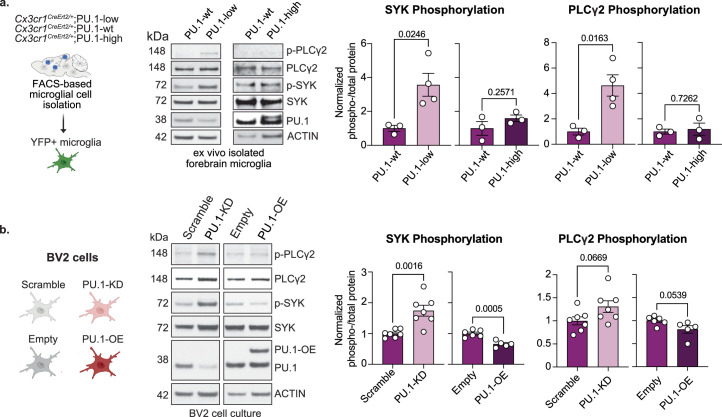
Downregulation of PU.1 in mouse microglia and BV2 cells is associated with an increase in SYK/PLCγ2 signaling (related to Fig. 3). (**a, b**) Experimental design (left), representative protein analysis by Western blot (middle), and quantification (right). The bar graph shows SYK and PLCγ2 phosphorylation levels normalized to total SYK and PLCγ2 protein levels—unpaired two-tailed t-test. (**a**) ex vivo microglia: Microglia acutely isolated from the forebrain of 6-month-old PU.1-wild-type (PU.1-wt, n = 3 mice, 3♀) and PU.1-low (n = 4 mice, 4♀); as well as PU.1-wt (n = 3 mice, 2♀, 1♂), and PU.1-high mice (n = 3 mice, 1♀, 2♂). Protein extracts equivalent to 120,000 microglia were loaded for Western blot per sample. Representative FACS plots for microglia isolation are shown in Supplementary Fig. 6b. (**b**) BV2 cells: PU.1-knockdown (PU.1-KD) cells treated with small interfering (si) RNAs against *Spi1* with associated control cells treated with scrambled siRNAs (scramble), as well as PU.1-overexpressor (PU.1-OE) cells transfected with vector containing *Spi1* transgene with associated control cells transfected with empty vector (empty) from Pimenova et al.^39^. n = 7 wells in scramble and PU.1-KD; n = 6 wells in empty, and n = 5 wells in PU.1-OE—pooled from two independently performed experiments. Bar graphs with individual data points show mean ± SEM. Illustrations in **a** and **b** were created using BioRender (https://biorender.com). Source data

**Extended Data Fig. 7:**
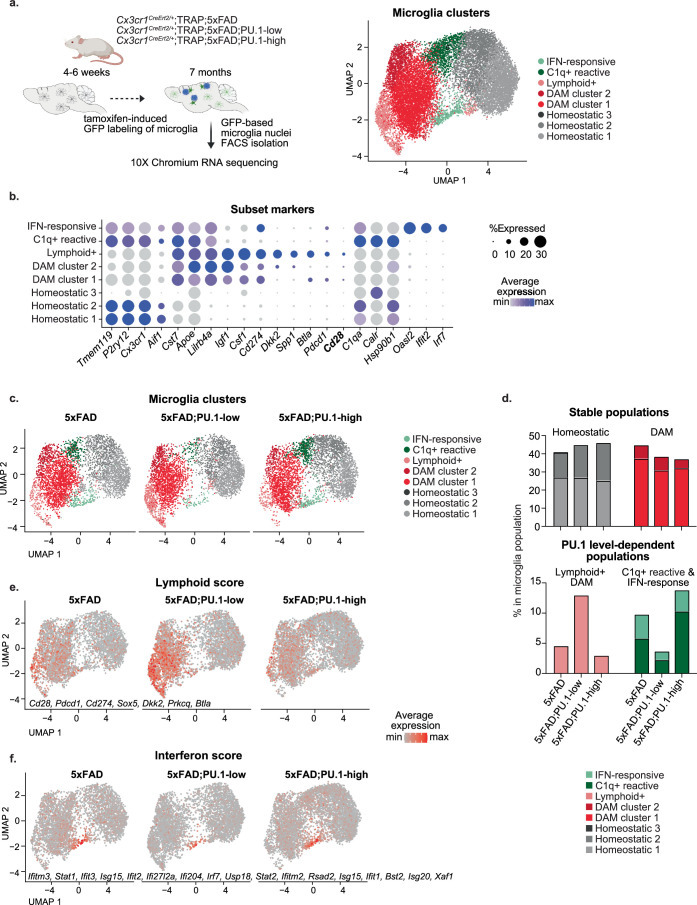
PU.1 levels govern microglia transcriptional responses in 5xFAD mice: microglia single nuclei analysis (related to Fig. 4). (**a-f**) Single-nuclei mRNA sequencing of cortical microglia from a 6-month-old 5xFAD (♀), 5xFAD;PU.1-low (♂), and 5xFAD;PU.1-high mouse (♂). (**a**) Left: Isolation of lineage-traced microglial nuclei by fluorescence-activated nuclear sorting (FANS) followed by single-nuclei sequencing of cortical microglia from 6-month-old control, 5xFAD, and 5xFAD;PU.1-low mice. Please see Supplementary Fig. 2b for the gating strategy. Right: Uniform manifold approximation and projection (UMAP) visualizations show microglia assigned into clusters that are manually annotated based on the marker genes. (**b**) The dot plot shows the scaled average expression per cluster (color) and the percentage of cells (size) from each DAM subcluster. (**c**) UMAP visualizations show microglia assigned into clusters that are manually annotated based on the marker genes shown in Supplementary Table 10. (**d**) The bar graphs show the change in the percentage of different microglia subpopulations in the three genotypes. (**e, f**) Feature plots show the expression of indicated lymphoid (e) and interferon genes (f) and the distribution of cells expressing the genes. Illustrations in **a** were created using BioRender (https://biorender.com). Source data

Reduction of PU.1 expression in microglia was sufficient to recapitulate the lymphoid gene signature observed in plaque-associated PU.1^low^ microglia, even in the absence of amyloid pathology (Fig. 3b, Extended Data Fig. 4d–f and Supplementary Table 6). Moreover, PU.1^low^ microglia, although maintaining an overall microglial lineage-specific chromatin accessibility pattern (Extended Data Fig. 4g, Supplementary Fig. 6 and Supplementary Table 7), showed more T cell-like accessibility changes (Fig. 3c,d, Extended Data Fig. 4g–i, Supplementary Fig. 6 and Supplementary Table 7). This transcriptional shift towards lymphoid gene (Fig. 3b, Extended Data Fig. 4d–f and Supplementary Table 6) and protein expression (Extended Data Fig. 5), which is associated with an increase in microglial SYK–PLCγ2 signalling capacity (Extended Data Fig. 6), can be triggered in vitro by acute PU.1 knockdown through RNA interference in microglia-like BV2 cells^39^ (Extended Data Fig. 5b,c and Supplementary Table 8), primary mouse microglia (Extended Data Fig. 5d) or human induced pluripotent stem cell (iPSC)-derived microglia (induced microglia-like cells (iMgls); Extended Data Fig. 5e,f and Supplementary Tables 2 and 9). These data support a role for reduced PU.1 expression in a microglial shift towards lymphoid-associated gene expression in mice and humans. By contrast, increased PU.1 expression in microglia is associated with a pro-inflammatory phenotype^38,39^ both in the non-diseased brain (Fig. 3b, Extended Data Figs. 4d–i and 5c and Supplementary Tables 6 and 8) and in response to amyloid pathology (multiplexed error-robust fluorescence in situ hybridization (MERFISH), Supplementary Fig. 7 and Supplementary Table 4; single-cell sequencing, Extended Data Fig. 7 and Supplementary Table 10).

**Fig. 4:**
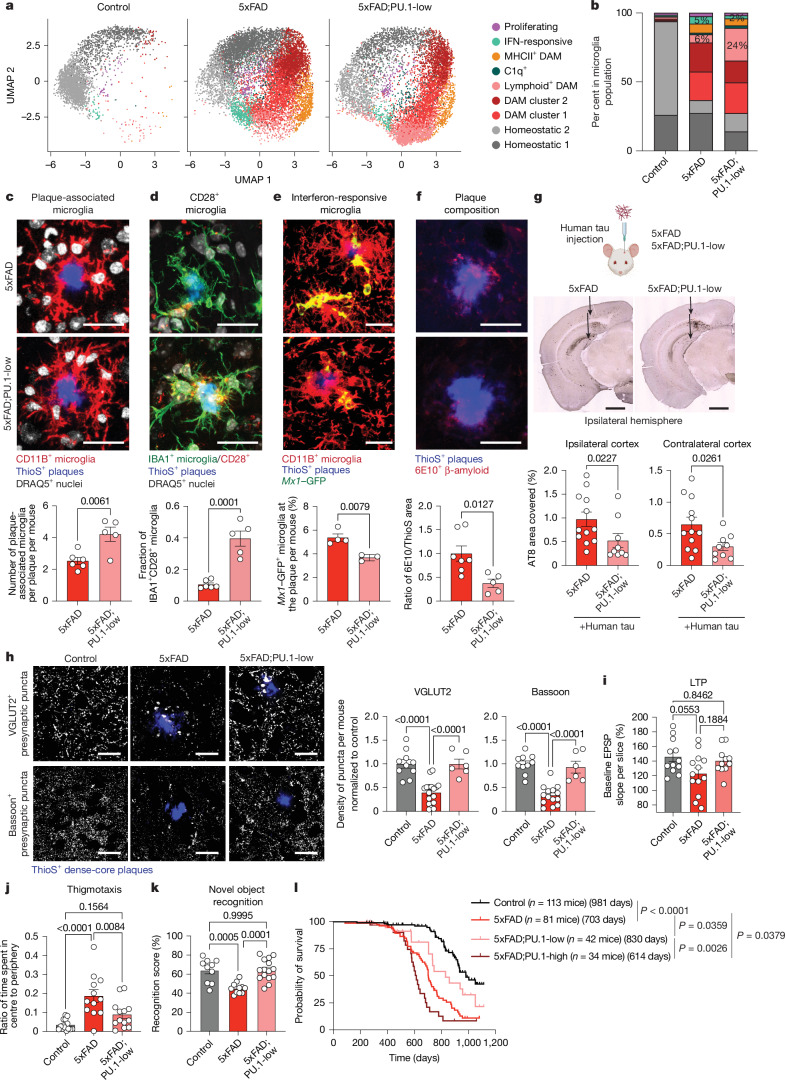
PU.1^low^ microglia exhibit neuroprotective phenotypes. **a**,**b**, UMAP (**a**) and bar graph (**b**) show microglial subpopulations and their distribution on the basis of MERFISH data in wild-type (*n* = 2 males), 5xFAD (*n* = 3 males) and 5xFAD–PU.1-low (*n* = 2 males) mice (8–10 months old). **c**–**f**, Representative cortical immunofluorescence and quantification in 6-month-old mice, analysed using an unpaired two-tailed *t*-test. **c**, Plaque-associated microglia in 5xFAD (*n* = 6; three females and three males) and 5xFAD–PU.1-low (*n* = 5; three females and two males) mice. **d**, CD28^+^ microglia in 5xFAD (*n* = 6; three females and three males) and 5xFAD–PU.1-low (*n* = 5; four females and one male) mice. **e**, MX1–GFP^+^ microglia in 5xFAD;*Mx1*–GFP (*n* = 4; two females and two males) and 5xFAD–PU.1-low;*Mx1*–GFP (*n* = 3; two females and one male) mice. **f**, Plaque composition (6E10^+^ soluble amyloid-β relative to ThioS^+^ dense-core plaques) in the subiculum of 5xFAD (*n* = 7; two females and five males) and 5xFAD–PU.1-low (*n* = 5; two females and three males) mice. **g**, Seeding of AT8^+^ phosphorylated Tau (p-Tau) in 10-month-old mouse brains injected with human Tau aggregates. Bar graphs show percentage of AT8^+^ area in 5xFAD (*n* = 12; seven females and five males) and 5xFAD–PU.1-low (*n* = 9; four females and five males) mice, analysed using an unpaired two-tailed *t*-test. **h**–**k**, Synaptic analyses were performed in 6-month-old mice, and physiological and behavioural analyses were performed in 9–10-month-old mice, using ordinary one-way ANOVA with multiple comparisons. **h**, VGLUT2 and Bassoon protein density in layer V cortex of control (*n* = 10; four females and six males), 5xFAD (VGLUT2 (*n* = 17; eight females and nine males); Bassoon (*n* = 14; six females and eight males)) and 5xFAD–PU.1-low (*n* = 6; four females and two males) mice. **i**, Late-phase LTP in CA1 hippocampal neurons of control (*n* = 12 slices from five mice; three females and two males), 5xFAD (*n* = 13 slices from six mice; four females and two males) and 5xFAD–PU.1-low (*n* = 11 slices from five mice; one female and four males) mice. **j**, Open-field thigmotaxis (centre/periphery) in control (*n* = 15; nine females and six males), 5xFAD (*n* = 12; six females and six males) and 5xFAD–PU.1-low (*n* = 13; seven females and six males) mice. **k**, Novel object recognition scores in control (*n* = 10; five females and five males), 5xFAD (*n* = 11; six females and five males) and 5xFAD–PU.1-low (*n* = 15; seven females and eight males) mice. **l**, Survival curves for wild-type (*n* = 113; 49 females and 64 males), 5xFAD (*n* = 81; 38 females and 43 males), 5xFAD–PU.1-low (*n* = 42; 19 females and 23 males) and 5xFAD–PU.1-high (*n* = 34; 22 females and 12 males) mice, analysed using Kaplan–Meier curve with a simple log-rank Mantel–Cox test. Bar graphs show the mean ± s.e.m. with individual points. Scale bars, 20 μm (**c**–**f**,**h**), 1 mm (**g**). Illustration in **g** was created using BioRender (https://biorender.com). Source data

**Extended Data Fig. 8:**
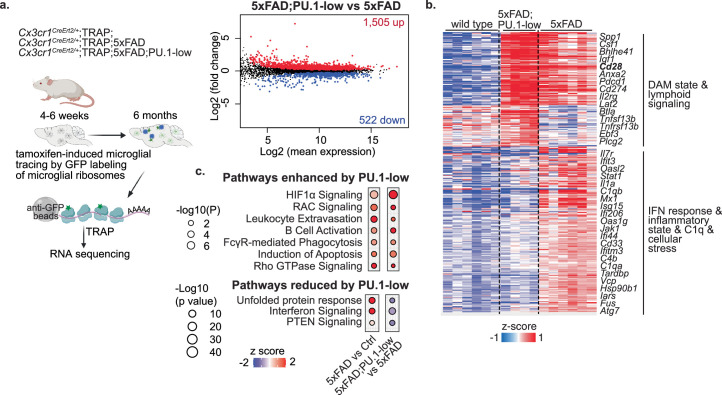
PU.1 levels regulate microglia transcriptional responses in 5xFAD mice: microglia-specific TRAP analysis (related to Fig. 4). (**a-c**) Microglia-specific translating ribosome affinity purification (TRAP) sequencing from 6-month-old control (n = 6 mice, 3♀, 3♂), 5xFAD (n = 6 mice, 3♀, 3♂), and 5xFADPU.1-low mice (1♀, 3♂). (**a**) Experimental design (left) and MA plots (right, representing log-ratio (M) on the y-axis and mean average (A) on the x-axis) show gene expression changes (red, upregulated; blue, downregulated) by DESeq2, shown in Supplementary Table 11. (**b**) Heatmap shows z-scored variance stabilizing transformations (vsts, color) of genes selected by overlapping and non-overlapping genes between pairwise comparisons (5xFAD;PU.1-low vs. 5xFAD and 5xFAD vs. control). (**c**) The balloon plot shows significantly enriched gene ontologies (Fisher’s exact test) associated with significantly regulated PU.1 level-dependent and independent gene sets (DESeq2). p-value (color) and combined score (size) are shown. Illustrations in **a** were created using BioRender (https://biorender.com).

**Extended Data Fig. 9:**
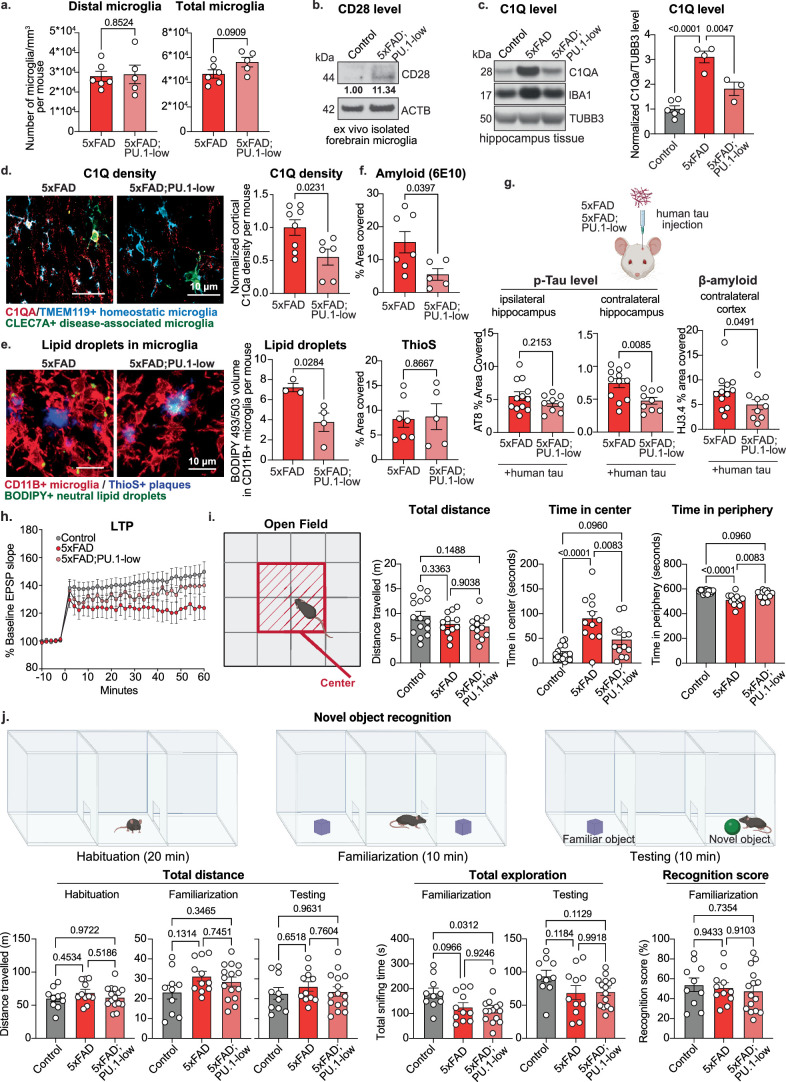
PU.1^low^ microglia display neuroprotective phenotypes and reduce amyloid load and Tau spreading in the brain of 5xFAD mice (related to Fig. 4). (**a**) 5xFAD-PU.1-low mice show similar numbers of distal microglia. The bar graphs show the density of distal and total microglia in the cortex of 6-month-old 5xFAD (n = 6 mice, 3♀, 3♂) and 5xFAD-PU.1-low mice (n = 5 mice, 3♀, 2♂)—unpaired two-tailed t-test. (**b**) CD28 protein expression. Western blot shows CD28 levels in ex vivo microglia acutely isolated from the forebrain of 12-month-old control (n = 3 pooled mice, 1♀, 2♂) and 5xFAD; PU.1-low mice (n = 3 pooled mice,1♀, 2♂). Normalized CD28 quantification is shown below the blot. (**c-e**) Microglia in 5xFAD-PU.1-low mice show reduced neutral lipid droplet load and complement C1Q expression. (**c**) Western blot (left) with quantification (right). The bar graph shows the C1QA level normalized to TUBB3 per sample in the hippocampi of 6-month-old control (n = 6 mice, 6♂), 5xFAD (n = 4 mice, 4♂), and 5xFAD-PU.1-low mice (n = 3 mice, 3♂)—ordinary one-way ANOVA with multiple comparisons was used. (**d-e**) Representative immunofluorescence images (left) with quantification (right) from the cortex of 6-month-old mice—unpaired two-tailed t-test. Scale bar: 10 μm. (**d**) CLEC7A+ disease-associated microglia: green, TMEM119+ homeostatic microglia: cyan, C1QA: red. The bar graph shows the percent C1QA in the cortex of 5xFAD (n = 8 mice, 5♀, 3♂) and 5xFAD-PU.1-low mice (n = 6 mice, 4♀, 2♂). (**e**) CD11B+ microglia: red, BODIPY 493/503: green, ThioS+ dense-core plaques: blue. The bar graph shows the BODIPY+ volume within microglia normalized to microglia volume in 5xFAD (n = 3 mice, 2♀, 1♂) and 5xFAD-PU.1-low mice (n = 4 mice, 2♀, 2♂). (**f**) 5xFAD-PU.1-low mice show a reduced soluble amyloid load. The bar graphs show the percent area covered by 6E10+ soluble Aβ or ThioS+ dense-core plaques in the subiculum from 6-month-old 5xFAD (n = 7 mice, 2♀, 5♂) and 5xFAD; PU.1-low mice (n = 5 mice, 2♀, 3♂)—unpaired two-tailed t-test. Representative images are shown in Fig. 4f. (**g**) 5xFAD-PU.1-low mice show a reduced spread of p-Tau following intracerebral injection of human pathogenic Tau isolated from the brain (schematic, top). Bar graphs (bottom) show the percent AT8+ phosphorylated Tau (p-Tau) area and HJ3.4+ amyloid β in the indicated brain regions of 10-month-old 5xFAD (n = 12 mice, 7♀, 5♂) and 5xFAD; PU.1-low mice (n = 9 mice, 4♀, 5♂) (**h**) LTP: The line graph shows the time course of long-term synaptic plasticity (LTP) quantified as a percentage change in excitatory postsynaptic potential (EPSP) slope from baseline (10-min period of a pre-and 60-min period of a post-100 Hz stimulation) in CA1 neurons of the hippocampus in 9-10-month-old control (n = 12 slices from 5 mice, 3♀, 2♂), 5xFAD (n = 13 slices from 6 mice, 4♀, 2♂), and 5xFAD; PU.1-low mice (n = 11 slices from 5 mice, 1♀, 4♂)—two-way ANOVA with repeated measures, P_time_ = 0.0183, P_genotype_ = 0.1031. (**i**) Open field analysis: Experimental design (left) and quantification (right) of the open field experiment. The area identified as “center” is marked by a red square. The bar graph shows the total distance traveled (m), time spent in the center (seconds), and the periphery (seconds) in a 10-min-long open field assay from 9–10 month-old control (n = 15 mice, 9♀, 6♂), 5xFAD (n = 12 mice, 6♀, 6♂), and 5xFAD; PU.1-low (n = 13 mice, 7♀, 6♂)—ordinary one-way ANOVA with multiple comparisons. (**j**) Novel object recognition: The schematic (top) and quantification of the novel object recognition experiment in a 3-room box. The bar graphs show the distance traveled during 20-min habituation, 10-min training, and 10-min testing; the total time spent sniffing any object during training and testing; and the “recognition score” calculated as the percent time spent sniffing the constant object to the total time spent sniffing either object during training from 9-10-month-old control (n = 10 mice, 5♀, 5♂), 5xFAD (n = 11 mice, 6♀, 5♂), and 5xFAD; PU.1-low mice (n = 15 mice, 7♀, 8♂)—ordinary one-way ANOVA with multiple comparisons. No sex differences are observed in behavioral analyses, and trends between genotypes were preserved in sex-specific analyses. Bar graphs with individual data points show mean ± SEM. Illustrations in **g**, **i** and **j** were created using BioRender (https://biorender.com). Source data

Lowering PU.1 in the context of amyloid pathology (5xFAD; PU.1-low mice) has a protective effect on disease progression; it enhances microglia–plaque interactions^42^ by nearly doubling the number of plaque-associated and quadrupling the number of lymphoid gene-expressing/CD28^+^ microglia (MERFISH (Fig. 4a,b, Extended Data Fig. 3f,g, Supplementary Figs. 3 and 7 and Supplementary Table 4), single-nucleus RNA sequencing (RNA-seq) (Extended Data Fig. 7 and Supplementary Table 10), TRAP sequencing (Extended Data Fig. 8 and Supplementary Table 11), morphological analysis (Fig. 4c and Extended Data Fig. 9a) and protein analysis (Fig. 4d and Extended Data Fig. 9b)). These microglia display phenotypes linked to reduced neurotoxic activity^10,43,44^, marked by decreased expression of type I interferon-stimulated genes (such as *Mx1*) and complement (such as *C1q*), at both RNA (MERFISH (Supplementary Fig. 7 and Supplementary Table 4), single-nucleus RNA-seq (Extended Data Fig. 7 and Supplementary Table 10) and TRAP sequencing (Extended Data Fig. 8 and Supplementary Table 11)) and protein (Fig. 4e and Extended Data Fig. 9c,d) levels, as well as a decline in neutral lipid droplet accumulation (Extended Data Fig. 9e). PU.1^low^ microglia abundance was further associated with decreased amyloid burden and enhanced plaque compaction (Fig. 4f and Extended Data Fig. 9f,g). In addition, PU.1^low^ microglia are more effective in blocking the spread of injected human Tau aggregates in the brain of 5xFAD mice, leading to an overall reduction in pathogenic Tau accumulation (Fig. 4g and Extended Data Fig. 9g).

PU.1^low^ microglia preserved synaptic integrity and associated cognitive function. Although 5xFAD mice were characterized by a significant loss of presynaptic neuronal terminals (Bassoon^+^ and VGLUT2^+^) in layer V of the cortex (Fig. 4h), impaired synaptic plasticity (long-term potentiation (LTP)) in the hippocampus (Fig. 4i and Extended Data Fig. 9h) and behavioural deficits in the open-field and novel object recognition tasks^45–47^ (Fig. 4j,k and Extended Data Fig. 9i,j), lowering PU.1 level in microglia was sufficient to prevent these disease-associated alterations and preserve near-wild-type-like phenotypes (Fig. 4h–k and Extended Data Fig. 9h–j). PU.1^low^ microglia, in contrast to PU.1^high^ microglia, also significantly extended the lifespan of 5xFAD mice (Fig. 4l).

**Fig. 5:**
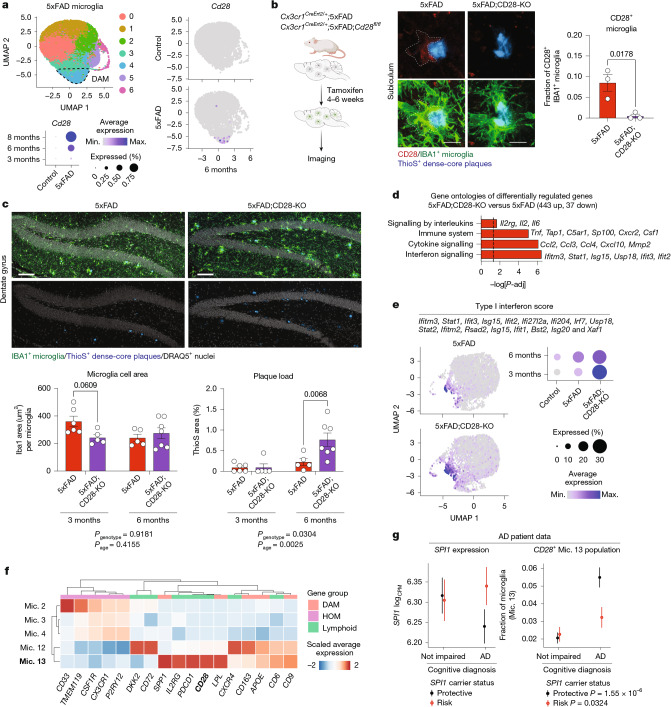
Microglial CD28 regulates inflammatory activation and amyloid plaque burden in 5xFAD mice. **a**,UMAPs show CD28^+^ microglia in 5xFAD DAM. Single-cell RNA-seq of forebrain microglia from 3-month-old, 6-month-old and 8-month-old control and 5xFAD mice (3 months (*n* = 1 control and *n* = 2 5xFAD), 6 months (*n* = 1 per group) and 8 months (*n* = 2 per group); all males); DAM outlined (dotted line). Dot and feature plots show microglial *Cd28* expression. **b**, Inducible microglia-specific deletion of CD28 in 5xFAD mice. Representative images and quantification of CD28^+^ microglia in 6-month-old 5xFAD (*n* = 3; one female and two males) and 5xFAD;CD28-KO (5xFAD;*Cx3cr1*^*CreErt2/+*^*;Cd28*^*fl/fl*^; *n* = 3; two females and one male) mice were analysed using an unpaired two-tailed *t*-test. **c**–**e**, Microglial CD28 influences plaque load and microglial activation. **c**, Microglial area and plaque density (% ThioS) in dentate gyrus of 3-month-old and 6-month-old 5xFAD (3 months (*n* = 6; one female and five males) and 6 months (*n* = 5; two females and three males)) and 5xFAD;CD28-KO mice (3 months (*n* = 5; two females and three males) and 6 months (*n* = 7; four females and three males) were analysed using two-way ANOVA with multiple comparisons. **d**, Single-cell sequencing of forebrain microglia from 5xFAD and 5xFAD;CD28-KO mice. Significant gene ontologies (Fisher’s exact test) of differentially regulated genes using pseudobulk analysis (Wilcoxon rank-sum test) in 3- and 6-month-old mice by genotype (*n* = two 3-month-old male mice and *n *= one 6-month-old male mouse per group). **e**, UMAP (two 3-month-old male mice per group) and dot plots (control and 5xFAD (as in **a**) and 5xFAD;CD28-KO (two 3-month-old male mice and one 6-month-old male mouse) show expression of interferon-response genes. **f**,**g**, Human PU.1-lowering allele associates with increased lymphoid gene-expressing microglia. **f**, The heat map shows scaled average expression of indicated genes in surveillance clusters (Mic. 2, 3 and 4) and lipid-associated/DAM clusters (Mic. 12 and 13) in human microglia^17^. **g**, PU.1 protective allele carriers have lower *SPI1* expression, whereas risk allele carriers show higher *SPI1* expression in AD microglia. Normalized *SPI1* expression and fraction of *CD28*^*+*^ Mic. 13 population per individual versus cognitive diagnosis and PU.1 variant (rs1057233 A/A = risk (*n* = 61 not impaired; *n* = 64 AD); G/A and G/G = protective (*n* = 83 not impaired; *n* = 98 AD)) were analysed using two-way ANOVA with Tukey post hoc test. Bar graphs with individual data points show the mean ± s.e.m. Scale bars, 10 μm (**b**), 100 μm (**c**). Illustrations in **b** were created using BioRender (https://biorender.com). Mic., microglia. Source data

**Extended Data Fig. 10:**
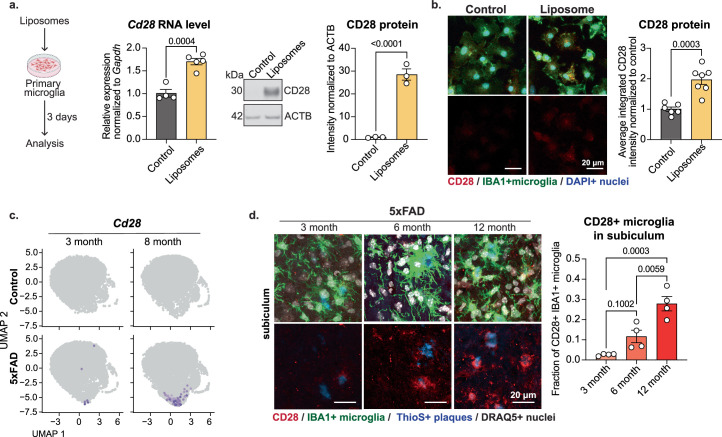
Plaque-associated signals induce microglial CD28 RNA and protein expression (related to Fig. 5). (**a-b**) Liposomes induce microglial CD28 RNA and protein expression in primary microglia in vitro: (**a**) Experimental design (left), qPCR quantification (middle left), representative Western blot (middle right), and Western blot quantification (right) of CD28 levels in primary microglia treated with 0.1 mg/ml liposomes for 3 days. The first bar graph shows *Cd28* normalized to *Gapdh* in primary microglia treated with vehicle (n = 4 wells) or liposomes (n = 5 wells)—unpaired two-tailed t-test. The second bar graph shows CD28 levels normalized to ACTB in primary microglia treated with vehicle or liposomes (n = 3 wells/group)—unpaired two-tailed t-test. (**b**) Representative immunofluorescence images (left) with quantification (right) of CD28 in primary microglia treated with 0.1 mg/ml liposomes for 3 days. IBA1+ microglia: green, CD28: red, DAPI+ nuclei: blue. Scale bar: 20 μm. The bar graphs show normalized integrated CD28 intensity in liposome-treated (control: n = 6 wells, liposome: n = 7 wells) primary microglia—unpaired t-test. (**c-d**) The number of plaque-associated CD28+ cells increases with amyloid disease progression. (**c**) *Cd28* RNA: Feature plots (right) show *Cd28* expression in uniform manifold approximation and projection (UMAP) of single-cell sequencing of forebrain microglia from 3- and 8-month-old wild-type and 5xFAD mice (n = 2 mice/group except control at 3 months: n = 1 mouse, all ♂). (**d**) CD28 protein: Representative immunofluorescence images (left) with quantification (right) of CD28+ microglia in the subiculum. IBA1+ microglia: green, CD28: red, ThioS+ dense-core plaques: blue, DRAQ5+ nuclei: gray. Scale bar: 20 μm. The bar graphs show the fraction of CD28 + IBA1+ microglia in the subiculum of 3-month-old (n = 4 mice, 2♀, 2♂), 6-month-old (n = 4 mice, 1♀, 3♂), and 12-month-old (n = 4 mice, 3♀, 1♂) 5xFAD mice—ordinary one-way ANOVA with multiple comparisons. Bar graphs with individual data points show mean ± SEM. Illustrations in **a** were created using BioRender (https://biorender.com). Source data

**Extended Data Fig. 11:**
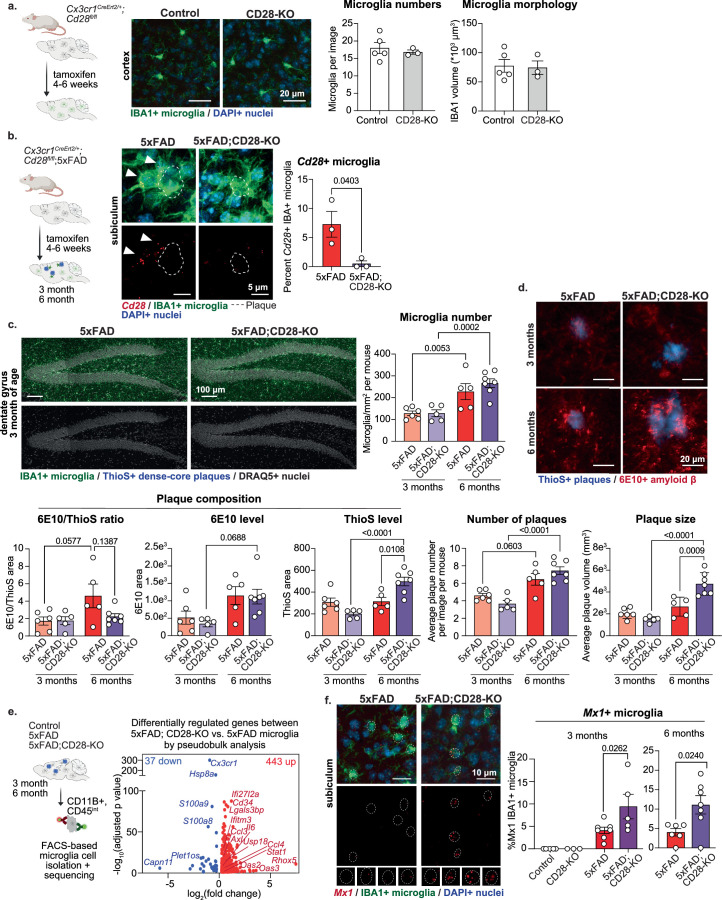
Microglial CD28 regulates amyloid burden in the brain of 5xFAD mice (related to Fig. 5). (**a**) Microglial *Cd28* ablation in the healthy brain has no impact on microglia numbers or morphology. The experimental design (left), representative immunofluorescence images (middle), and quantification (right) of microglia in the cortex of CD28-KO mice. CD11B+ microglia: green, DAPI+ nuclei: blue. Scale bar: 20 μm. The bar graphs show the total microglia numbers per image (left) and microglial cell volume (right, μm^3^) in the subiculum of 3-month-old control (n = 5 mice, 1♀, 4♂) and CD28-KO mice (*Cx3cr1*^*CreErt2/+(Litt)*^*; Cd28*^*fl/fl*^, n = 3 mice, 3♂)—unpaired two-tailed t-test. (**b**) Microglia-specific *Cd28* ablation in 5xFAD mice prevents plaque-induced *Cd28* expression. Experimental design (left), representative RNA in situ hybridization with immunofluorescence images (middle), and quantification (right) of *Cd28*+ microglia. CD11B+ microglia: green, *Cd28* mRNA: red, DAPI+ nuclei: blue. Scale bar: 5 μm. The bar graph shows the percent of *Cd28* + IBA1+ microglia in the subiculum of 6-month-old 5xFAD (n = 3 mice, 1♀, 2♂) and 5xFAD;CD28-KO mice (n = 3 mice, 2♀, 1♂)—unpaired two-tailed t-test. (**c**) Microglial *Cd28* ablation in 5xFAD mice does not affect microglia numbers. Representative immunofluorescence images (left) with quantification (right) of microglia numbers. IBA1+ microglia: green, ThioS+ dense-core plaques: blue, DRAQ5+ nuclei: gray. The bar graph shows the microglia numbers in the dentate gyrus of 3- and 6-month-old 5xFAD (n = 6 mice at 3 months, 1♀, 5♂ and n = 5 mice at 6 months, 2♀, 3♂) and 5xFAD; CD28-KO mice (n = 5 mice at 3 months, 2♀, 3♂ and n = 7 mice at 6 months, 4♀, 3♂)—two-way ANOVA with multiple comparisons. P_genotype_ = 0.3913, P_age_ < 0.0001 (**d**) Microglial *Cd28* ablation in 5xFAD mice alters plaque load and composition. Representative immunofluorescence images (left) with quantification (right) of plaque load in the subiculum. 6E10+ amyloid beta: red, ThioS+ dense-core plaques: blue. The bar graphs show 6E10/ThioS ratio, total 6E10 level, and total ThioS level, number of ThioS+ plaques, and volume of ThioS+ plaques in the dentate gyrus of 3- and 6-month-old 5xFAD (n = 6 mice at 3 months, 1♀, 5♂ and n = 5 mice at 6 months, 2♀, 3♂) and 5xFAD; CD28-KO mice (n = 5 mice at 3 months, 2♀, 3♂ and n = 7 mice at 6 months, 4♀, 3♂)—two-way ANOVA was used (ThioS: P_genotype_ = 0.3077, P_age_ = 0.0005; 6E10: P_genotype_ = 0.6140, P_age_ = 0.0024; ThioS/6E10: P_genotype_ = 0.1121, P_age_ = 0.0271; average ThioS plaque numbers per image per mouse: P_genotype_ = 0.9603, P_age_ < 0.0001; ThioS volume per image per animal: P_genotype_ = 0.0273, P_age_ < 0.0001). (**e**) Single-cell sequencing of CD45^low^ CD11B+ forebrain microglia from 3- and 6-month-old wild-type, 5xFAD, and 5xFAD;CD28-KO mice (control: n = 1 at 3 and 6 months, 5xFAD and 5xFAD;CD28-KO: n = 2 mice at 3 months, 5xFAD and 5xFAD;CD28-KO: *n* = 1 mice at 6 months, all ♂). Left: Microglia isolation strategy for single-cell sequencing. Right: Volcano plot shows significantly up- (red) and downregulated (blue) genes between all microglia from 5xFAD and 5xFAD;CD28-KO mice identified by DESeq2 in pseudobulk analysis using FindMarkers() function using Wilcoxon Rank Sum test in Seurat, shown in Supplementary Table 12. (**f**) Representative RNA in situ hybridization with immunofluorescence images (left) with quantification (right) of *Mx1* levels in microglia. IBA1+ microglia: green, *Mx1* RNA: red, DAPI+ nuclei: blue. Scale bar: 10 μm. Dotted circles indicate microglial nuclei, some of which are shown in higher magnifications on the bottom. The bar graphs show the fraction of *Mx1* + , IBA1+ microglia in the subiculum of 3-month-old wild-type (n = 5 mice, 1♀, 4♂), CD28-KO (n = 3 mice, 3♂), 5xFAD (n = 7 mice, 2♀, 5♂) and 5xFAD; CD28-KO mice (n = 5 mice, 2♀, 3♂)—two-way ANOVA was used P_disease_ = 0.0004, P_genotype_ = 0.1004—and 6-month-old 5xFAD (n = 6 mice, 3♀, 3♂) 5xFAD; CD28-KO (n = 7 mice, 4♀, 3♂)—unpaired two-tailed t test. Bar graphs with individual data points show mean ± SEM. Illustrations in **a**, **b** and **e** were created using BioRender (https://biorender.com). Source data

The PU.1^low^ microglial expression of lymphoid receptor proteins with potent signalling capacity raised a question about the contributions of these receptors to PU.1^low^ microglia activity and neuroprotection. We demonstrated that CD28, a well-known T cell co-stimulatory signalling protein^3,4^, which is induced by PU.1 downregulation and in response to plaque-associated cues in mouse (Figs. 2a,d, 3b,d and 4d and Extended Data Figs. 4e, 5, 9b and 10a,b) and human microglia (Fig. 2e), contributes to microglial activation during AD. We found that despite being expressed in only a small fraction of total microglia in 5xFAD brains (Fig. 5a, Extended Data Fig. 10c,d and Supplementary Table 12), CD28 plays an important regulatory role in broader microglia activation and disease progression. Microglia-specific deletion of CD28 in 5xFAD mice (Fig. 5b and Extended Data Fig. 11a,b) increased amyloid plaque burden (Fig. 5c and Extended Data Fig. 11c,d) and microglial activation, characterized by a broad pro-inflammatory type I interferon response in approximately 30% of all microglia (Fig. 5d,e, Extended Data Fig. 11e,f, Supplementary Fig. 8 and Supplementary Table 12). The frequency of microglia that display an inflammatory response during early disease progression exceeds the number of cells that express *Cd28* by more than 10-fold (Fig. 5a,e). One interpretation of the observed broad inflammatory microglia activation is a possible *trans*-regulatory role for CD28^+^ PU.1^low^ microglia in restraining neuroinflammation^48^ and associated amyloid plaque propagation.

**Extended Data Fig. 12:**
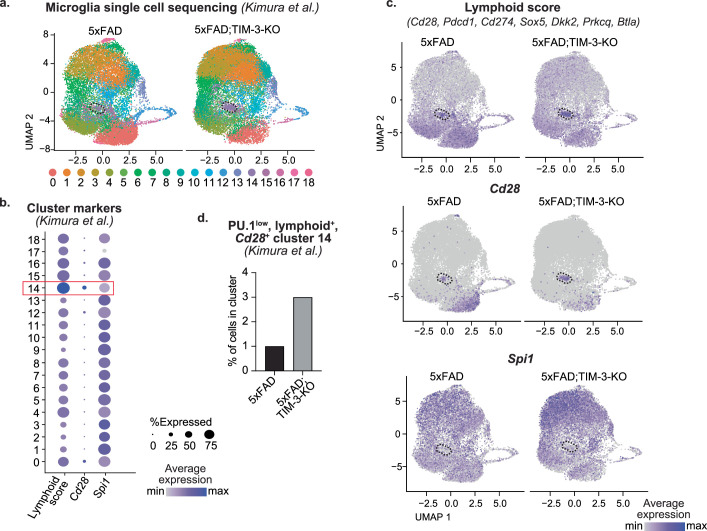
The neuroprotective PU.1^low^, lymphoid+/CD28+ microglia subpopulation expands in TIM-3-KO mice (related to Fig. 5). (**a-d**) Single-cell sequencing of human microglia from Kimura et al.^49^. (**a**) Uniform manifold approximation and projection (UMAP) visualizations (left). (**b, c**) Dot plot (b) and feature plots (c) show the expression of *Cd28*, *Spi1*, and selected PU.1^low^ microglia expressed lymphoid genes (*Cd28, Pdcd1, Cd274, Sox5, Dkk2, Prkcq, Btla*) in UMAP visualizations. Marker genes for subsets are shown in Supplementary Table 13. (**d**) The bar graphs show the change in the percentage of microglia comprising the lymphoid + /*Cd28+* populations (cluster 14) in 5xFAD (1%) and 5xFAD, TIM-3-KO mice (3%).

The importance of CD28 (refs. ^3,4^) and probably other functionally related immunoregulatory receptors in governing microglial responses is underscored by recent findings identifying the T cell immunoglobulin and mucin domain 3 (TIM-3) receptor as a novel regulator of microglial activity in AD^49^. TIM3 deficiency promotes a protective microglia state in 5xFAD mice, characterized by increased phagocytic and reduced pro-inflammatory activity. One of the distinct microglial populations that is expanded upon TIM-3 deficiency expresses CD28 alongside other immune activity regulators, including *Pdcd1* (PD-1), *Cd274* (PDL-1), *Cd5*, *Btla* and *Lat2* while exhibiting markedly reduced PU.1 expression (Extended Data Fig. 12 and Supplementary Table 13). These findings raise the possibility that the neuroprotective effects of TIM-3 deficiency may involve CD28^+^PU.1^low^ microglia, and that the coordinated activity of lymphoid-associated receptor gene expression in plaque-associated microglia underlies neuroprotection in AD.

Our observations support a model in which the switch towards a neuroprotective microglial state depends on the expression of immunoregulatory lymphoid receptor proteins that potentially govern microglial activity in a manner analogous to T cell regulation^3,4,50^. It is tempting to speculate that PU.1^low^ microglia expressing lymphoid gene signatures contribute to neuroprotection not only in mice but also in humans. A gene expression analysis from large cohorts of human microglia^17^ revealed that individuals carrying the PU.1-lowering protective allele (GG/GA versus AA), which was previously associated with improved cognitive performance and reduced AD severity^24^, have an increased abundance of the lymphoid gene-expressing microglia subset (Mic. 13; Fig. 5f,g). The surface expression of CD28 and other lymphoid co-stimulatory or co-inhibitory receptor proteins on neuroprotective PU.1^low^ microglia highlights a new opportunity for antibody-based microglia-driven immunotherapy in AD.

## Methods

### Human samples

Post-mortem human brain tissue samples for immunohistochemistry were obtained from patients who had enrolled in and provided consent for a brain donation programme through the Neuropathology Brain Bank and Research CoRE at the Icahn School of Medicine at Mount Sinai. These samples were collected in accordance with ethical guidelines and institutional review board approval at the Icahn School of Medicine at Mount Sinai, ensuring the privacy and dignity of the donors while supporting ongoing neurological research. Tissue was obtained from donors who had provided written informed consent for research use either directly or through their next of kin. Three post-mortem brain samples (frontal cortex) were obtained from patients affected by Alzheimer’s disease neuropathological changes. Neuropathological assessments were performed at the respective centres using standardized criteria, including the Consortium to Establish a Registry for Alzheimer’s Disease neuritic plaque assessment and Braak neurofibrillary tangle staging^51,52^. In this study, we did not recruit participants; we only de-identified post-mortem brain samples from individuals. Research with de-identified autopsy material does not meet the federal regulatory definition of human subject research, as defined in 45 Code of Federal Regulations Part 46. However, Health Insurance Portability and Accountability Act requirements still apply.

Human samples of induced pluripotent stem cell lines were generated by the Icahn School of Medicine at Mount Sinai, University of California, Irvine Alzheimer’s Disease Research Center (UCI ADRC)and Washington University Alzheimer’s Disease Research Center (WashU ADRC) Induced Pluripotent Stem Cell Core from subject fibroblasts or peripheral blood mononuclear cells (PBMCs) with approved Institutional Review Boards and Human Stem Cell Research Oversight committee protocols at the Icahn School of Medicine at Mount Sinai, UCI ADRC and WashU ADRC Induced Pluripotent Stem Cell Core. The consent for reprogramming human somatic cells to human samples of induced pluripotent stem cell was obtained through the Human Stem Cell Research Oversight protocols 19-04, 2013-9561 and 2017-1061. Informed consent was received from each participant who donated fibroblasts or PBMCs.

Human demographic information, including sex, age, AD diagnosis, Braak state, post-mortem interval and/or clinical cognitive status, can be found in Supplementary Table 2. This study does not report on race, ethnicity or other socially relevant groupings to minimize the likelihood of unintentional identification of de-identified samples.

### Mice

Mice were housed in cages with two to five animals per cage, with a 12-h light/dark cycle (lights on from 0700 to 1900 hours), a constant temperature (23 °C) and ad libitum access to food and water. Humidity averaged 38%, with a high of 58% and a low of 31% over a 24-h period. All animal protocols were approved by the Institutional Animal Care and Use Committee at Icahn School of Medicine at Mount Sinai and performed according to the National Institutes of Health (NIH) guidelines.

The *Spi1*^*fl/+*^ (ref. ^41^), *Cx3cr1*^*CreErt2/+(Litt)*^ (ref. ^53^), *Syk*^*fl/fl*^ (ref. ^54^), *Cd28*^*fl/fl*^ (ref. ^55^) and 5xFAD^25^ (also known as Tg6799) mice were purchased from The Jackson Laboratory (006922, 021160, 017309, 024282 and 034840, respectively). The *Plcg2*^*fl/+*^ mouse^56^ was generously provided by T. Inoue and T. Kurosaki (RIKEN Institute). The *Eef1a1*^*LSL.eGFPL10a/+*^ mouse was generously provided by A. Domingos (Instituto Gulbenkian de Ciência) and J. Friedman (Rockefeller University). The *Mx1::GFP* mouse^57^ was generously provided by A. García-Sastre (Icahn School of Medicine at Mount Sinai).

To achieve ectopic expression of the *Spi1* gene, a floxed transcriptional STOP cassette followed by FLAG-tagged mouse *Spi1* complementary DNA (cDNA) was targeted into the *Rosa R26* locus of embryonic stem cells using the TV-targeting vector for homologous recombination at the ROSA26 locus, generously provided by K. Rajewsky^58^ and available at Addgene (plasmid no. 11739), as previously described. A schematic is shown in Extended Data Fig. 4a. In brief, a transcriptional STOP cassette consisting of six SV40 polyadenylation sites was flanked by *loxP* recognition sequences to facilitate Cre-mediated deletion and subsequent activation of exogenous FLAG-tagged *Spi1*. The original plasmid with mouse *Spi1* sequence was provided by C. Vakoc^24,59^ (Cold Spring Harbor Laboratory). Routine genotyping of *Rosa26*^*LSL.FLAG-Spi1/+*^ mice (Extended Data Fig. 4a) was performed using the following primers:

5′-GTG TTG CAA TAC CTT TCT GGG AGT T

5′-GGA AGT CTT GTC CCT CCA ATT TTA CAC

5′-ACT CCA CAC AGG CAT AGA GTG TCT G

5′-CTGA ATC GTA AGT AAC CAA GTC ATC CGA TG

Wild-type band: 220 bp

Floxed band: 532 bp

To generate SYK-KO mice, *Cx3cr1*^*CreErt2/+(Litt)*^ (ref. ^53^) mice were crossed with *Syk*^*fl/fl*^ (ref. ^54^) mice. To generate PLCγ2-KO mice, *Cx3cr1*^*CreErt2/+(Litt)*^ mice were crossed with *Plcg2*^*fl/fl*^ (ref. ^56^) mice. To generate CD28-KO mice, *Cx3cr1*^*CreErt2/+(Litt)*^ mice were crossed with *Cd28*^*fl/fl*^ (ref. ^55^) mice. To achieve microglia-specific PU.1-low and PU.1-high mice, microglia-specific *Cx3cr1*^*CreErt2/+(Litt)*^ (ref. ^53^) mice were crossed with *Spi1*^*fl/+*^ (ref. ^41^) or *R26*^*FLAG-Spi1/+*^ mice. Microglia-specific ribosomal profiling and single-nucleus sequencing were performed on mice further crossed with *Eef1a1*^*LSL.eGFPL10a/+*^ mice^60^. For ISRE-reporter, *Cx3cr1*^*CreErt2/+(Litt)*^*;Spi1*^*fl/+*^*;*5xFAD mice were additionally crossed with *Mx1::GFP* mice^57^. Unless otherwise specified, *Cx3cr1*^*CreErt2/+(Litt)*^ mice were used as controls. For all experiments involving disease models, mice were crossed with 5xFAD mice^25^. To control for potential differences in expression levels arising from paternal versus maternal transmission of the 5xFAD transgene^61^, litter-matched animals were used for comparison when applicable. Given the reported sex differences in some disease pathology phenotypes^46,47,62^, mice from both sexes were used unless otherwise specified. When both male and female mice were used, an analysis for sex differences was performed; unless otherwise noted, no sex differences were observed. All mice used in the experiments were backcrossed into the C57Bl/6 background for six or more generations.

### Drug treatments of mice

To activate tamoxifen-inducible Cre (CreErt2), all mice were gavaged at 4–6 weeks of age with six doses of 100 mg kg^−1^ of tamoxifen (T5648; Sigma) in corn oil (C8267; Sigma), administered at intervals of at least 48 h. For microglia ablation, 5xFAD mice (4–5 months old) were treated with CSF1R inhibitor PLX5622 (1,200-ppm diet, generously provided by Plexxikon) or vehicle for 4–6 weeks. PLC inhibitor (PLCi) injections were performed intraperitoneally daily for 5 days at 8.7 mg kg^−1^. PLCi U73122 (refs. ^35,63^) was first dissolved in chloroform at 100 mM. This solution was aliquoted and dehydrated using nitrogen. The dehydrated film was dissolved in dimethyl sulfoxide (DMSO) at 5 mM, diluted 1:8 in saline and injected.

### Stereotactic intracerebral Tau injections

AD-Tau was isolated, as previously described, from the brain of a human with AD^64,65^. Using a bicinchoninic acid assay (Thermo Fisher Scientific; 23225), the total protein concentration of the AD-Tau preparation was 21.1 µg µl^−1^. The Tau concentration in the AD-Tau preparation was determined, as previously described, using a Tau-specific sandwich enzyme-linked immunosorbent assay, which was found to be 5.4 µg µl^−1^ (refs. ^64,65^). The AD-Tau preparation was diluted to 0.4 µg µl^−1^ before the injections and sonicated in a water bath sonicator (Qsonica; Q700) for 30 s at 60% amplitude at 4 °C. The 7-month-old 5xFAD (seven females and five males) and 5xFAD;PU.1-low mice (four females and five males) were anaesthetized with isofluorane, immobilized in a stereotactic frame (Kopf Instruments) and unilaterally injected with a total of 2-µg AD-Tau (1 µg per injection site) in the dentate gyrus (Bregma, −2.5 mm; lateral, −2.0 mm; depth, −2.2 mm) and overlying cortex (Bregma, −2.5 mm; lateral, −2.0 mm; depth, −1.0 mm) using a Hamilton syringe (80265–1702RNR; needle, 7803–07). The mice were allowed to recover on a 37 °C heating pad and monitored for the first 72 h after surgery. Although male mice show overall slightly lower levels of amyloid and Tau pathology^62,66^, significant differences between genotypes were preserved as trends in both sexes when the sexes were separated.

### Human induced microglia-like cells

Human iPSCs (see Supplementary Table 2 for demographic information) generated by the Icahn School of Medicine at Mount Sinai, UCI ADRC and WashU ADRC Induced Pluripotent Stem Cell Core from subject fibroblasts or PBMCs were maintained on Matrigel-coated six-well plates (BD Biosciences) in complete StemFlex (Thermo Fisher Scientific; A3349401). iPSCs were passaged every 5–6 days using ReLeSR dissociation reagent (STEMCELL Technologies; 05872) and used for haematopoietic stem cell differentiation with STEMdiff Hematopoietic Kit (STEMCELL Technologies; 05310), followed by differentiation to iMgls using a previously published protocol^67^. The iMgls were maintained and fed with a microglial medium supplemented with three factors (3F medium containing 100 ng ml^−1^ of interleukin (IL)-34, 50 ng ml^−1^ of transforming growth factor (TGF)β and 25 ng ml^−1^ of macrophage colony-stimulating factor) for 25 days. On day 25, cells were collected for nucleofection using P3 Primary Cell 4D-Nucleofector X Kit (Lonza; V4XP-3024) and 4D-Nucleofector System (Lonza; AAf-1003X). The cells were nucleofected with 50 nM of SMARTpool ON-TARGETplus short interfering RNA (siRNA) targeting human *SPI1* (Dharmacon; L-010537-00-0005) or non-targeting pool (Dharmacon; D-001810-10-05). After nucleofection, the cells were transferred into iMgl medium with five factors (5F medium containing 100 ng ml^−1^ of IL-34, 50 ng ml^−1^ of TGFβ, 25 ng ml^−1^ of macrophage colony-stimulating factor, 100 ng ml^−1^ of CXCL1 and 100 ng ml^−1^ of CD200) for an extra 3 days.

### Primary neonatal microglia culture

Microglia were isolated from neonatal (postnatal day P7–P10) mice for ex vivo cell culture and RNA isolation, as previously described^68^. The mice were rapidly killed, brains were extracted and meninges were removed before extracting the forebrain. Tissue was mechanically dissociated in glass homogenizers, and live cells were enriched by centrifugation over 20% Percoll (17-0891-02; GE HealthCare). Microglia were selected using anti-CD11B-coated microbeads (130-093-636; Miltenyi Biotec) with the QuadroMACS separator, following the manufacturer’s recommendations. Cells were manually counted with a haemocytometer using trypan exclusion staining. For immunoblotting, immunocytochemistry and RNA in situ hybridization, the microglia were plated at 250,000 cells per well using DMEM supplemented with 10% fetal bovine serum (FBS; Gibco; 16140) and 100 U ml^−1^ of penicillin–streptomycin (Pen/Strep; Gibco; 15140) and allowed to adhere for 3–4 days before any experimental treatments. For immunocytochemistry and in situ experiments, cells were plated on poly-d-lysine-coated coverslips (8774384; Thermo Fisher Scientific). Initial validation of the protocol for microglial purity was described previously^68^. In each experiment, microglial purity was visually determined by IBA1 or CD11b immunostaining. Cultures were consistently found to be purely microglia. These microglia were used to determine the regulation of PU.1 or CD28 levels following TREM2, CLEC7A or PLC stimulation in Extended Data Figs. 2 and 10a,b.

### Mixed glia culture and primary neonatal microglia isolation

Primary microglia were isolated from postnatal days 2–3 CD1 mouse pups, as described previously^69^. In brief, brains were collected in Leibovitz's L-15 medium, and cortices were dissected. Cortical tissue was lysed at 37 °C for 25 min using 2.5% trypsin (Thermo Fisher Scientific; 15090046). Afterward, DNase I (Sigma; D4263-1VL) was added, and cells were pelleted at 270*g* for 5 min at 4 °C. The pellet was resuspended in DMEM (Thermo Fisher Scientific; 11965092) supplemented with 10% FBS (Gibco; 10437) and 1% Pen/Strep/l-glutamine (Gibco; 10378016) and passed through a 70-μm cell strainer. The cells were seeded into poly-d-lysine-coated T75 flasks and incubated at 37 °C with 5% CO_2_. The medium was changed on day in vitro (DIV)2 and supplemented with L929-conditioned medium on DIV6 and DIV10. On DIV11–14, microglia were collected and seeded onto poly-d-lysine-coated plates using TGF-β, IL-34, and Cholesterol medium (DMEM/F12 phenol red-free (Gibco; 21041), 1% Pen/Strep/l-glutamine, 5 µg ml^−1^ of *N*-acetyl cysteine (Sigma; A9165), 5 µg ml^−1^ of insulin, 5 µg ml^−1^ of apo-transferrin, 5 ng ml^−1^ of sodium selenite (Roche; 11074547001), 2 ng ml^−1^ of TGFß2 (PeproTech; 100-35B), 10 ng ml^−1^ of colony-stimulating factor 1 (PeproTech; 315-02), 1.5 µg ml^−1^ of cholesterol (Sigma/Avanti; 700000 P) and 1% FBS. These microglia were used for PU.1 or CD28 quantification following siRNA magnetofection, as shown in Extended Data Fig. 5d.

### siRNA magnetofection of primary mouse microglia

Primary microglia were magnetofected 24 h after seeding using the Glial-Mag magnetofection kit (GL00250), following the manufacturer’s instructions. In brief, siRNA (non-targeting control pool (scramble), *Spi1* and *Cd28*; Horizon Discovery; D-001810-10-05, L-041420-00-0005 and L-058630-00-0005, respectively) was diluted in DMEM (Gibco; 11-965-092) and mixed with Glial-Mag transfection reagent. After a 20-min incubation at room temperature, the solution was added to each respective well in a dropwise manner. The Glia-Boost reagent was added directly to cells to a final concentration of 1×. The final concentration of siRNA was 25 nM. Cells were then incubated on top of a magnetic plate (OZ Biosciences; MF10000) inside a humidified incubator at 37 °C and 5% CO_2_ for 30 min. The magnetic plate was removed, and cells were incubated for an extra 3 h. Afterward, the medium was removed and replaced with 50% collected conditioned medium + 50% fresh TGFβ–IL34–cholesterol medium. Knockdown efficiency was analysed 48 h after magnetofection using quantitative polymerase chain reaction (qPCR) and western blot.

### Immortalized microglia BV2 cell line

The BV2 mouse microglial cell line was provided by M. Diamond (UT Southwestern Medical Center). It has been maintained by the Goate Laboratory for the past 15 years and by the Schaefer Laboratory for the past 10 years, with long-term storage in liquid nitrogen. BV2 cells have been authenticated on the basis of their characteristic microglia-like morphology, positive immunolabelling for microglia markers (CD11B, IBA1 and PU.1) and gene and protein expression analysis using RNA-seq and proteomic analysis. *Mycoplasma* contamination testing was performed on a regular basis every 3 months, and all cell lines tested negative. The BV2 cells were cultured in DMEM (Gibco; 11965) supplemented with 5% heat-inactivated FBS and 100 U ml^−1^ of Pen/Strep. We have previously generated BV2 cells stably overexpressing mouse PU.1 and respective empty frame control, as well as BV2 cells with knockdown of PU.1 with the respective scrambled control^39^. Cells were used for western blot measurement of PU.1 and CD28 (Extended Data Fig. 5b), mass spectrometry analysis (Extended Data Fig. 5c) and signalling phospho-protein analysis (Extended Data Fig. 6b).

### Drug treatments of primary mouse microglia

For TREM2 activation, PS/PC liposomes (70% DOPC and 30% POPS) resuspended in phosphate-buffered saline (PBS) were purchased from CD Bioparticles (CDECPS-1676). Microglial cultures were treated with liposomes resuspended in PBS at 0.1 mg ml^−1^ and 1 mg ml^−1^ for 12 h and 3 days, unless otherwise stated. For washout experiments, cells were treated with PS/PC liposome or vehicle for 3 days, the liposome-containing medium was replaced with fresh medium and cells were analysed 4 days later. Liposome-treated cells were imaged at the end of the 3-day treatment. For PLCi and PLC activator (PLCa) experiments, we used 0.1 μM U73122 (PLCi; Tocris) and 40 μM m-3M3FBS (PLCa; Tocris). U73122 was first dissolved in chloroform at 100 mM. This solution was aliquoted and dehydrated using nitrogen. The dehydrated film was dissolved in DMSO at 5 mM. m-3M3FBS was prepared using DMSO. The final DMSO concentration in the medium was 0.2%. PLCi was added 1 h before liposome treatment. For CLEC7A activation, pustulan (InvivoGen; tlrl-pst) was resuspended in PBS. Microglial cultures were treated with 50 μg ml^−1^ of pustulan for 3 days.

### Chromogenic multiplex immunohistochemistry of human brain tissue

The 5-μm-thick paraffin-embedded post-mortem brain sections from individuals with AD (see Supplementary Table 2 for demographic information) were pretreated using Cell Conditioning CC1 antigen retrieval buffer (Tris/borate/EDTA buffer; pH 8.0–8.5; 950-224; Roche Diagnostics) and incubated in rounds of staining with primary antibodies against PU.1 (clone 9G7; Cell Signaling Technology; 2258S; 1:50), IBA1 (Thermo Fisher Scientific; PA5-27436; 1:500) to label microglia, β-amyloid (clone 4G8; BioLegend; 800701; 1:4,000) and secondary antibody Multimer HRP OmniMap Anti-Mouse and Multimer HRP OmniMAP Anti-Rabbit (760-4310 and 760-4311; Roche Diagnostics; 1:1). Detection was performed using the DISCOVERY Purple HRP Kit, DISCOVERY Green HRP Kit and DISCOVERY Yellow HRP Kit (760-229, 760-271 and 760-250, respectively; Roche Diagnostics). Sodium citrate antigen retrieval (pH 6.0) was used between rounds to remove the antibody from the previous round and avoid cross-reactivity. Haematoxylin and bluing reagent (760-2021 and 760-2037; Roche Diagnostics) were used as a nuclear counterstain.

### Image analysis of immunohistochemistry of human brain tissue

Digital images of the stained sections were captured at ×40 using the Aperio GT 450 high-resolution scanner (Leica Biosystems). Images were also acquired using the Nikon Eclipse Ci microscope at ×20 and ×40 magnification. Images were analysed using QuPath (v.0.5.1). For each sample, three regions of interest (ROI) were annotated in plaque-dense areas, and three ROI were annotated in plaque-depleted areas in the cortical grey matter, with each ROI measuring 1 mm^2^. Microglia were manually counted in each ROI. Microglia were considered plaque-associated if there was at least one IBA1^+^ process co-localized with an amyloid-β plaque. If microglia did not meet these criteria, the cell was considered distal. PU.1 level was manually scored as either low or high on the basis of the staining intensity of PU.1 within microglia nuclei.

### Immunocytochemistry of primary microglia

For PU.1 and CD28 quantification, primary microglia isolated by anti-CD11B antibody were washed twice with PBS and fixed with 4% paraformaldehyde (PFA) at room temperature for 15 min, followed by three more PBS washes. Coverslips were washed and permeabilized with 0.2% PBS–Tween, followed by a 1-h blocking step with 4% normal goat serum in 0.2% PBS–Tween. The coverslips were then incubated with the following primary antibodies overnight at 4 °C: PU.1 (1:200 Cell Signaling Technology; 2258), CD11B (1:500; Thermo Fisher Scientific; 14-0112-85) and IBA1 (1:500; Synaptic Systems; 234 009) to label microglia and/or CD28 (1:50; Cell Signaling Technology; 38774).The coverslips were washed three times with PBS + 0.2% Triton X-100 (PBST) and incubated for 1 h at room temperature with secondary antibodies (goat anti-rabbit 1:500 and goat anti-rat 1:500), conjugated to Alexa Fluor 488, Alexa Fluor 546 or Alexa Fluor 568. To label nuclei, the coverslips were washed twice in PBST and once in PBS and then stained with DAPI (1:10,000 in PBS) for 20 min at room temperature.The coverslips were then embedded on a microscopy slide using ProLong Gold Antifade Mountant with DAPI (Invitrogen) and dried overnight. Imaging was performed using an LSM 780 confocal microscope (ZEISS). For *z*-stack images, 20-μm *z*-stack confocal images were acquired at 2-μm intervals, with ×20 objective at ×1 zoom. Image processing was performed using ZEN 2011 software (ZEISS). Image quantification of CD28 in vitro, *z*-section confocal microscopy images were applied to custom ImageJ macros. Using the maximum intensity projected *z* stack of the CD28 channel, the CD28 signal was detected using the default ImageJ threshold and measured using integrated density (as shown in figure quantifications unless otherwise specified) and mean grey value, both of which yielded comparable results.

### Combined RNA in situ hybridization and immunocytochemistry of primary microglia

Cells were washed twice with PBS and fixed with 4% PFA at room temperature for 15 min, followed by three more PBS washes. Coverslips were then dehydrated in 50%, 70% and 100% ethanol and allowed to dry before in situ hybridization. In situ hybridization was performed using RNAscope custom-designed probes for *Spi1* in combination with the RNAScope 2.0 RED Kit, according to the manufacturer’s recommendation (Advanced Cell Diagnostics). After completing in situ hybridization, sections were rinsed with double-distilled water and a gradient concentration of PBS (0.1×–1×) before the colorimetric reaction, blocked in 2% normal goat serum in PBS for 1 h at room temperature. The coverslips were then incubated with primary antibodies overnight at 4 °C: PU.1 (1:200; Cell Signaling Technology; 2258) and CD11B (1:500; Thermo Fisher Scientific; 14-0112-85) to label microglia. The coverslips were then washed three times with PBST and incubated with a secondary antibody (goat anti-rabbit 1:500; goat anti-rat 1:500, conjugated to the fluorophore Alexa Fluor 488 or Alexa Fluor 546) for 1 h at room temperature. To label nuclei, the coverslips were washed twice in PBST and once in PBS and then stained with DAPI (1:10,000 in PBS) for 20 min at room temperature. Sections were rinsed with a gradient concentration of PBS (0.1×–1×) and ddH_2_O, subjected to colorimetric reaction, dried for 15 min at 60 °C, mounted using EcoMount (EM897L; Biocare Medical) and dried overnight. *Z*-stack images were taken on a ZEISS LSM 780 confocal microscope with 20-μm *z*-stack confocal images acquired at 2-μm intervals and ×20 objective at ×1 zoom. Image processing was done using ZEN 2011 software.

### Image analysis of RNA in situ hybridization and immunocytochemistry of primary microglia

*Z*-section confocal microscopy images were applied to custom ImageJ macros. Nuclei in the DAPI channel were segmented using the default ImageJ threshold, followed by the ‘AnalyzeParticles’ function without size and circularity constraints to identify all microglia nuclei. These DAPI ROI were then projected onto PU.1 maximum intensity projected *z* stack of the corresponding PU.1 channel to measure the nuclear PU.1 signal through mean grey value (as shown in figure quantifications unless otherwise specified) and integrated density measurements, both of which yielded comparable results in PU.1 signal in treatment versus control conditions. For the quantification of microglia *Spi1* signal from in situ hybridization and correlation analyses between PU.1 versus *Spi1* signal within microglia, the ImageJ freehand selection tool was used to manually outline the borders of individual microglia (ROI for *Spi1*) along with their corresponding nuclei (ROI for PU.1). Within these microglia, the ROI mean grey value and integrated density measurements were calculated, yielding comparable results in *Spi1* and PU.1 signal in treatment versus control. At least 30 microglia from each coverslip from each condition were quantified this way, and the average mean grey value of these cells is shown in the figure quantifications unless otherwise specified.

### Preparation of mouse tissue for imaging

For immunofluorescence and RNA in situ hybridization of mouse tissue, mice were anaesthetized with ketamine (120 mg kg^−1^) and xylazine (24 mg kg^−1^) and perfused transcardially with 10-ml PBS and 40 ml of 4% PFA (Electron Microscopy Sciences), as previously described^70^. Fixed brains were removed and dehydrated in 15% and 30% sucrose in PBS. Following dehydration, the brains were frozen in Neg-50 (Life Technologies) on dry ice and stored at −80 °C until further processing. The brains were cut using a cryostat, and 10-μm or 30-μm sections were mounted on Superfrost Plus Microscope Slides (Thermo Fisher Scientific), which were stored at −80 °C until staining.

For immunohistochemistry of AT8 and amyloid-β, 10-month-old 5xFAD (seven females and five males) and 5xFAD;PU.1-low (four females and five males) mice were killed through intraperitoneal injection of pentobarbital (200 mg kg^−1^), 3 months after the AD-Tau injection. Whole brains were extracted and fixed in 4% PFA for 24 h before being transferred to 30% sucrose and stored at 4 °C until sectioned. The brains were cut coronally into 30-μm sections on a freezing sliding microtome (Microm; HM 400) and stored in a cryoprotectant solution (0.2 M PBS, 15% sucrose and 33% ethylene glycol) at −20 °C until use. A notch on the left hemisphere at the piriform cortex ensured proper identification of the ipsilateral AD-Tau-injected side.

### Immunohistochemistry of mouse tissue

For immunohistochemical staining of AD-Tau^64,65^ and amyloid-β, sections were washed three times in Tris-buffered saline (TBS) for 5 min and incubated in 0.3% hydrogen peroxide for 10 min. After washing, sections were blocked for 30 min in 3% milk in TBS containing 0.25% Triton X-100. The primary antibody was diluted in 1% milk in TBS containing 0.25% Triton X-100, and sections were incubated in the primary antibody overnight at 4 °C (AD-Tau (AT8; mouse monoclonal; 1:500; Thermo Fisher Scientific; MN1020B) and amyloid-β (HJ3.4 biotinylated; anti-amyloid-β N terminus 1–16; mouse monoclonal; 2 µg ml^−1^ generated in-house^71^). The next day, sections were washed three times and then incubated in ABC Elite solution (Vectastain; PK-6100) for 1 h, prepared following the manufacturer’s instructions, followed by another washing step. Sections were developed in DAB solution (Sigma; D5905 for AT8; anti-amyloid-β N terminus 1–16 antibody; clone HJ3.4), washed and mounted on slides. After drying overnight, the slides were dehydrated in increasing ethanol concentrations followed by xylene and coverslipped with CytoSeal 60 (Thermo Fisher Scientific; 8310).

### Immunofluorescence of mouse tissue

For microglia size and number quantification, plaque-associated microglia quantification, 6E10, ThioS and boron dipyrromethene (BODIPY) imaging, 30-μm slides from 5xFAD;PU.1-low, 5xFAD–SYK-KO, 5xFAD;PLCγ2-KO or 5xFAD;CD28-KO mice with sex-matched and age-matched littermate control 5xFAD mice were washed with PBS, permeabilized with PBST and blocked with 2% normal goat serum in PBST. Slides were incubated with primary antibodies in 2% normal goat serum in PBST overnight at 4 °C. The primary antibodies included CD11B (1:1,000; MCA711GT; Bio-Rad) and IBA1 (1:750; Synaptic Systems; 234 009) to label microglia and 6E10 (1:500; 803004; BioLegend) to label amyloid-β. The slides were then washed and incubated with Alexa Fluor-conjugated secondary antibody (Alexa Fluor 488-labelled, 546-labelled or 568-labelled goat anti-rat IgGs heavy and light chains (H + L); 1:500; Life Technologies) in 2% normal goat serum in PBST for 1 h at room temperature. The slides were washed and incubated with Thioflavin S for 2 min (1% Thioflavin S diluted 1:1,000 in PBS; Sigma-Aldrich; T1892) to label dense-core plaques, followed by DRAQ5 for 20 min (1:2,500 in PBS) to label nuclei. For neutral lipid droplet imaging, BODIPY 505/515 (4,4-difluoro-1,3,5,7-tetramethyl-4-bora-3a,4a-diaza-*s*-indacene; Thermo Fisher Scientific; D3921) was used to label lipid droplets, according to the manufacturer’s recommendations. Imaging was performed using a ZEISS LSM 780 confocal microscope. Confocal *z*-stack images (20 μm) were acquired at 2-μm intervals using a ×40/1.3 oil objective. Image processing was performed using ZEN 2011 software (ZEISS). Compressed *z*-stack immunofluorescence images were used as representative images unless otherwise specified.

For PU.1 quantification, 30-μm slides from 6-month-old sex-matched control, 5xFAD, CSF1Ri-treated 5xFAD, 5xFAD–SYK-KO, 5xFAD;PLCγ2-KO, SYK-KO, PLCγ2-KO, PU.1-low and/or PU.1-high and 20-month-old control mice were washed with PBS. Antigen retrieval was performed using 0.1% Triton X-100 and 0.1% sodium citrate for 10 min at room temperature, followed by DNAse treatment of the fixed tissue to increase nuclear accessibility to primary antibodies. The slides were blocked with 2% normal goat serum in PBST and then incubated overnight at 4 °C with primary antibodies in the same blocking solution. The primary antibodies included PU.1 (1:200; Cell Signaling Technology; 2258) and CD11B (1:500 Thermo Fisher Scientific; 14-0112-85) or IBA1 (1:750; Synaptic Systems; 234 009) to label microglia. The slides were then washed and incubated with Alexa Fluor-conjugated secondary antibodies (Alexa Fluor 488-labelled, 546-labelled and 568-labelled goat anti-mouse, goat anti-rat, goat anti-chicken, goat anti-rabbit or donkey anti-goat IgGs (H + L); 1:500; Life Technologies) in 2% normal goat serum in PBST for 1 h at room temperature. To label nuclei, the slides were incubated with DRAQ5 (1:2,000 in PBS; Life Technologies; 62254), washed, coverslipped using ProLong Gold Antifade (Invitrogen) and dried overnight. For images containing plaques, the plaques were stained with Thioflavin S for 2 min after secondary antibody incubation (1% Thioflavin S diluted 1:5,000 in PBS; Sigma-Aldrich; T1892) to label dense-core plaques. Nuclei were labelled using DRAQ5 (1:2,000 in PBS) for 20 min. Imaging was performed using a ZEISS LSM 780 confocal microscope. Confocal *z*-stack images (20 μm) were acquired at 2-μm intervals using a ×40/1.3 oil objective. Image processing was performed using ZEN 2011 software (ZEISS). Compressed *z*-stack immunofluorescence images were used as representative images unless otherwise specified.

For CD28 quantification, 30-μm slides from 5xFAD;CD28-KO and 5xFAD;PU.1-low mice with sex-matched and age-matched littermate control 5xFAD mice were washed with PBS, permeabilized with PBST and blocked with 2% normal goat serum in PBST. The slides were incubated with primary antibodies in 2% normal goat serum in PBST overnight at 4 °C. The primary antibodies included IBA1 (1:500; Synaptic Systems; 234 009) to label microglia and CD28 (1:500; Cell Signaling Technology; 38774). The slides were then washed with PBST and incubated with Alexa Fluor-conjugated secondary antibodies for 1 h (Alexa Fluor 488-labelled goat anti-chicken IgG (H + L); 1:500; Life Technologies). Tyramide amplification for CD28 using 568 tyramide conjugate was performed following the manufacturer’s instructions (Tyramide SuperBoost Kit goat anti-rabbit IgG (Thermo Fisher Scientific; B40922) and Tyramide Conjugate (Invitrogen; B40956)). The slides were washed and incubated with DRAQ5 (1:2,000 in PBS) for 20 min to label nuclei, followed by Thioflavin S staining (1% Thioflavin S diluted 1:5,000 in PBS; Sigma-Aldrich; T1892) for 2 min to label dense-core plaques. Imaging was performed using a ZEISS LSM 780 confocal microscope. Confocal *z*-stack images (20 μm) were acquired at 2-μm intervals using a ×40/1.3 oil objective. Image processing was performed using ZEN 2011 software (ZEISS). Compressed z-stack immunofluorescence images were used as representative images unless otherwise specified.

For VGluT2, Bassoon and C1Q quantification, 10-µm coronal brain sections from control, 5xFAD and 5xFAD;PU.1-low mice were blocked and permeabilized for 1 h at room temperature in 10% normal goat serum/0.1 M phosphate buffer containing 0.3% Triton X-100 (all from Sigma). Sections were then incubated with primary antibodies at room temperature overnight. The following primary monoclonal and polyclonal antibodies have been used: mouse monoclonal anti-Bassoon (Enzo Life Sciences; ADI-VAM-PS003-F; 1:500) and guinea pig polyclonal anti-VGLUT2 (Synaptic Systems; 135404; 1:2,000) to label presynaptic axonal terminals and C1Q (Abcam; ab182451; 1:500). The following day, sections were incubated with appropriate Alexa-fluorophore-conjugated secondary antibodies (Thermo Fisher Scientific), and β-amyloid plaques were visualized using 0.0005% Thioflavin S in 1× PBS (Sigma-Aldrich; T1892) to label dense-core plaques. After thorough washing, sections were mounted using VECTASHIELD without DAPI (Vector Laboratories).

For *Mx1*–GFP^+^ microglia quantification, 30-μm slides from 5xFAD;*Mx1*–GFP (two females and two males) and 5xFAD;*Mx1*–GFP;PU.1-low mice (two females and one male) were washed with PBS, permeabilized with PBST and blocked with 2% normal goat serum in PBST. The slides were incubated with primary antibodies in 2% normal goat serum in PBST overnight at 4 °C. The primary antibodies included CD11B (1:500; eBioscience; 14-0112-82) to label microglia and turboGFP (1:5,000; Evrogen; AB513) to label Mx1–GFP^+^ interferon-responsive cells. The slides were then washed, and the signal for turboGFP was amplified for 10 min using tyramide signal amplification with SuperBoost (Invitrogen; B40922). The slides were washed and incubated with Thioflavin S for 2 min (1% Thioflavin S diluted 1:10,000 in PBS; Sigma-Aldrich; T1892) to label dense-core plaques and then with DRAQ5 for 20 min (1:2,000 in PBS) to label nuclei. For BODIPY imaging, the manufacturer’s recommendations were followed. Imaging was performed using a ZEISS LSM 780 confocal microscope. Confocal *z*-stack images (20 μm) were acquired at 2-μm intervals using a ×40/1.3 oil objective. Image processing was performed using ZEN 2011 software (ZEISS). Compressed *z*-stack immunofluorescence images were used as representative images unless otherwise specified.

### Imaging quantification

For most image analyses (with the exception of the analyses indicated below), single eight-tile *z*-stack images with a ×20 objective (×1 zoom) were analysed using Imaris software (Bitplane). First, a surface for plaques was generated. Next, microglia surfaces (processes) within 5 μm of the plaque surface were generated to select for plaque-associated microglia. We used this cutoff to select for microglia virtually touching the plaques. Finally, surfaces were generated for plaque-associated microglial nuclei. The number of nuclei per plaque, the surface area of plaques and the volume of plaques were calculated. The distal microglia were counted similarly for those more than 5 μm from the plaque surface. They were normalized to cortex volume minus total plaque volume. The total microglia numbers were calculated similarly. For volume calculations, the volume of the microglia surface was divided by the number of nuclei. For BODIPY quantification, a microglia surface was generated, and then a BODIPY^+^ surface was generated within microglia. The volume of the BODIPY surface per microglia volume was calculated. *Mx1::GFP*^*+*^ microglia were quantified manually.

For Bassoon and VGLUT2 density quantification, three to four stained sections from each sample containing the somatosensory cortex were imaged using a ZEISS Observer Spinning Disk Confocal microscope equipped with diode lasers (405 nm, 488 nm, 594 nm and 647 nm) and ZEN Blue acquisition software (ZEISS). Two to three randomly chosen ×63 fields of view within the somatosensory cortex were acquired with five *z*-stack steps at 0.68 µm spacing for each hemisphere. Identical settings were used to obtain images from all samples within one experiment, and data analyses were performed using ImageJ (National Institutes of Health, v.1.53k), as described previously, with some modifications^72^. In brief, a consistent threshold range was first determined using sample images for each genotype and condition. All images were subjected to background subtraction. Then, single *z*-planes of the *z*-stacks (five *z*-planes per animal) were subjected to the same background subtraction and thresholding. Finally, the total area of presynaptic inputs was measured from the thresholded images using the AnalyzeParticles function of ImageJ. Data from single *z*-planes were averaged for each *z*-stack of each field of view, and the mean of all fields of view from one animal was determined and normalized to control animals to assess changes in synaptic densities. For inhibitor experiments, manual thresholding, blinded to condition and genotype for each channel within one experiment, was performed (ISODATA segmentation method; 85–255).

For AT8 and HJ3.4–amyloid-β immunohistochemistry quantification, images were obtained from two to three sections per mouse. Slides were scanned on the NanoZoomer 2.0-HT system (Hamamatsu Photonics). The images were further processed using NDP viewing software (Hamamatsu Photonics) and Fiji software v.1.51 (National Institutes of Health).

For 6E10–amyloid-β/ThioS ratio quantification from the hippocampus and subiculum, *z*-stack images of approximately 20-μm thickness acquired with a ×40 objective at ×1 zoom were used to quantify the area of the respective signals in maximum intensity *Z* projections in ImageJ.

For PU.1 immunofluorescence quantification by CellProfiler (Figs. 1e and 3a), approximately 20 μm of mouse brain tissue stained for PU.1, CD11B, DRAQ5 and ThioS was captured by *z*-section confocal microscopy. The *z* sections were projected as summed slices and converted to 16-bit. Images stained for CD11B (microglia marker) were normalized to 0.35% pixel saturation to standardize microglia segmentation (outlining) in Fiji/ImageJ^73^. The *z*-projected images were then loaded into CellProfiler v.4.2.1 (ref. ^74^), in which microglia nuclei were identified, the nuclear outline was applied to the PU.1 channel and measurements of individual nuclei were recorded. CellProfiler classified nuclei as belonging to microglia using FilterObjects if they exceeded an upper quartile intensity threshold in the CD11B stain and passed spatial form factor and eccentricity thresholds. To further constrain microglia nuclei, CellProfiler was used to generate a properties file containing DRAQ5 nuclei objects and the associated CD11B and DRAQ5 measurements. The classifier feature in CellProfiler Analyst v.3.0.4 (ref. ^75^) was then trained using the properties file by visually selecting representative microglia and non-microglia nuclei, applying the training and saving a .model file. A separate CellProfiler pipeline was then created to incorporate this classifier model under ClassifyObjects. Nuclear outlines were reduced in size for consistency. Microglial PU.1 values, merged images containing outlines and mean intensity measurements to allow visual inspection were recorded.

For PU.1 immunofluorescence quantification by Imaris (Fig. 1b,f and Extended Data Fig. 1a,e), approximately 20 μm of mouse brain tissue from control, 5xFAD, 5xFAD;SYK-KO and 5xFAD–PLCγ2-KO, as well as PU.1-low and PU.1-high mice (as reference mice), stained for PU.1, IBA1, DRAQ5 and Thioflavin S, was captured using *z*-section confocal microscopy. The *z* sections were analysed using Imaris software (Bitplane). First, a surface for IBA1^+^ microglia was generated, and the DRAQ5 nuclei channel was masked within this surface. Next, microglia nuclei were specifically detected by creating a surface for the masked DRAQ5 channel. The mean signal intensity for the PU.1 channel was measured within the microglia nuclei DRAQ5 surface. Low and high PU.1 cutoffs were defined on the basis of the fold change in mean PU.1 signal between nuclei from PU.1-high and PU.1-low microglia. The fold change (calculated as the mean PU.1 signal in PU.1-high nuclei divided by the mean PU.1 signal in PU.1-low nuclei) was determined to be 1.25. For each experiment, the high cutoff was set by multiplying and the low cutoff was set by dividing the mean PU.1 signal across all microglia of 6-month-old 5xFAD mice. Nuclei between these cutoffs were considered to have medium PU.1 signal. Quantifications represent the proportion of microglia within each of these PU.1 intensity categories normalized to the total number of microglia analysed.

For CD28 quantification, approximately 20 μm of mouse brain tissue stained for CD28, IBA1, DRAQ5 and ThioS was captured by *z*-section confocal microscopy. The *z* sections were analysed using Imaris software (Bitplane). Microglia nuclei in each image were detected and counted using the Spots function. IBA1^+^CD28^+^ microglia were counted and normalized to the total number of microglia per image.

### Combined RNA in situ hybridization and immunofluorescence of mouse tissue

In situ hybridization was performed on 30-μm cryosectioned slides from 5xFAD;CD28-KO mice with sex-matched and age-matched littermate control 5xFAD mice using RNAscope probes for *Cd28* (Advanced Cell Diagnostics; 1070671-C1) and *Mx1* (Advanced Cell Diagnostics; 474931-C1) to label interferon-responsive cells, with the RNAscope Multiplex Fluorescent Reagent Kit v.2 according to the manufacturer’s recommendation for fixed-frozen tissue samples. After completing the in situ hybridization, sections were rinsed in PBST for 15 min and blocked in 2% normal goat serum in PBS for 1 h at room temperature. Sections were incubated overnight at 4 °C with the primary antibody IBA1 (1:1,000; Synaptic Systems; 234 009) to label microglia. Sections were washed three times with PBST for 10 min and incubated with a secondary antibody (goat anti-chicken; 1:500; conjugated to the fluorophore Alexa Fluor 488) and DAPI (1:10,000) to label nuclei for 1 h at room temperature. Sections were washed twice in PBST and once in PBS, dried, mounted using ProLong Gold Antifade Mountant (P36930; Invitrogen) and dried overnight. *Z*-stack images were taken using a ZEISS LSM 780 confocal microscope with 30-μm *z*-stack confocal images acquired at 0.8-μm intervals and ×20 objective at ×1 zoom, and image processing was done using Fiji software. Microglia were identified by IBA1. *Cd28*^*+*^ or *Mx1*^*+*^ microglia were manually identified by at least five dots (*Cd28*) or three dots (*Mx1*) of in situ signal within the microglia nuclei.

### Multiplexed error-robust fluorescence in situ hybridization

For MERFISH, we used two control (*Cx3cr1*^*CreErt2/+(Litt)*^), three 5xFAD (*Cx3cr1*^*CreErt2/+(Litt)*^*;*5xFAD), two 5xFAD;PU.1-low (5xFAD;*Cx3cr1*^*CreErt2/+(Litt)*^*;Spi1*^*fl/+*^) and one 5xFAD;PU.1-high (5xFAD;*Cx3cr1*^*CreErt2/+(Litt)*^*;R26*^*FLAG-Spi1/+*^) male mice (8–10 months old). After sacrificing the mice, the brains were immediately placed in a cold O.C.T. Compound (Fisher; 23-730-571) and frozen. The samples were then processed following the Vizgen Fresh Frozen Tissue Sample Preparation protocol. Briefly, brains were sagittally sectioned into 10-µm-thick slices and placed on a functionalized coverslip covered with fluorescent beads (Vizgen; 20400001). Once the tissue adhered to the coverslip, we fixed the tissue (4% PFA in 1× PBS) for 15 min at room temperature, followed by three washes with PBS. After aspiration, we added 70% ethanol to permeabilize the tissue for 24 h at 4 °C. The samples were photobleached for 3 h in 70% ethanol (MERSCOPE Photobleacher; Vizgen; 10100003), followed by incubation with a blocking solution (Vizgen Blocking Buffer C premix; 20300100; and 10% RNase inhibitor; New England Biolabs; M0314L) for 1 h at room temperature. Then, the samples were incubated with a primary antibody solution (purified azide-free anti-β-amyloid 6E10; 803004; BioLegend; and 1:100 plus 10:100 of RNase inhibitor; New England Biolabs; M0314L). The samples were washed three times with 1× PBS and incubated with the secondary antibody solution (Vizgen; Anti-Mouse Aux 4 20300101; and 1:100 plus 10:100 of RNase inhibitor; New England Biolabs; M0314L) for 1 h at room temperature. The samples were washed three times with 1× PBS, fixed (4% PFA in 1× PBS) for 15 min at room temperature and washed two times with 1× PBS. Following staining, the samples were incubated in formamide wash buffer (30% formamide in 2× saline–sodium citrate (SSC) buffer), and the MERFISH library mix was added and left for hybridization for 48 h. We then washed the samples twice and incubated them at 47 °C with formamide wash buffer. Then, the tissue was embedded in a polyacrylamide gel, followed by incubation overnight at 37 °C using a tissue clearing solution (2× SSC, 2% SDS, 0.5% v/v Triton X-100 and 1:100 proteinase K). The tissue was washed (2× SSC) and incubated with DAPI and PolyT solution (Vizgen) for 15 min at room temperature. The coverslip was assembled into the imaging chamber and placed into the microscope for imaging. The MERSCOPE 500 gene imaging kit (Vizgen; 10400003) was activated by adding 250 μl of Imaging Buffer Activator (Vizgen; 203000015) and 100 μl of RNase Inhibitor (New England Biolabs; M0314L). Mineral oil (15 ml) was overlaid on top of the imaging buffer through the activation port. The instrument was primed, and the imaging chamber was assembled according to the MERSCOPE user guide. A ×10 low-resolution mosaic of the sample was acquired. The imaging area was then selected, and the sample was imaged. The settings for the MERSCOPE antibody image acquisition were set to high.

Following image acquisition, the resulting data were decoded using Vizgen’s analysis pipeline incorporated in MERSCOPE. Vizgen’s post-processing tool was then applied to obtain the cell segmentation on the basis of the DAPI staining using the Cellpose algorithm. Segmentation was performed on the middle *z* plane (fourth of seven), and cell borders were propagated to *z* planes above and below. The following gene sets have been used to identify cell types in the representative images: macrophage-identifying gene (*Mrc1*), endothelial cell-identifying gene (*Cldn5*), T cell-identifying genes (*Cd3e* and *Cd8a*), microglia homeostatic genes (*Cx3cr1*,* Tmem119*, *P2ry12*, *Csf1r*, *Hexb*, *Aif1*, *Tnf*, *Trem2*, *Itgam*, *C1qa*, *C1qb*, *C1qc*, *Sall1* and *Selplg*), neuron-identifying gene (*Meg3*), oligodendrocyte-identifying genes (*Mog*, *Sox10* and *Mag*), lymphoid genes (*Pdcd1*, *Cd274*, *Cd28*, *Cd72*, *Tnfsf13b* and *Cd4*) and DAM-identifying genes (*Itgax*, *Clec7a*, *Igf1*, *Cst7* and *Spp1*) (Extended Data Fig. 3g).

### Bioinformatics analysis of MERFISH data

Single-cell gene expression matrices were obtained by counting messenger RNA (mRNA) molecules within segmented cell boundaries and were further analysed in RStudio using R 4.2.2, Seurat 5.0.3 and custom-made scripts (https://github.com/SchaferLabUMassChan/Ayata-et-al_2025). We excluded cells containing fewer than 40 transcripts or a volume of less than 100 µm^3^. To account for global differences in mRNA counts between samples, we normalized data to the total transcripts per cell of each sample. We then normalized the gene expression of each cell by its volume to compute counts per cubic micrometre. Cells with less than 11 unique genes were further excluded.

For annotation of cell types and nearest-neighbour analysis relative to plaques, data from all samples were merged into a single Seurat object for clustering and cell type annotation following a modified Seurat pipeline. Data were normalized by dividing gene counts per cubic micrometre for each cell by the total count per cubic micrometre for that cell, multiplied by 10,000 and log-transformed. Data were then scaled, and the principal components were calculated on all 398 measured genes. Thirty-nine principal components were used to calculate the UMAP embedding and perform clustering analysis using the Louvain algorithm with a resolution of 2.4. Clusters were manually annotated on the basis of the spatial distribution of the cells in the tissue and expression of cell-type-specific marker genes. Owing to imperfections in cell boundary segmentation, a small fraction of cells expressed cell type markers for several cell types. Clusters composed of these ‘hybrid’ cells and clusters consisting of imaging artefacts were removed from the analysis. UMAP embedding and clustering analysis were iteratively repeated until all hybrid clusters were removed.

Microglia sub-cluster analysis was performed using the same modified Seurat pipeline. Before the removal of clusters containing hybrids, all clusters expressing microglia cell type markers (*Tmem119*, *Hexb*, *P2ry12*, *Trem2*, *C1qa* and *Cx3cr1*) were separated out and sub-clustered. Sub-clusters composed of hybrid cells and sub-clusters composed of border-associated macrophages (*Mrc1*, *Lyve1* and *Cd163*) were removed from the analysis. Seventeen principal components were used to calculate the UMAP embedding and perform clustering analysis using the Louvain algorithm with a resolution of 0.7.

The *X* and *Y* coordinates of all amyloid-β plaques in the cortex of two 5xFAD MERFISH samples were manually recorded using MERSCOPE visualizer software (Vizgen) to visualize anti-β-amyloid 6E10 immunofluorescent signal. For each plaque, because plaques varied in size, 4 points along the perimeter were recorded. The *X* and *Y* coordinates outlining the cortex were acquired by manual tracing in the MERSCOPE visualizer software (Vizgen) using DAPI signal and *Meg3*, *Cux2* and *Rorb* transcripts as landmarks. For all microglia within the cortex, the distance between each microglia and the nearest plaque was calculated using a custom Python script (find_nearest_neighbors2.py). The nearest-neighbour distance was calculated as the distance between the centre of the microglia and the edge of the plaque (defined using four points around the perimeter of each plaque). Microglial nuclei within 15 µm of a plaque were defined as ‘plaque-associated’, whereas those further than 15 µm from the nearest plaque were defined as ‘distal’. We used a larger distance cutoff for nuclei to account for the soma and processes.

The MERFISH analysis plots were generated using Seurat 5.0.3 and scCustomize 3.0.1 R packages. MERFISH-processed data have been deposited in Gene Expression Omnibus (GEO) and are publicly available as of the date of publication. Processed MERFISH.vzg files for browsing on MERSCOPE visualizer software (Vizgen) and the MERFISH raw output files are available upon request. Any further information required to reanalyse the data reported in this paper is available upon request.

### Isolation of adult microglial cells

For microglia phospho-protein analysis (Extended Data Fig. 6a), PU.1-low, wild-type and PU.1-high mice were euthanized by cervical dislocation, and brain regions were immediately removed. Frozen tissue was mechanically dissociated in glass homogenizers in Hank’s balanced salt solution (HBSS) supplemented with protease and RNase inhibitors. The homogenate was filtered through a 70-μm mesh filter. Myelin removal was performed using Percoll (pH 7.4) density gradient separation. The homogenate was supplemented with 90% Percoll (17-0891-02; Amersham) with PBS (pH 7.4). The resulting homogenate in a 21% Percoll gradient was centrifuged at 500*g* for 15 min at 4 °C. The pellet was washed and resuspended in HBSS. Microglia were gated as live cells expressing YFP^+^ in *Cx3cr1*^*CreErt2/+(Litt)*^ mice (Supplementary Fig. 6b). Roughly 120,000 cells were used per sample.

For ATAC-seq, wild-type, PU.1-low and PU.1-high mice were anaesthetized with an intraperitoneal injection of ketamine/xylazine, transcardially perfused with 12 ml of cold HBSS. The brains were collected, and the cerebellum and olfactory bulb were removed. Tissue was mechanically dissociated in glass homogenizers in HBSS. Myelin removal was performed using Percoll (pH 7.4; GE-17-0891-02) density gradient separation in 30% Percoll centrifuged at 500*g* for 15 min at 4 °C. The pellet was washed and resuspended in magnetic-activated cell sorting buffer (2 mM EDTA and 0.5% bovine serum albumin in 1× PBS) with DAPI and sorted on the FACS Aria II (BD Biosciences). Microglia were gated as live cells expressing YFP^+^ (Supplementary Fig. 6b). Roughly 50,000 cells were used per sample.

For single-cell sequencing and CD28 analysis, wild-type and 5xFAD mice were euthanized by CO_2_ asphyxiation, and brain regions were immediately removed. Fresh tissue was mechanically dissociated in glass homogenizers in HBSS and then supplemented with fluorescently conjugated antibodies (rat anti-mouse APC-CD11B; eBioscience; 17-0112-82; 1:100; and rat anti-mouse PerCP-Cy5.5-CD45; Invitrogen; 45-0451-82; 1:100) and incubated for 30 min on ice. Cells were washed twice (300*g* centrifugation for 5 min at 4 °C) and resuspended in FACS buffer (PBS and 2% bovine serum albumin). Cells were sorted on the FACS Aria II (BD Biosciences). Microglia were gated as live cells expressing CD11B^+^ and Cd45^low^ (Supplementary Fig. 1b). Roughly 250,000 (Extended Data Fig. 5a) and 700,000 (Extended Data Fig. 9b) cells were used per well for CD28 western blotting.

### Isolation of T cells

For ATAC-seq, wild-type mice were anaesthetized with an intraperitoneal injection of ketamine/xylazine and transcardially perfused with 12 ml of cold HBSS. Spleens were collected and homogenized on ice using a 70-µm strainer. Red blood cells were lysed with RBC lysis buffer (eBioscience; 00-4333-57) for 5 min at room temperature, followed by a PBS wash and centrifugation. Cells were resuspended in magnetic-activated cell sorting buffer, incubated with Fc blocker (Bio-Rad; BUF041B; 1:100) on ice for 10 min, stained with Thy1.2 (eBioscience; 17-0902-82; 1:100) and DAPI and sorted on the FACS Aria II (BD Biosciences). T cells were gated as live cells expressing THY1^+^. For western blot analysis, cells were snap-frozen and prepared, as described below. Roughly 250,000 (Extended Data Fig. 5a) cells were used for CD28 western blotting.

### Protein preparation and western blot analysis

For protein preparation from tissue, 6-month-old PU.1-low (three females and two males), control (two females and three males) and PU.1-high (three females) mice, as well as 6-month-old control (six males), 5xFAD (four males) and 5xFAD–PU.1-low mice (three males) were killed, and brain tissue was dissected and frozen immediately. The frozen brain tissue (cortex or hippocampus) was resuspended in 1% SDS (Ambion) supplemented with protease inhibitor (Roche), sonicated at 4 °C using a Bioruptor on high-power 30-s on/30-s off cycles for a total of ten cycles and boiled for 10 min in 1× lithium dodecyl sulfate sample buffer (Invitrogen) supplemented with dithiothreitol (DTT) to a final concentration of 200 mM (Sigma).

For protein preparation from cells, BV2 cells, iMgls, ex vivo microglia or T cells were centrifuged at 300*g* for 5 min at 4 °C. The supernatant was aspirated, and the cell pellet was snap frozen in liquid nitrogen. The BV2 cells were detached from the culture plate using PBS + 2 mM EDTA for 5 min at room temperature, whereas the primary microglia required further scraping. Protein was lysed in cold RIPA buffer (Thermo Fisher Scientific; 89900) with protease inhibitors (Roche; 11873580001), phosphatase inhibitors (Roche; 4906845001) and benzonase (MilliporeSigma; 71206) for 1 h on ice. Samples were then brought to 0.35 M NaCl, incubated for 1 h at 4 °C and spun at 14,000*g* at 4 °C for 15 min. The supernatant was moved to a fresh tube.

The protein concentration was determined using a bicinchoninic acid protein assay kit (Thermo Fisher Scientific) according to the manufacturer’s instructions. Protein samples were diluted in equal volumes of 2× lithium dodecyl sulfate sample buffer (Invitrogen) and supplemented with DTT to a final concentration of 200 mM (Sigma). Protein samples (20–40 μg) or concentrated culture supernatant (100 μg) was separated on 4–12% Bolt Bis-Tris precast denaturing gels or 10% NuPAGE Bis-Tris precast denaturing gels (Invitrogen) and transferred onto polyvinylidene difluoride membranes. For CD28 and PU.1 immunoblotting of primary microglia, NuPAGE Antioxidant (Invitrogen) was added to the running buffer. The membranes were probed with primary antibodies diluted in 5% bovine serum albumin (BSA)– or milk–TBST solution overnight at 4 °C. The membranes were then washed and probed with horseradish peroxidase-conjugated anti-mouse (GE; 1:10,000), anti-rabbit IgG secondary antibody (GE 1:10,000) or anti-sheep IgG antibody (Jackson ImmunoResearch; 1:10,000) for 1 h at room temperature. The membranes were developed using an enhanced chemiluminescence substrate (PerkinElmer) and exposed on film. Exposed films were scanned, and protein bands were quantified using ImageJ software (National Institutes of Health). Protein quantities were normalized using β-actin or histone H3. Full scans of western blots (uncropped) are provided in Supplementary Figs. 9–11.

The primary antibodies used were Syk (Cell Signaling Technology; 2712; 1:500), phospho-Syk Y352 (Cell Signaling Technology; 2717; 1:500), PLCG2 (Cell Signaling Technology; 3852; 1:500), phospho-PLCG2 Y1217 (Cell Signaling Technology; 3871; 1:500), PU.1 (Cell Signaling Technology; 2266; 1:500), CD28 (Abcam; ab243228; 1:500), ACTB (Abcam; ab8227; 1:2,000), H3 (Abcam; ab1791; 1:5,000), IBA1 (1:500; Wako; 016-20001) and C1Qa (1:500; Proteintech; 11602-1-AP).

### Proteomics analysis by mass spectrometry

Sample preparation and liquid chromatography–tandem mass spectrometry measurements were performed, as previously described^76^. In brief, BV2 cells were lysed in sodium deoxycholate buffer (1% sodium deoxycholate, 10 mM tris(2-carboxy (ethyl)phosphine), 40 mM 2-chloroacetamide and 100 mM Tris–HCl (pH 8.5)), heated at 95 °C for 10 min and sonicated to shear DNA. Proteins were digested overnight at 37 °C and 1,000 rpm using trypsin and LysC (1:100 enzyme/protein ratio; w/w). Digests were desalted using in-house-made SDB-RPS StageTips. Desalted peptides were dried in a vacuum concentrator and resolubilized in 0.1% formic acid. Concentrations were determined using a NanoDrop spectrophotometer and normalized between samples for equal peptide injection. Peptide mixtures were analysed using an EASY-nLC 1000 chromatographic system coupled to an Orbitrap Exploris 480 (Thermo Fisher Scientific). Peptides were separated by 120-min chromatographic gradients using a binary buffer system comprising buffer A (0.1% formic acid in liquid chromatography–mass spectrometry-grade water) and buffer B (80% acetonitrile and 0.1% formic acid in liquid chromatography–mass spectrometry-grade water) on a 50-cm in-house-made column (75-µm inner diameter) packed with 1.9-µm ReproSil C18 beads (Dr. Maisch GmbH) at a flow rate of 300 nl min^−1^ and 60 °C maintained by an in-house-made column oven. Spectra were acquired with data-independent acquisition using full scans with a range of 300–1,650 *m*/*z*.

### Bioinformatics analysis of mass spectrometry proteomics data

Data-independent acquisition mass spectrometry raw files were processed using DIA-NN^77^ (v.1.8) with FASTA digest for library-free search and deep learning-based spectra, retention time and ion mobility prediction enabled. Precursor false discovery rate was set to 1%, and default parameters were used with the following changes: the precursor range was restricted to 300–1,650 *m*/*z* and the fragment ion range to 200–1,650 *m*/*z*. The ‘--relaxed-prot-inf’ option was enabled through the command line. Match between runs was enabled, and the neural network classifier was set to ‘double-pass mode’ and the quantification strategy to ‘robust liquid chromatography (high accuracy)’. Spectra were matched against the UniProt FASTA database for mouse (December 2022 release).

Exported peptide data were filtered for a false discovery rate of less than 0.01 (‘*Lib.Q.Value*’ and ‘*Lib.PG.Q.Value*’) and proteotypic peptides before applying a sample-wise median normalization on log_10_ normalized precursor quantities and recovering the original value range. Protein intensities were normalized using the MaxLFQ^78^ algorithm using an in-house script, with a minimum of more than one peptide. Bioinformatics analyses were performed using Perseus^79^ (v.1.6.15.0) and R (v.4.1.2). Before performing statistical analysis, quantified proteins were filtered for at least three valid values in at least one group of replicates.

### RNA isolation and qPCR from primary microglia

Primary microglia (250,000) were lysed in TRIzol (Life Technologies) for 5 min, followed by phase separation with chloroform. After extraction, RNA was precipitated for 30 min at −20 °C in isopropanol with 0.15 M sodium acetate and GlycoBlue (Life Technologies). The pellet was then washed three times with 70% ethanol, air-dried and suspended in nuclease-free water. cDNA was prepared from total RNA using the High-Capacity RNA-to-cDNA Kit (Applied Biosystems). Relative gene expression of the cDNA was assayed by qPCR using recommended TaqMan gene expression assays (Life Technologies) and following the manufacturer’s recommendations (*Cd28*, *Ifnb*, *Mx1*, *Ifitm3*, *Gapdh* and *Actb*). Cycle counts for mRNA quantification were normalized to *Gapdh* or *Actb*. Relative expression (ΔCT) and quantification (*RQ* = 2^−ΔΔCT^) for each gene were calculated.

### RNA isolation and sequencing from iMgls

RNA was extracted using the RNeasy Plus Mini Kit (QIAGEN; 74136) following the manufacturer’s instructions. Libraries were prepared using the NEBNext Ultra II RNA Library Prep Kit following the manufacturer’s instructions. The quality of the libraries was assessed by a 2200 TapeStation (Agilent). Multiplexed libraries were directly loaded on NovaSeq X Plus (Illumina) with paired-end sequencing. Raw sequencing data were processed using Illumina bcl2fastq2 Conversion Software v.2.17.

### Bioinformatics analysis of iMgl RNA sequencing

Raw sequencing reads were first quality checked and trimmed using Trim Galore (https://www.bioinformatics.babraham.ac.uk/projects/trim_galore/ v.0.6.4, a wrapper program implementing Cutadapt v.2.9 (https://journal.embnet.org/index.php/embnetjournal/article/view/200) and FastQC v.0.11.9 (https://www.bioinformatics.babraham.ac.uk/projects/fastqc/) and mapped to the human genome (hg38) using the HISAT2 package (v.2.2.0) (ref. ^80^). Reads were counted using featureCounts (v.2.0.0) (ref. ^81^) against the Ensembl 99 annotation. The raw counts were processed through a VST procedure using the DESeq2 package^82^ to obtain transformed values that were more suitable than the raw read counts for certain data mining tasks. A pairwise comparison was performed on the count data of entire gene transcripts using the DESeq2 package (v.1.36.0) (ref. ^82^). Heat maps were made using R (v.3.1.1; https://www.R-project.org), in which the expression of each gene in VST was normalized to the mean across all samples (*z*-scored).

### Assay for transposase-accessible chromatin sequencing

ATAC-seq libraries were prepared using the Omni-ATAC protocol^83–85^. In brief, nuclei were isolated from 50,000 isolated YFP^+^ ex vivo microglia or THY1.2^+^ T cells. Transposition was performed using Tn5 transposase (Illumina) at 37 °C for 30 min, followed by DNA purification using the ZYMO DNA Clean & Concentrator Kit. Libraries were pre-amplified for five cycles using NEBNext High-Fidelity 2X PCR Master Mix. Further amplification cycles were determined through qPCR. Double-sided size selection was performed using AMPure XP beads. Libraries were quantified through Qubit and analysed using Bioanalyzer or TapeStation. Final libraries were pooled equimolarly and sequenced on an Illumina NovaSeq (2 × 150 bp), targeting approximately 40 million reads per sample.

### Bioinformatics analysis of ATAC-seq data

A total of 11 ATAC-seq libraries from PU.1-low microglia (*n* = 3), PU.1-high microglia (*n* = 3), wild-type microglia (*n* = 3) and wild-type T cells (*n* = 2) were processed using the same pipeline for compatibility. Read quality was assessed using FastQC (v.0.11.8)^86^. Reads were trimmed for adaptor sequences using Trim Galore (v.0.6.6)^87^ and aligned to the mouse reference genome (GRCm38; GENCODE M25) using Bowtie 2 (v.2.2.8) with a maximum fragment length of 2,000 bp (−X 2000) with default sensitivity settings^88^. Reads aligned to mitochondrial DNA and non-canonical chromosomes were removed. Picard (v.2.2.4) was used to remove duplicated reads. Post-filtering Binary Alignment Map files for all samples were merged using the merge function from Samtools (v.1.11), followed by peak calling using MACS2 (v.2.1.0) with parameters --nomodel, --nolambda, --keep-dup all and --slocal 10000, optimized for paired data (−f BAMPE) using the mouse genome (−g mm)^89^. Blacklisted regions were removed before downstream analysis. Peak summits were extended ±200 bp, and quantification of reads in peaks was performed using featureCounts from Subread (v.2.0.1). Peaks that fell below the top 67% of peaks across all samples were filtered out as low-signal regions. Coverage tracks were generated from Binary Alignment Map files using deepTools (v.3.2.1) bamCoverage with parameters –normalizeUsingRPKM –binsize 10 (ref. ^90^).

Differential accessibility in the analysis was performed using the DESeq2 (v.1.34.0) R package^82^. A peak was considered differentially accessible if the Benjamini–Hochberg adjusted *P* value < 0.05, and the absolute log_2_ fold change was greater/lower than 1.

Heat maps of the union of all differentially accessible regions across multiple comparisons were generated using heatmaply (v.1.2.1), displaying DESeq2 VST-normalized ATAC read counts. Read counts were *z*-scored by row, and hierarchical clustering was applied to rows only. Heat maps and profile plots of peak signals were generated using deepTools (v.3.2.1) in reference-point mode to visualize peak signals across differentially accessible regions using RPKM-normalized bigWig files as input^90^.

For peak-to-gene associations, annotated peaks were obtained using ChIPseeker (v.1.30.3). Peaks were first converted into a GRanges object using the makeGRangesFromDataFrame() function from GenomicRanges (v.1.46.1), retaining all relevant metadata (keep.extra.columns=TRUE). Peak annotation was performed with the annotatePeak() function using a TxDb object derived from the mouse reference genome (GRCm38; GENCODE M25). Peaks were mapped to gene features at the ‘gene’ annotation level, with the transcription start site region defined as ±1,000 bp.

Principal component analysis (PCA) variance-stabilized counts were generated using DESeq2 (v.1.34.0) and used as input for PCA. For each analysis, the top 5,000 most variable peaks were selected on the basis of row variance using matrixStats (v.0.62.0.9003). PCA was performed using prcomp in R (v.4.1.0). For visualization of microglia-only samples, PCA plots were generated using Plotly (v.4.9.4.1). To visualize both microglia and T cells in a combined PCA space, the first two principal components (PC1 and PC2) were plotted using ggplot2 (v.3.3.5) with fixed axis scaling to preserve relative distances.

Transcription factor motif enrichment was done using HOMER (v.4.10). The findMotifsGenome.pl tool was applied to significant peaks within 200 bp around the peak summit using the parameter −size 200. Motifs were considered on the basis of *P* value (*P* < 1 × 10^−12^). HOMER de novo motifs are shown.

### Ribosome profiling by translating ribosome affinity purification

The TRAP approach^91,92^ relies on the genetic labelling of the ribosomal protein L10a with the enhanced green fluorescent protein (eGFP) using *Eef1a1*^*LSL.eGFPL10a/+*^ mice^60^. Crossing these mice to microglia-specific Cre mice allows for the isolation of ribosome-associated mRNAs in a cell-type-specific fashion using eGFP-based immunoaffinity purification^28,91,93^. *Cx3cr1*^*CreErt2/+(Litt)*^*;Eef1a1*^*LSL.eGFPL10a/+*^ control (three females and three males), *Cx3cr1*^*CreErt2/+(Litt)*^*;*5xFAD;*Eef1a1*^*LSL.eGFPL10a/+*^ (three females and three males on control diet or two females and two males on PLX5622 CSF1Ri diet), *Cx3cr1*^*CreErt2/+(Litt)*^*;Spi1*^*fl/+*^*;Eef1a1*^*LSL.eGFPL10a/+*^ PU.1-low mice (four females and two males), *Cx3cr1*^*CreErt2/+(Litt)*^*;R26*^*FLAG-Spi1/+*^*;Eef1a1*^*LSL.eGFPL10a/+*^ PU.1-high mice (three females and one male), *Cx3cr1*^*CreErt2/+(Litt)*^*;*5xFAD;*Spi1*^*fl/+*^*;Eef1a1*^*LSL.eGFPL10a/+*^ 5xFAD–PU.1-low (one female and three males) and *Cx3cr1*^*CreErt2/+(Litt)*^*;*5xFAD;*R26*^*FLAG-Spi1/+*^*;Eef1a1*^*LSL.eGFPL10a/+*^ 5xFAD–PU.1-high mice (one female and two males) were euthanized with CO_2_, and brain ROI were dissected. Ribosome-associated mRNA from microglia was isolated from each region, as previously described^92^, in which each sample corresponds to a single mouse. Briefly, the brain tissue was thawed in an ice-cold Wheaton 33 low extractable borosilicate glass homogenizer containing 1-ml cell lysis buffer (20 mM HEPES–KOH (pH 7.3), 150 mM KCl, 10 mM MgCl_2_, 1% NP-40, 0.5 mM DTT, 100 μg ml^−1^ of cycloheximide and 10 μl ml^−1^ of RNasin (Promega) and SUPERase·In (Invitrogen). The samples were first manually homogenized using a polytetrafluoroethylene homogenizer (grinding chamber clearance of 0.1–0.15 mm) with three to five gentle strokes, followed by homogenization in a motor-driven overhead stirrer at 900 rpm with 12 complete strokes. The samples were then transferred to chilled Eppendorf tubes. A post-nuclear supernatant was prepared by centrifugation at 2,000*g* for 10 min at 4 °C. NP-40 (final concentration of 1%) and DHPC (final concentration of 30 mM) were added to the supernatant. A post-mitochondrial supernatant was prepared by centrifugation at 16,000*g* for 10 min at 4 °C. Dynabeads MyOne Streptavidin T1 (200 μl; Invitrogen), conjugated to 1 μg μl^−1^ of biotinylated Protein L (Pierce) and 50 μg each of anti-eGFP antibodies Htz–GFP–19F7 and Htz–GFP–19C8 (bioreactor supernatant from the Memorial Sloan Kettering Cancer Center Monoclonal Antibody Facility) was added to each supernatant. The unbound fraction was collected using a magnetic stand after overnight incubation at 4 °C with gentle end-over-end rotation. The polysome-bound beads were washed with a high-salt buffer (20 mM HEPES–KOH (pH 7.3), 350 mM KCl, 10 mM MgCl_2_, 1% NP-40, 0.5 mM DTT and 100 μg ml^−1^ of cycloheximide).

RNA clean-up from isolated microglial cells (50,000), TRAP samples and 5% of the unbound fractions from TRAP samples was performed using RNeasy Mini Kit (QIAGEN) following the manufacturer’s instructions. RNA integrity was assayed using an RNA Pico chip on a Bioanalyzer 2100 (Agilent), and only samples with RNA integrity number greater than 9 were considered for subsequent analysis. Double-stranded cDNA was generated from 1–5 ng of RNA using the Ovation V2 kit (NuGEN) following the manufacturer’s instructions. Libraries for sequencing were prepared using the Nextera XT Kit (Illumina) following the manufacturer’s instructions. The quality of the libraries was assessed by a 2200 TapeStation (Agilent). Multiplexed libraries were directly loaded on NovaSeq (Illumina) with single-read sequencing for 75 cycles. Raw sequencing data were processed using Illumina bcl2fastq2 Conversion Software v.2.17.

### Bioinformatics analysis of TRAP sequencing

Raw sequencing reads were first quality checked and trimmed using Trim Galore (https://www.bioinformatics.babraham.ac.uk/projects/trim_galore/ v.0.6.4, a wrapper program implementing Cutadapt v.2.9 https://journal.embnet.org/index.php/embnetjournal/article/view/200 and FastQC v.0.11.9 https://www.bioinformatics.babraham.ac.uk/projects/fastqc/) and then mapped to a mouse genome (mm10) using the HISAT2 package (v.2.2.0)^80^. Reads were counted using featureCounts (v.2.0.0)^81^ against the Ensembl 99 annotation. The raw counts were processed through a VST procedure using the DESeq2 package^82^ to obtain transformed values that were more suitable than the raw read counts for certain data mining tasks. PCA was performed on the top 500 most variable genes across all samples on the basis of the VST data to assess for visual outliers. All pairwise comparisons were performed on the count data of entire gene transcripts using the DESeq2 package (v.1.36.0)^82^.

For comparisons of baseline samples with 5xFAD samples, a cutoff of the adjusted *P* value < 0.1 and mean expression greater than 10 (DESeq2) was applied. For comparisons involving drug treatments, genetic manipulations and ageing, a cutoff of uncorrected *P* value < 0.05 and mean expression greater than 10 was applied (DESeq2). Additionally, TRAP-enriched genes were calculated using a cutoff of *P* value < 0.05 and fold change greater than 2 over their respective unbound fraction. All heat maps and scatter plots for bulk sequencing were made using R (v.3.1.1; https://www.R-project.org). For all heat maps, the expression of each gene in VST was normalized to the mean across all samples (*z*-scored).

Gene Ontology term enrichment, cell type enrichment and kinase co-expression ARCHS4 (ref. ^94^) analyses were performed using Enrichr^95–97^. Pathway and upstream regulator analyses were performed using Ingenuity Pathway Analysis software (v.01-22-01; QIAGEN). Selected and significantly enriched (*P* value < 0.05 with Benjamini–Hochberg correction) Gene Ontology terms for biological processes are represented in balloon graphs and were made using R (v.3.1.1; https://www.R-project.org). Pie charts for functional characterization of genes were built manually on the basis of Gene Ontology term enrichment analyses and a literature search. Gene expression data were further analysed using Gene Set Enrichment Analysis (GSEA 4.2.3 software; number of permutations = 1,000) using the DAM gene set from Keren-Shaul et al.^12^ (Supplementary Table 3).

### Isolation of lineage-traced microglial nuclei for single-nucleus sequencing

Microglial nuclei were isolated from microglia-specific TRAP mice bred to *Eef1a1*^*LSL.eGFPL10a/+*^ mice^60^ on the basis of the eGFP–L10a fluorescence of newly formed ribosomes in the microglia nucleoli, as described^28^. In brief, 6-month-old *Cx3cr1*^*CreErt2/+(Litt)*^*;*5xFAD;*Eef1a1*^*LSL.eGFPL10a/+*^ mice (one female on control diet or two males on PLX5622 diet) or *Cx3cr1*^*CreErt2/+(Litt)*^*;*5xFAD*;Eef1a1*^*LSL.eGFPL10a/+*^ 5xFAD (female), *Cx3cr1*^*CreErt2/+(Litt)*^*;*5xFAD;*Spi1*^*fl/+*^*;Eef1a1*^*LSL.eGFPL10a/+*^ 5xFAD–PU.1-low mice (male) and *Cx3cr1*^*CreErt2/+(Litt)*^*;*5xFAD; *R26*^*FLAG-Spi1/+*^*;Eef1a1*^*LSL.eGFPL10a/+*^ 5xFAD–PU.1-high mice (male) were euthanized with CO_2_. Brain regions were quickly dissected and frozen immediately. The frozen cortex tissue was homogenized in 0.25 M sucrose, 150 mM KCl, 5 mM MgCl_2_ and 20 mM tricine (pH 7.8), supplemented with protease and RNase inhibitors with a glass Dounce homogenizer (1984-10002; Kimble Chase). The buffers were supplemented with 10 μl ml^−1^ of RNasin, SUPERase·In and EDTA-free protease inhibitor cocktail (11836170001; Roche). The homogenate was then spun through a 29% iodixanol cushion. The resulting nuclear pellet was resuspended in 0.25 M sucrose, 150 mM KCl, 5 mM MgCl_2_ and 20 mM tricine (pH 7.8), supplemented with 10 μM DyeCycle Ruby (V10304; Invitrogen) and 10% donkey serum (017-000-121; Jackson ImmunoResearch). Microglial nuclei were sorted in a BD FACSAria Cell Sorter by gating for the lowest DyeCycle Ruby, which indicates nuclei singlets and a high GFP signal. For single-nucleus RNA-seq, isolated nuclei were used immediately. More than 10,000 nuclei were used for 10× sequencing.

### Microglia-specific single-cell and single-nucleus sequencing

We used the Chromium platform (10x Genomics) with the 3′ gene expression v.2 kit with a targeted input of 5,000 nuclei or cells per sample on average. In brief, gel bead in emulsions (GEMs) were generated on the sample chip in the Chromium controller. Barcoded cDNA was extracted from GEMs using Post GEM-RT Cleanup and amplified for 12 cycles. The amplified cDNA was fragmented and subjected to end-repair, poly-A-tailing, adaptor ligation and 10x-specific sample indexing following the manufacturer’s protocol. Libraries were quantified using Bioanalyzer (Agilent) and Qubit (Thermo Fisher Scientific) analyses and then sequenced in paired-end mode on a HiSeq 2500 instrument (Illumina) targeting a depth of 50,000–100,000 reads per nucleus per cell.

### Bioinformatics analysis of single-cell and single-nucleus sequencing

The raw read data were demultiplexed, aligned and analysed using 10x Cell Ranger (v.2.1.0). To capture the unspliced pre-mRNA in the single-nucleus RNA expression assay, intronic regions in the 10x Cell Ranger mm10 v.1.2.0 reference were marked as exonic, as suggested by 10x for pre-mRNA reference generation. Data from two experiments were used to generate Seurat objects in R. Quality control cutoffs (nFeature of more than 1,500; pct.mt of less than 5) were used to generate final datasets. The following clustering pipeline steps were applied: NormalizeData(), FindVariableFeatures(), ScaleData(), RunPCA(), FindNeighbors() and FindClusters(). Clusters that do not express *Hexb* (highly expressed pan-microglia gene marker) were excluded. Cluster quality was confirmed by VlnPlot() for nFeature, nCount and pct.mt (percentage of mitochondrial DNA). Three datasets were generated from single-cell RNA-seq: control versus 5xFAD (8-month-old mice; Figs. 1a and 2a, Extended Data Fig. 3a,b and Supplementary Fig. 1), time course of control and 5xFAD (Fig. 5a and Extended Data Fig. 10c) and control, 5xFAD versus 5xFAD;Cd28-KO (Fig. 5d,e, Extended Data Fig. 11e and Supplementary Fig. 8).

For single-cell sequencing (Figs. 1a, 2a and 5a,d,e, Extended Data Figs. 3a, 10c,d and 11e and Supplementary Figs. 1 and 8), cell counts were as follows: 3-month-old control (5,240 cells), 3-month-old 5xFAD (4,172 cells in replicate 1 and 2,966 cells in replicate 2), 3-month-old 5xFAD;Cd28-KO (2,466 cells in replicate 1 and 2,812 cells in replicate 2), 6-month-old control (6,249 cells), 6-month-old 5xFAD (4,351 cells), 6-month-old 5xFAD;CD28-KO (4,361 cells), 8-month-old control (351 cells in replicate 1 and 6,635 cells in replicate 2) and 8-month-old 5xFAD (785 cells in replicate 1 and 7,539 cells in replicate 2). The following clustering pipeline steps were applied: FindIntegrationAnchors() with dims = 1:20, IntegrateData() with dims = 1:2, ScaleData(), FindVariableFeatures() with selection.method = ‘vst’, nFeature = 3,000, RunPCA() with npcs = 30, RunUMAP() with reduction = ‘pca’ and dims = 1:20, FindNeighbors() with reduction = ‘pca’ and dims = 1:20 and FindClusters() with resolution = 0.6. Cluster marker genes were identified using FindAllMarkers() with logfc.threshold = 0.25 and min.pct = 0.0. Sub-clustering of DAM microglia in Fig. 1a and Supplementary Fig. 1 was performed after unbiased cell clustering and manual annotation of cluster identification on the basis of genes (*Cst7*, *Apoe*, *Lpl* and *Itgax*) driving the clustering. For the sub-clustering of DAM microglia, the DAM cluster was isolated and reclustered into three sub-clusters following the clustering pipeline steps: ScaleData(), FindVariableFeatures() with selection.method = ‘vst’, nFeature = 3,000, RunPCA() with npcs = 30, RunUMAP() with reduction = ‘pca’ and dims = 1:20, FindNeighbors() with reduction = ‘pca’ and dims = 1:20 and FindClusters() with resolution = 0.4. Further differential analysis was performed using the Seurat package in R. Differential expression between the *Spi1*^low^ and *Spi1*^high^ (PU.1^low^ and PU.1^high^) clusters was performed using FindMarkers(), with a log_2_fc cutoff of 0.25, with min.pct = 0.01. Differential expression between the homeostatic and DAM clusters was performed using the ‘FindMarkers’ function, with a log_2_fc cutoff of 0.25 and min.pct = 0.01. For pseudobulk analysis comparing genotypes in Fig. 2a and Extended Data Fig. 11e, Seurat identities were set to ‘genotype’ using the feature Idents(). Pseudobulk analysis was performed with the FindMarkers() function with a Wilcoxon rank-sum test in Seurat with the following parameters: min.pct = 0.01 and logfc.threshold = 0.25.

For single-nucleus sequencing (Fig. 1e, Extended Data Fig. 3c and Supplementary Fig. 2), data were obtained from 6-month-old 5xFAD mice fed with control diet (7,286 nuclei) and 6-month-old 5xFAD mice fed with CSF1Ri diet (3,995 nuclei). The following clustering pipeline steps were applied: Merge(), ScaleData(), FindVariableFeatures with nFeature = 3,000, RunPCA() with npcs = 30, RunUMAP() with dims = 1:20, FindNeighbors with dims = 1:20 and FindClusters() with resolution = 0.6. Cluster marker genes were identified using FindAllMarkers() with logfc.threshold = 0.25 and min.pct = 0.0. Sub-clustering of DAM microglia (Fig. 1e and Supplementary Fig. 2) was performed in the same fashion as single-cell sequencing. In brief, the DAM cluster was isolated and reclustered into three sub-clusters by applying the following clustering pipeline steps: ScaleData(), FindVariableFeatures() with selection.method = ‘vst’, nFeature = 3,000, RunPCA() with npcs = 30, RunUMAP() with reduction = ‘pca’ and dims = 1:20, FindNeighbors() with reduction = ‘pca’ and dims = 1:20 and FindClusters() with resolution = 0.3. Differential expression between all microglia from the control and CSF1Ri-treated mice (Extended Data Fig. 3e) was performed using pseudobulk analysis. Seurat identities were set to ‘genotype’ using the feature Idents(). Pseudobulk analysis was performed with the FindMarkers() function with a Wilcoxon rank-sum test in Seurat with the following parameters: min.pct = 0.0 and logfc.threshold = 0.25.

For single-nucleus sequencing (Extended Data Fig. 7), cell counts were as follows: 6-month-old 5xFAD (4,541 cells), 6-month-old 5xFAD;PU.1-low (4,866 cells) and 6-month-old 5xFAD;PU.1-high (6,190 cells). The following clustering pipeline steps were applied: FindIntegrationAnchors() with dims = 1:40, IntegrateData() with dims = 1:2, ScaleData(), FindVariableFeatures() with nFeature = 1,000, RunPCA(), RunUMAP() with dims = 1:20, FindNeighbors() with dims = 1:20 and FindClusters() with resolution = 0.5. Cluster marker genes were identified using FindAllMarkers() with logfc.threshold = 0.2 and min.pct = 0.05.

To perform differential expression analysis of PU.1^low^ and PU.1^high^ microglia in independent experiments, the filtered UMI count matrices from two independent experiments, each containing one control and one 5xFAD sample, were loaded into R (v.4.3.2)^98^ using SingleCellExperiment^99^ package (v.1.24.0) and Seurat (v.5.0.1)^100^. Low-quality cells and presumed doublets were removed using the R package Scatter^101^ (v.1.30.1). Non-microglia cells were identified by Seurat clustering after SCTransform^102,103^ on the basis of the expression of known microglia signature genes *Hexb* and *Fcer1g*. For each of the 5xFAD samples, one PU.1^high^ cluster and one PU.1^low^ cluster of cells were identified by Seurat clustering on the basis of known marker genes (PU.1^high^: *Spi1*, *Il7r*, *Csf1r*, *Cd33*, *Tlr4*, *Il1a* and *C3ar1*; PU.1^low^: *Cst7*, *Cd274*, *Igf1*, *Apoe*, *Spp1*, *Csf1*, and *Pdcd1*). The differential expression analyses between PU.1^high^ and PU.1^low^ clusters were conducted with the function ‘findMarkers()’ of the Seurat package with default parameters, except min.pct = 0.01, on ‘data’ layer of ‘RNA’ assay after ‘NormalizeData()’ transformation. The log_2_ fold changes of overlapping differentially expressed genes (DEGs) were compared between the 5xFAD samples in experiments 1 and 2 in a scatter plot highlighting key genes related to lymphoid or myeloid lineage or inflammation.

In single-cell sequencing of 5xFAD;CD28-KO, we observed a paradoxical increase in *Cd28* mRNA expression. This increased expression is seen randomly across all microglial clusters and is likely to reflect mRNA expression downstream of the deleted exons 2 and 3 in the CD28-KO. The ablation of *Cd28* RNA and CD28 protein in these mice was confirmed using in situ hybridization (Extended Data Fig. 11f) and immunofluorescence protein staining (Fig. 5b).

Similarly, we did not detect significant differences in *Spi1* level in single-nucleus sequencing data. Instead, upon removing one allele of the *Spi1* gene, the non-microglia-expressed, co-regulated and adjacent *Mybpc3* gene^24^ was upregulated, which serves as a positive indicator of *Spi1* downregulation. Changes in PU.1 protein levels in PU.1-low and PU.1-high mice have been validated by western blotting (Extended Data Fig. 4b) and immunostaining (Fig. 3a).

For all experiments, plots were generated using the DimPlot(), FeaturePlot(), VlnPlot() and DotPlot() functions. Lymphoid (*Cd28*, *Cd274*, *Pdcd1*, *Ctla2a*, *Cd5*, *Sox5*, *Cd48*, *Cd52* and *Cd72*) and interferon (*Ifitm3*, *Ifit3*, *Isg15*, *Ifit2*, *Ifi27l2a*, *Ifi204*, *Irf7*, *Usp18*, *Stat2*, *Ifitm2*, *Rsad2*, *Isg15*, *Ifit1*, *Bst2*, *Isg20* and *Xaf1*) scores were calculated using the AddModuleScore() function. Volcano plots were made using R.

### Bioinformatics analysis of publicly available data

For the analysis of human microglia scRNA-seq^15^ (shown in Fig. 2e and Supplementary Fig. 5), data were obtained from Synapse syn21438358. The gene count matrix and corresponding cell barcodes for human ex vivo microglia were processed using a similar approach, as described by the authors. Briefly, UMI counts and cell annotations were imported into Seurat v.5.0.1 for downstream analysis. Cells annotated as non-microglial by Olah et al.^15^ (such as monocytes, T cells, B cells, red blood cells and GFAP-positive cells) were excluded to focus on microglial cell populations. Data normalization and scaling were performed using the Seurat default parameters and included regression of UMI counts to account for technical noise. Subsequently, variable features were identified, and clustering was performed using the default Seurat settings, with a final resolution of 0.6 to determine cluster identities. Clusters were visualized using UMAP. Gene set module scores were computed per cell using the AddModuleScore function in Seurat to assess the relative expression of predefined gene sets, DAM, homeostatic and lymphoid genes (Supplementary Fig. 5). Clusters were categorized into two main groups (homeostatic and DAM) on the basis of the average ratio of DAM to homeostatic module scores. The DAM group was further subdivided on the basis of the expression of lymphoid marker genes. DAM clusters were categorized as either lymphoid-positive or lymphoid-negative, depending on the levels of lymphoid-related gene expression. Finally, gene expression patterns and functional module scores across clusters were visualized using dot plots and UMAP projections to identify and compare gene signatures associated with different microglial clusters. New clusters were annotated on the basis of marker gene expression profiles and compared to the original Olah et al.^15^ cluster annotations. A Sankey plot was generated to visualize the redistribution of cells from original clusters to new clusters using the scmap R package. A confusion matrix was constructed to quantify the percentage overlap between the two clustering schemes. For each original cluster, the percentage of its cells found in each new cluster was calculated and plotted using a heat map, with a blue colour gradient indicating increasing overlap.

The analysis of human microglia scRNA-seq data^17^ (shown in Fig. 5f,g) is, on the whole, based on data obtained from the AD Knowledge Portal (https://adknowledgeportal.org)^104^. Single-nucleus RNA-seq counts and cell-level metadata were downloaded from Synapse syn31512863 (ref. ^17^). All downstream processing and statistical tests were performed using R statistical software v.4.3.1 (ref. ^98^). To generate pan-microglial pseudobulks, the authors’ cell annotations were used to filter microglia. Because most of the samples from the same donor were sequenced in two replicate batches, we further subsetted the data to include only those donors and batches and generated pseudobulks by summing read counts for all cells for each donor. Samples with at least ten cells were retained for further analysis (*n* = 428). Similarly, to generate cluster-specific pseudobulks, the same subset of donors and batches was included. Counts were summed for all cells in a cluster for each donor, and we retained samples with at least ten cells per cluster (*n* = 1,963). For visualization, counts were CPM normalized with edgeR^105^ and batch corrected with the regressBatches function from the batchelor package^106^. Heat maps were generated with the pheatmap package^107^.

*SPI1*–AD risk variant differential expression and DAM abundance analysis were done after donor genotypes were downloaded from the AD Knowledge Portal^104^. Pseudobulks were annotated by matching on the ‘projid’ included in the cell metadata and searching for rs1057233 genotype information^108^. On inspection of the data, we noted that rs1057233 and *SPI1* expression showed a dominant inheritance pattern, in which donors with two copies of the risk-associated A allele showed slightly increased *SPI1* expression compared with heterozygous carriers and non-carriers. We, therefore, labelled homozygotes as ‘risk’ and others as ‘non-carriers’.

Differential expression and DAM abundance analyses were performed on samples with diagnoses (cogdx) of either not cognitively impaired or AD and for which *SPI1* variant status was known (*n* = 302 for differential expression and *n* = 306 for DAM abundance). For differential expression on the basis of carrier status, limma-voom^109^ was run on donor-level pseudobulks with batch as a blocking factor and AD status as a grouping variable. For the DAM abundance analysis, the proportion of microglia in the Mic 13 cluster was calculated for each donor, and a two-way ANOVA was performed to analyse the effect of cognitive diagnosis for AD and *SPI1* carrier status on the proportion of DAM microglia. This was followed by Tukey’s post hoc test, with *P* < 0.05 considered significant.

For the bioinformatics analysis of Kimura et al.^49^, mouse microglia single-nucleus sequencing data were obtained on GEO with accession number GSE267764 and converted to Seurat objects. Quality control cutoffs (nFeature greater than 1,500; pct.mt less than 5) were used to generate final datasets. The following clustering pipeline steps were then applied: NormalizeData(), FindVariableFeatures(), ScaleData(), RunPCA(), FindNeighbors() and FindClusters(). Clusters that do not express *Hexb* (highly expressed pan-microglia gene marker) were excluded. Cluster quality was confirmed by VlnPlot() for nFeature, nCount and pct.mt (percentage of mitochondrial DNA). Data were then integrated using the following clustering pipeline steps: FindIntegrationAnchors() with dims = 1:20, IntegrateData() with dims = 1:2, ScaleData(), FindVariableFeatures() with selection.method = ‘vst’, nFeature = 3,000, RunPCA() with npcs = 30, RunUMAP() with reduction = ‘pca’, dims = 1:20, FindNeighbors() with reduction = ‘pca’, dims = 1:20 and FindClusters() with resolution = 0.6. Nineteen clusters were generated; cluster-identifying markers were generated using the FindMarkers() with logfc.threshold = 0.25 and min.pct = 0.

### Electrophysiological studies for long-term potentiation in hippocampal slices

Sex-matched control, 5xFAD and 5xFAD;PU.1-low mice (9–10 months old) were deeply anaesthetized with isoflurane and subsequently decapitated. Brains were rapidly removed and chilled in cutting artificial CSF (ACSF) containing choline chloride (93 mM), HCl (93 mM), KCl (2.5 mM), NaH_2_PO_4_ (1.2 mM), NaHCO_3_ (30 mM), HEPES (20 mM), glucose (25 mM), sodium ascorbate (5 mM), thiourea (2 mM), sodium pyruvate (3 mM), MgSO_4_ (10 mM) and CaCl_2_ (0.5 mM; pH 7.4). The brains were embedded in 2% agarose, and coronal slices (400 µm thick) were made using a Compresstome (Precisionary Instruments). Brain slices were then allowed to recover at 32 ± 1 °C in cutting solution for 5 min and after that at room temperature in holding ACSF containing NaCl (92 mM), KCl (2.5 mM), NaH_2_PO_4_ (1.2 mM), NaHCO_3_ (30 mM), HEPES (20 mM), glucose (25 mM), sodium ascorbate (5 mM), thiourea (2 mM), sodium pyruvate (3 mM), MgSO_4_ and CaCl_2_ (2 mM; pH 7.4). After at least 2 h of recovery, the slices were transferred to a submersion recording chamber and continuously perfused (2–4 ml min^−1^) with ACSF containing NaCl (124 mM), KCl (2.5 mM), NaH_2_PO_4_ (1.2 mM), NaHCO_3_ (24 mM), HEPES (5 mM), glucose (12.5 mM), MgSO_4_ (2 mM) and CaCl_2_ (2 mM; pH 7.4). Slices were allowed to recover for another hour in the recording chamber before recording the baseline. All the solutions were continuously bubbled with 95% O_2_/5% CO_2_, and recordings were obtained at 25 ± 1 °C.

A bipolar stimulating electrode was positioned in the stratum radiatum of the CA1 region. Field excitatory postsynaptic potentials were evoked by square-wave 50-µs pulses delivered every 30 s by MultiClamp 7b (Molecular Devices), and current was set to 50% of the maximum field excitatory postsynaptic potential amplitude using ISO-Flex (ampi). A recording electrode, filled with ACSF (2–4 MΩ), was placed in the stratum radiatum a few millimetres away from the stimulating electrode. LTP was induced using a theta-burst protocol: four trains, separated by 10 s, each comprised five bursts of four stimuli delivered at 100 Hz, with 200 ms between the bursts. Data were acquired using a MultiClamp 7B amplifier (Molecular Devices), filtered at 3 kHz, digitized at 10 kHz and analysed using pCLAMP 11 software (Molecular Devices). LTP was quantified as a percentage change in excitatory postsynaptic potential slope from baseline (the last 10 min of a 30-min baseline period).

### Behavioural assays

Behavioural analyses used sex-matched control, 5xFAD and 5xFAD;PU.1-low mice (9–10 months old). All behavioural analyses were performed within the 0700–1900 hours light cycle. All experiments were conducted in a blinded manner, and genotypes were decoded only after data processing and analysis. The mice were habituated to the testing room for at least 1 h before the experiment. All procedures followed the National Institutes of Health Guide for the Care and Use of Laboratory Animals and were approved by the Institutional Animal Care and Use Committee at the Icahn School of Medicine at Mount Sinai. GraphPad Prism (GraphPad software; v.9.0) was used for statistical data analysis.

#### Open field

Locomotion and exploratory behaviour were measured using the open-field assay, as previously described^28^. Open-field activity was quantified using Fusion software (Omnitech Electronics). The mice were recorded for 60 min, and chambers were thoroughly cleaned with ethanol between rounds. Anxiety was assessed by measuring the total time spent in the centre of the open-field box for the first 10 min of the 60-min recording.

#### Novel object recognition

Mice were habituated to a three-room novel object box for 20 min without objects. During the training trial, the mice were placed in the box containing two identical objects and allowed to explore for 10 min. They were then returned to their home cage for a 1-h inter-test interval. During the testing trial, the mice were placed in the box containing one familiar object from the training phase and one novel object. Between each round, the box was thoroughly cleaned with ethanol. Videos of each session were measured using EthoVision video-tracking software (Noldus Information Technology). Blinded manual scoring was performed for the time spent sniffing the familiar and novel objects. Recognition score was calculated as the percentage of time spent sniffing the novel object relative to the total time spent sniffing either object.

#### Survival

Control, 5xFAD, 5xFAD;PU1-low and 5xFAD;PU.1-high mice were monitored for survival and evaluated for health status at least once per week starting at tamoxifen induction (4–6 weeks of age) and continuing until death. Mice that were used for the experiments or died from environmental factors (such as cage flooding) were censored and excluded from death rate calculations. Both male and female mice were included in the study. No sex-specific differences in survival outcomes were observed; therefore, data from both sexes were pooled for analysis. Survival curves were generated using the Kaplan–Meier method, and statistical differences between groups were assessed using log-rank (Mantel–Cox) tests.

### Statistical analysis

Statistics were analysed using GraphPad Prism v.5.01, with significance determined as *P* < 0.05. All statistical analyses were two-tailed. Normal distribution was assessed using the Shapiro–Wilk normality test. Grubbs’s test was used to identify outliers. Variance was determined using the *F*-test. An unpaired *t*-test was used for normally distributed datasets with two independent samples. For normally distributed datasets with two dependent samples (for example, plaque-associated and distal microglia from the same mice), we used a paired *t*-test. For normally distributed datasets with more than two samples, we used one-way ANOVA with multiple comparisons. Normally distributed data with unequal variance were analysed using Welch’s correction. Non-normally distributed data involving two samples were analysed using a two-tailed Mann–Whitney *U*-test. Non-normally distributed data involving more than two samples were analysed using a two-tailed Kruskal–Wallis test. No statistical methods were used to predetermine sample size, but our sample sizes were similar to those generally used in the field.

### Sample sizes, data exclusion, replication, randomization and blinding

We did not include a justification of the sample size for this study. We used the minimum number of animals needed to reliably detect the expected effect size with an alpha rate of 0.05 in a standardly powered experiment and on the basis of extensive laboratory experience and literature in the field. There were further practical constraints related to the availability of samples and appropriate controls in the in vitro experiments, such as primary cell yield from animals and limitations in treatment or read-out methods, such as siRNA treatment or mass spectrometry. For human studies, the limiting factor was high-quality sample availability. These considerations collectively informed our sample size, which we deemed sufficient to observe biologically meaningful trends within the context of the aims of the study. Sample sizes are sufficient to detect significant changes on the basis of previous studies that used similar methodologies and experimental designs in the field. Data were formally excluded only if identified as a statistically significant outlier by Grubbs’s outlier test. Any outliers identified by Grubbs’s test were removed from the dataset, and the test was iterated until no outliers were detected. All replication attempts were successful. Each experiment was reproduced with similar results. Reproducibility is either indicated in the figure legends or shown as a quantification. For all experiments involving treatment with CSF1R or PLCi, animals or tissue culture wells were randomly assigned to groups. In other experiments, the mice were allocated into groups on the basis of either genotype or age. We did not compare human populations (control versus AD) but rather only within individuals with AD. The experimenters were blinded during the imaging and behavioural experiments. Gene expression and western blot analyses were not conducted under blinded conditions because they rely on quantitative read-outs, which lack subjectivity and are not influenced by knowledge of the experimental condition. Samples were processed either under blinded conditions or in batches.

### Reporting summary

Further information on research design is available in the Nature Portfolio Reporting Summary linked to this article.

## Online content

Any methods, additional references, Nature Portfolio reporting summaries, source data, extended data, supplementary information, acknowledgements, peer review information; details of author contributions and competing interests; and statements of data and code availability are available at 10.1038/s41586-025-09662-z.

## Supplementary information

Supplementary Information Supplementary Figs. 1–11 and Table 2.

Reporting Summary

Supplementary Tables Supplementary Table 1. Single-cell sequencing from wild-type and 5xFAD mice.Supplementary Table 2. Human demographics data. Supplementary Table 3. Single-nucleus sequencing from 5xFAD and 5xFAD^+^CSF1Ri microglia. Supplementary Table 4. MERFISH from 5xFAD–PU.1-low–wt–high microglia. Supplementary Table 5. TRAP sequencing from 5xFAD and 5xFAD^+^CSF1Ri microglia. Supplementary Table 6. TRAP sequencing from PU.1-low–wt–high microglia. Supplementary Table 7. ATAC sequencing from PU.1-low–wt–high microglia and T cells. Supplementary Table 8. Proteomics from PU.1-low–wt–high BV2 cells. Supplementary Table 9. RNA sequencing from PU.1-low iMgls. Supplementary Table 10. Single-nucleus sequencing from 5xFADPU.1-low–wt–high microglia. Supplementary Table 11. TRAP-seq from 5xFADPU.1-low–wt–high microglia. Supplementary Table 12. Single-cell sequencing from 5xFAD–CD28KO microglia. Supplementary Table 13. Single-nucleus sequencing from 5xFAD–TIM-3-KO microglia from Kimura et al.^49^.

## Source data

Source Data Fig. 1

Source Data Fig. 3

Source Data Fig. 4

Source Data Fig. 5

Source Data Extended Data Fig. 1

Source Data Extended Data Fig. 2

Source Data Extended Data Fig. 4

Source Data Extended Data Fig. 5

Source Data Extended Data Fig. 6

Source Data Extended Data Fig. 7

Source Data Extended Data Fig. 9

Source Data Extended Data Fig. 10

Source Data Extended Data Fig. 11

## Data Availability

All data have been made accessible in the indicated public repository. Sequencing and MERFISH data can be downloaded from the National Center for Biotechnology Information GEO: MERFISH (GSE275026), TRAP sequencing (GSE274896), single-cell sequencing (GSE296768), single-nucleus sequencing (GSE296523), iMgl sequencing (GSE296769) and ATAC-seq (GSE296641). The raw liquid chromatography–tandem mass spectrometry data used for proteomics have been deposited to the ProteomeXchange Consortium through the PRIDE partner repository, with the dataset identifier PXD063383. Upon request, the lead contacts are happy to provide any further information related to the data reported in this paper. Source data are provided with this paper.

## Extended data

Extended data is available for this paper at 10.1038/s41586-025-09662-z.

## Code availability

The code for MERFISH analysis is available at https://github.com/SchaferLabUMassChan/Ayata-et-al_2025. The code to analyse the human single-cell sequencing data of Olah et al.^15^ is available at https://github.com/tulsi92/pu1_paper_supp_fig7. The code to analyse the human single-cell sequencing data of Green et al.^17^ and AD Knowledge Portal can be found at https://github.com/maggieeiggam/pu1_paper/tree/main.
