# Supplementary material for: Lymphoid gene expression supports neuroprotective microglia function
Source: Nature. 2025 Nov 5;648(8092):157–65. doi: 10.1038/s41586-025-09662-z (PMC12675299; doi:10.1038/s41586-025-09662-z)
Supplement: Supplementary file 1 — Supplementary Figs. 1–11 and Table 2. [file 41586_2025_9662_MOESM1_ESM.pdf]

nature portfolio

https://doi.org/10.1038/s41586-025-09662-z

Supplementary information

Lymphoid gene expression supports neuroprotective microglia function

In the format provided by the authors and unedited

Supplementary Information

Lymphoid gene expression supports neuroprotective microglia function.

Authors

Pinar Ayata[1,2,3,18,*], Jessica M. Crowley[1,18], Matthew F. Challman[1,18], Vinaya Sahasrabuddhe[1,] Maud Gratuze[4,5], Sebastian Werneburg[6,7], Diogo Ribeiro[1], Emma C. Hays[1], Violeta Durán-Laforet[6], Travis E. Faust[6], Philip Hwang[1], Francisco Mendes Lopes[8], Chrysa Nikopoulou[8], Sarah Buchholz[8], Robert E. Murphy[6], Taoyu Mei[9], Anna A. Pimenova[1,2], Carmen Romero-Molina[1,2,10], Francesca Garretti[1,2,10], Tulsi A. Patel[1,2,10], Claudia De Sanctis[1,2,11], Angie V. Ramirez Jimenez[12], Megan Crow[13], Felix D. Weiss[14,15], Jason D. Ulrich[4], Edoardo Marcora[1,2,10], John W. Murray[16], Felix Meissner[14], Andreas Beyer[9] , Dan Hasson[12], John F. Crary[1,2,11], Dorothy P. Schafer[6], David M. Holtzman[4], Alison M. Goate[1,2,10], Alexander Tarakhovsky[17], Anne Schaefer[1,2,8]*

Affiliations

[1]Nash Family, Department of Neuroscience, Department of Psychiatry, Friedman Brain Institute, Icahn School of Medicine at Mount Sinai, New York, NY, USA.

[2]Ronald M. Loeb Center for Alzheimer's Disease, Center for Glial Biology, Icahn School of Medicine at Mount Sinai, New York, NY, USA.

[3]Neuroscience Initiative, Advanced Science Research Center, Graduate Program in Biology, Graduate Program in Biochemistry, Graduate Program in Neuroscience, The City University of New York Graduate Center, New York, NY, USA.

[4]Department of Neurology, Hope Center for Neurological Disorders, Charles F. and Joanne Knight Alzheimer's Disease Research Center, Washington University School of Medicine, St. Louis, MO, USA.

[5]Institute of Neurophysiopathology (INP), University of Aix-Marseille, CNRS UMR 7051, 13385 Marseille, France.

[6]Department of Neurobiology, Brudnick Neuropsychiatric Research Institute, University of Massachusetts Chan Medical School, Worcester, MA, USA.

[7]Department of Ophthalmology and Visual Sciences, Kellogg Eye Center, Michigan Neuroscience Institute, Department of Molecular & Integrative Physiology, University of Michigan, Michigan Medicine, MI, USA.

[8]Max Planck Institute for Biology of Ageing, Cologne Excellence Cluster for Aging and Aging-Associated Diseases (CECAD), University of Cologne, Cologne, Germany.

[9]Cologne Excellence Cluster for Aging and Aging-Associated Diseases (CECAD), Center for Molecular Medicine Cologne, Faculty of Medicine and University Hospital Cologne, Institute for Genetics, Faculty of Mathematics and Natural Sciences, University of Cologne, Cologne, Germany.

[10]Department of Genetics & Genomic Sciences, Icahn School of Medicine at Mount Sinai, New York, NY, USA.

[11]Department of Pathology, Department of Artificial Intelligence & Human Health, Neuropathology Brain Bank and Research CoRE, Icahn School of Medicine at Mount Sinai, New York City, New York, USA.

[12]Bioinformatics for Next-Generation Sequencing (BiNGS) Core, Tisch Cancer Institute, Department of Oncological Sciences, Icahn School of Medicine at Mount Sinai, New York, New York 10029, USA.

[13]Department of Human Genetics, Genentech Inc., South San Francisco, CA, USA

[14]Institute of Innate Immunity, Department for Systems Immunology and Proteomics, Medical Faculty, University of Bonn, Bonn, Germany

[15]German Rheumatology Research Center (DRFZ), A Leibniz Institute, Berlin, Germany

[16]Columbia Center for Human Development, Center for Stem Cell Therapies, Department of Medicine, Columbia University Vagelos College of Physicians and Surgeons, New York, NY 10032, USA.

[17]Laboratory of Immune Cell Epigenetics and Signaling, The Rockefeller University, New York, NY, USA.

[18]Authors contributed equally to this work

* Corresponding authors

Supplementary Figure 1. Identification of a microglia subpopulation with low PU.1 expression at the plaque, related to Figure 1.

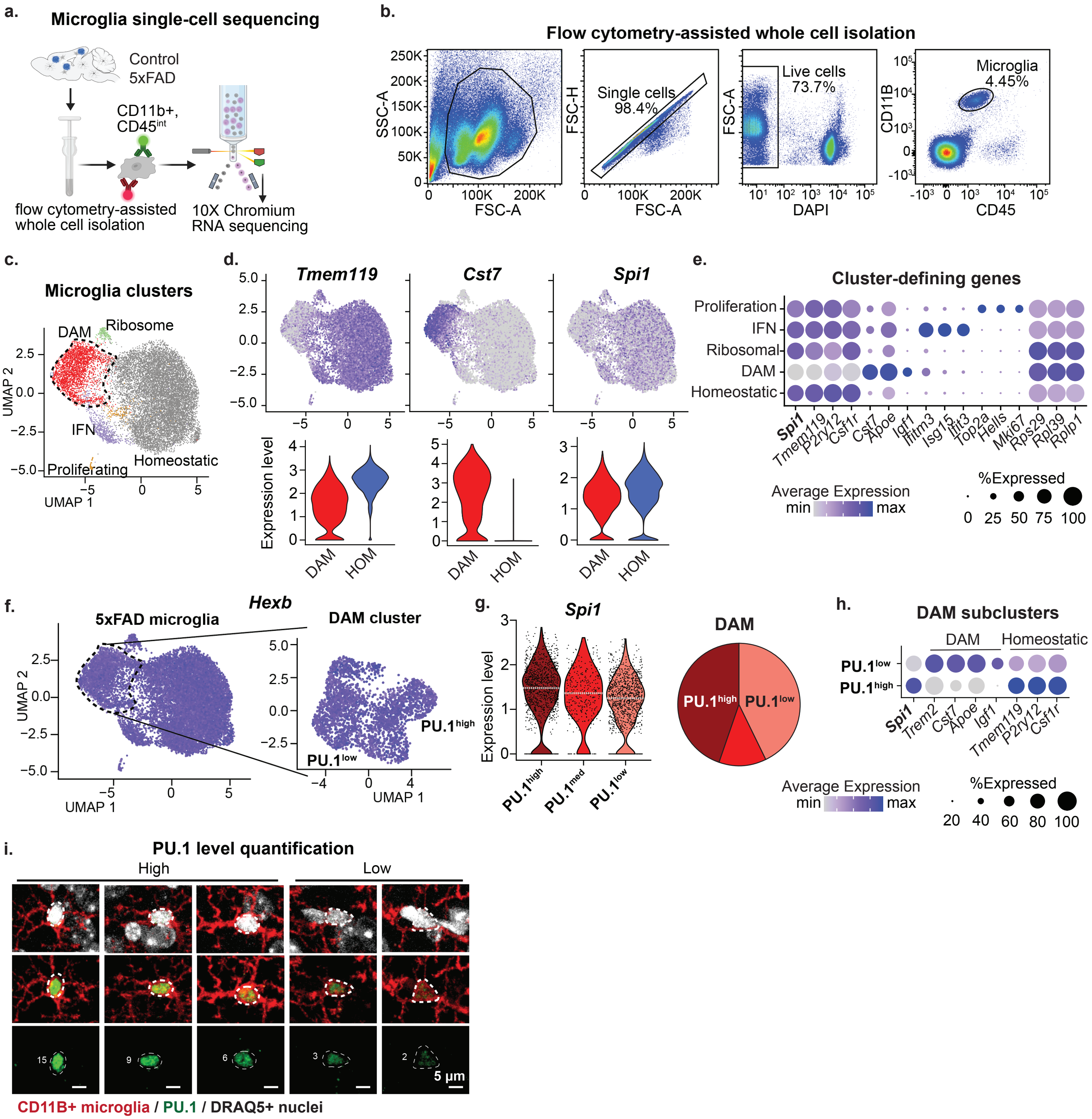

Supplementary Figure 1. Identification of a microglia subpopulation with low PU.1 expression at the plaque, related to Figure 1. (a) Schematic shows the isolation of microglia by fluorescence-activated cell sorting (FACS) followed by single-cell sequencing from forebrain microglia. **(b)** Microglia isolation: Scatter plots show representative FACS plots generated on the BD FACS Aria and visualized with FlowJo v10 and the gating strategy to isolate CD45^{int} CD11B+ microglia. Each plot shows the percentage of the target population from the parent gate. Cells were gated based on size and granularity using FSC-A vs. SSC-A to eliminate debris. Single cells were additionally gated using FSC-H (shown) and SSC-W (not shown, 98.9% of the parent gate). Live cells were gated using DAPI to mark dead cells. Microglia were gated for intermediate CD45 and high CD11B expression. SSC-A: side scatter area, FSC-A: forward scatter area, FSC-H: forward scatter height. SSC-W: side scatter width. **(c-g)** Microglia single-cell sequencing analysis: Single-cell sequencing from forebrain microglia of 8-month-old wild-type (2♂) and 5xFAD mice (2♂). IFN: Interferon-responsive microglia. DAM: disease-associated microglia. **(c)** Uniform manifold approximation and projection (UMAP) visualizations

show microglia automatically assigned into clusters that are manually annotated based on the marker genes shown in Supplementary Table 1. **(d)** Feature plots in UMAP visualizations (top) and violin plots (bottom) show the expression of indicated genes by microglia. **(e)** The dot plot shows the scaled average expression per cluster (color) and the percentage of cells (size) from each cluster of microglia. **(f)** Feature plots show the expression of *Hexb* in UMAP visualizations of all microglia (left) and the DAM subset after reclustering (right). **(g)** Left: Violin plots show *Spi1* expression level in the three DAM subclusters. Gray dotted lines represent the mean. Right: The pie charts show the percentage of PU.1^{low} DAM subcluster. **(h)** The dot plot shows the scaled average expression levels per cluster (color) and the percentage of cells (size) from DAM subclusters. **(i)** PU.1 expression analysis: A representative immunofluorescence image for PU.1 expression in microglial nuclei identified by CellProfiler with the corresponding PU.1 intensity. CD11B+ microglia: red, PU.1: green, DRAQ5+ nuclei: gray. Scale bar: 5 μm. Microglial nuclei are indicated in dotted circles. Independently repeated >20 times.

Supplementary Figure 2. Low PU.1 expression is associated with CSF1R-independent microglia survival at the plaque, related to Figure 1.

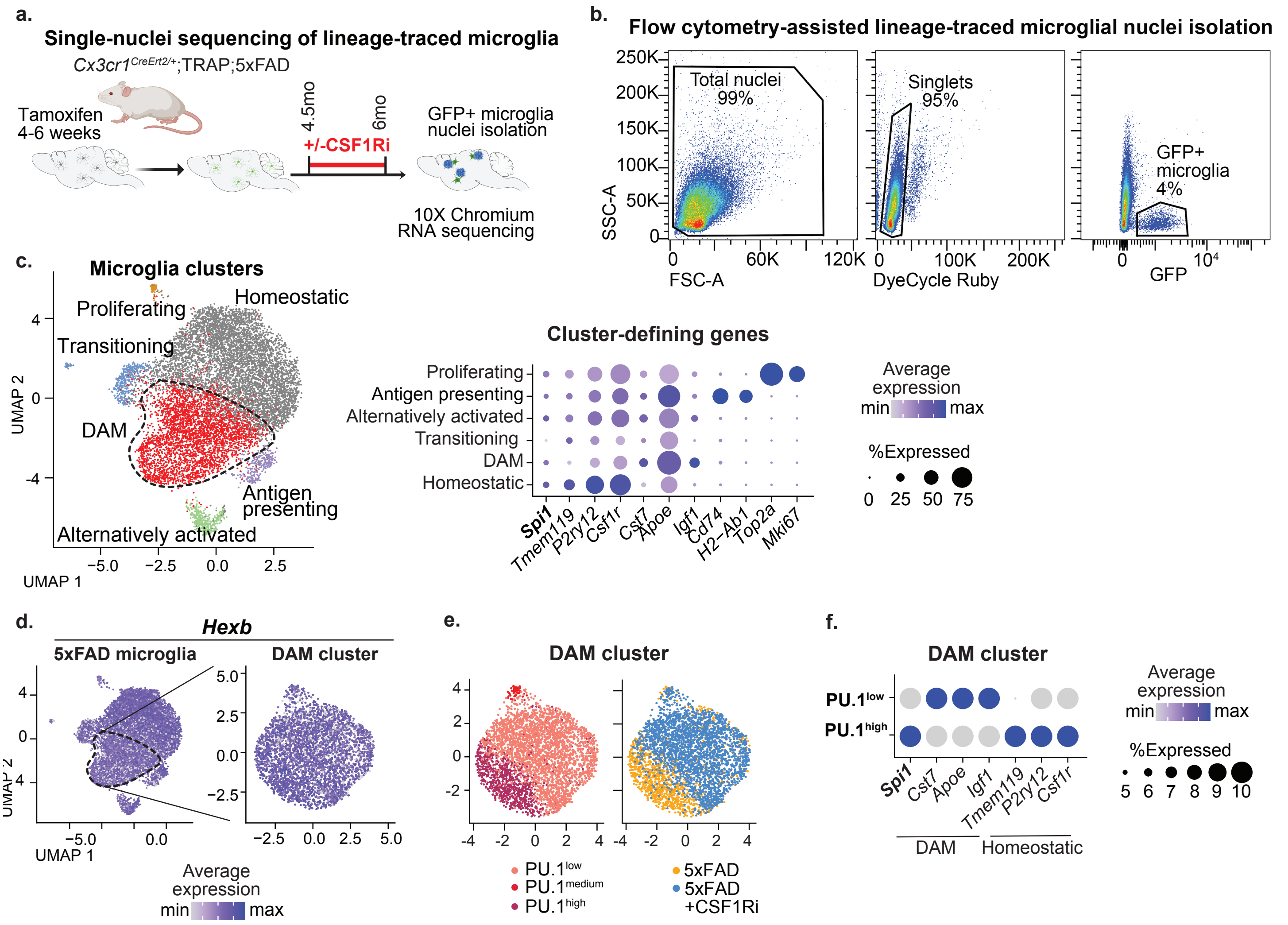

Supplementary Figure 2. Low PU.1 expression is associated with CSF1R-independent microglia survival at the plaque, related to Figure 1. (a-f) PU.1^{low} plaque-associated microglia show CSF1R-independent survival. (a) The schematic shows GFP+ labeling of microglial nuclei in 5xFAD mice crossed to microglia-specific TRAP mice by tamoxifen administration at 4-6 weeks, 6 weeks of CSF1Ri or control treatment starting at 4.5 months old, the isolation of lineage-traced GFP+ microglial nuclei by fluorescence-activated nuclear sorting (FANS) at 6 months, and single-nuclei sequencing by 10x Chromium. **(b)** Microglia nuclei isolation: Scatter plots show representative FANS plots generated on the BD FACS Aria and visualized with FlowJo v10 and the gating strategy to isolate GFP+ microglial nuclei. Each plot shows the percentage of the target population from the parent gate. Nuclei were gated based on size and granularity using FSC-A vs SSC-A to eliminate debris. Single nuclei (singlets) were gated using DyeCycle Ruby to label DNA. Microglial nuclei were gated for eGFP expression (based on nucleolar localization of eGFP-RPL10A-labeled ribosomes). SSC-A: side scatter area, FSC-A: forward scatter area. **(c-f)** Microglia single nuclei sequencing analysis: Single-nuclei sequencing of cortical lineage-traced microglia from 6-month-old control diet- (1♀) and CSF1Ri diet-fed (2♂ pooled) 5xFAD mice. DAM: disease-associated microglia. **(c)** Left: Uniform manifold approximation and projection (UMAP) visualizations show microglia automatically assigned into clusters that are manually annotated based on the marker genes shown in Supplementary Table 3. Right: The dot plot shows the scaled average expression levels per cluster (color) and the percentage of cells (size) from each microglia cluster. **(d)** Feature plots show the expression of microglia-specific *Hexb* in UMAP visualizations of all cortical microglia (top) and the reclustered DAM subset (bottom). **(e)** UMAP visualizations show automatic subclustering of DAM with subclusters annotated as PU.1^{low}, PU.1^{medium}, and PU.1^{high} based on *Spi1* (PU.1) expression (left) and split by diet (right). **(f)** The dot plot shows the scaled average expression (color) of selected genes in PU.1^{low} and PU.1^{high} DAM subclusters and the percentage of cells (size) expressing the genes.

Supplementary Figure 3. Identification of brain cell types and plaque-associated microglia by MERFISH analysis, related to Figure 2.

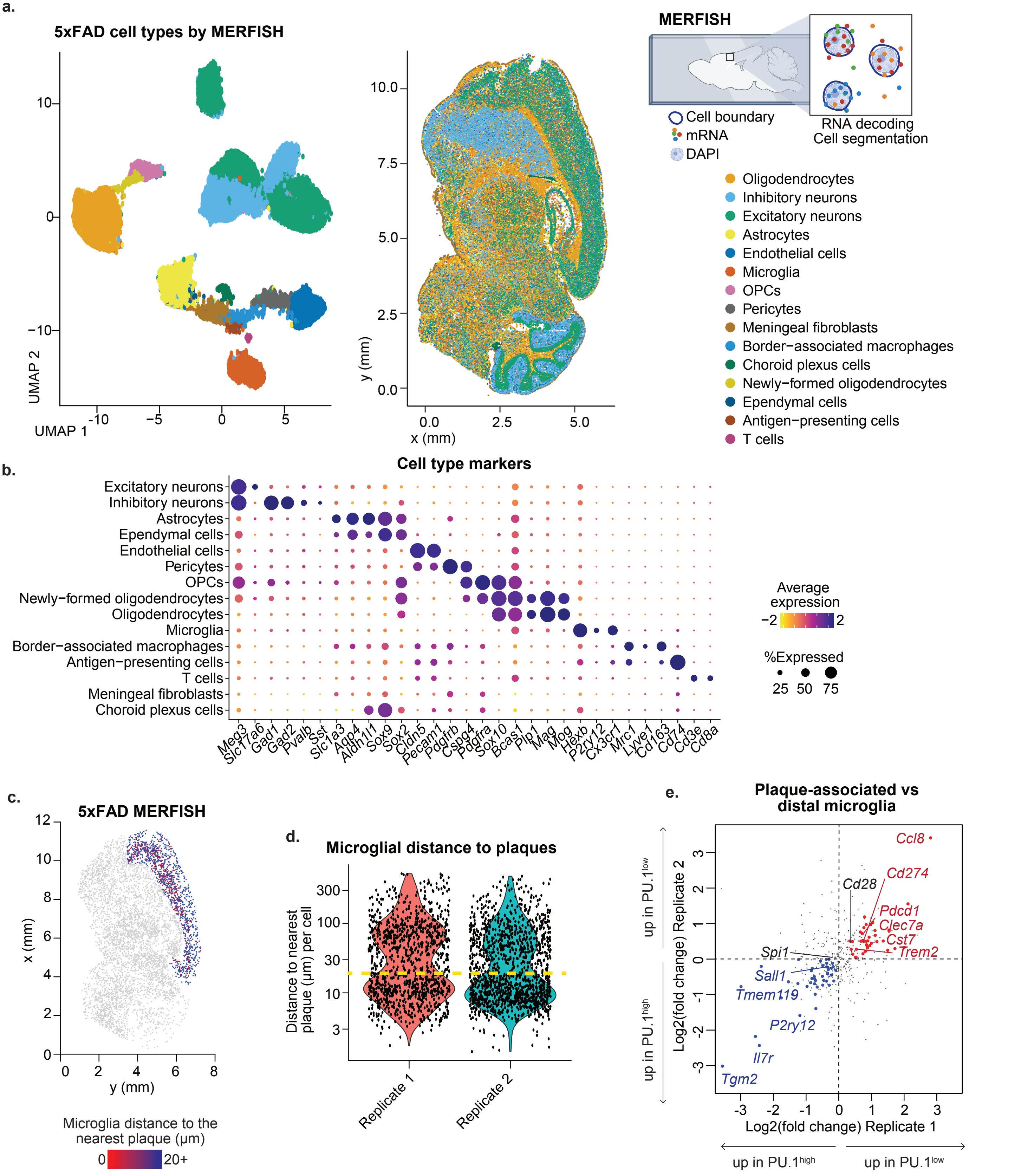

Supplementary Figure 3. Identification of brain cell types and plaque-associated microglia by MERFISH analysis, related to Figure 2. (a-e) Multiplexed error-robust fluorescent in situ hybridization (MERFISH) within the brain of the 5xFAD model (8-month-old, ♂). OPCs: Oligodendrocyte progenitor cells. **(a)** Uniform manifold approximation and projection (UMAP) visualization (left) and representative spatial distribution in one 5xFAD mouse (middle), and schematic (right) show all cell types identified from automated clusters and manually

annotated. Marker genes are shown in Supplementary Table 4. **(b)** The dot plot shows the average scaled expression per cluster (color) and the percentage of cells (size) from each cell type identified. **(c)** The representative spatial distribution of microglia within the cortex of one 5xFAD mouse. The distance of each microglia to the nearest plaque is shown (color). **(d)** Violin plot with individual data points shows the distance of each microglia in two ♂ 5xFAD mice to the nearest plaque. The dotted yellow line indicates the cut-off used to differentiate between plaque-associated and distal microglia (15 μm). We used a larger distance cutoff for nuclei identified in MERFISH than cells identified by immunofluorescence to account for the soma and processes. Please see Fig. 2c. **(e)** The scatter plot shows a comparison of log2 (fold change) of significantly upregulated genes in the plaque-associated (red) vs. distal microglia (blue) in the cortex between two independent replicates—Wilcoxon rank sum test.

Supplementary Figure 4. Plaque-associated, PU.1low microglia display ribosome association of lymphoid-expressed genes, related to Figure 2.

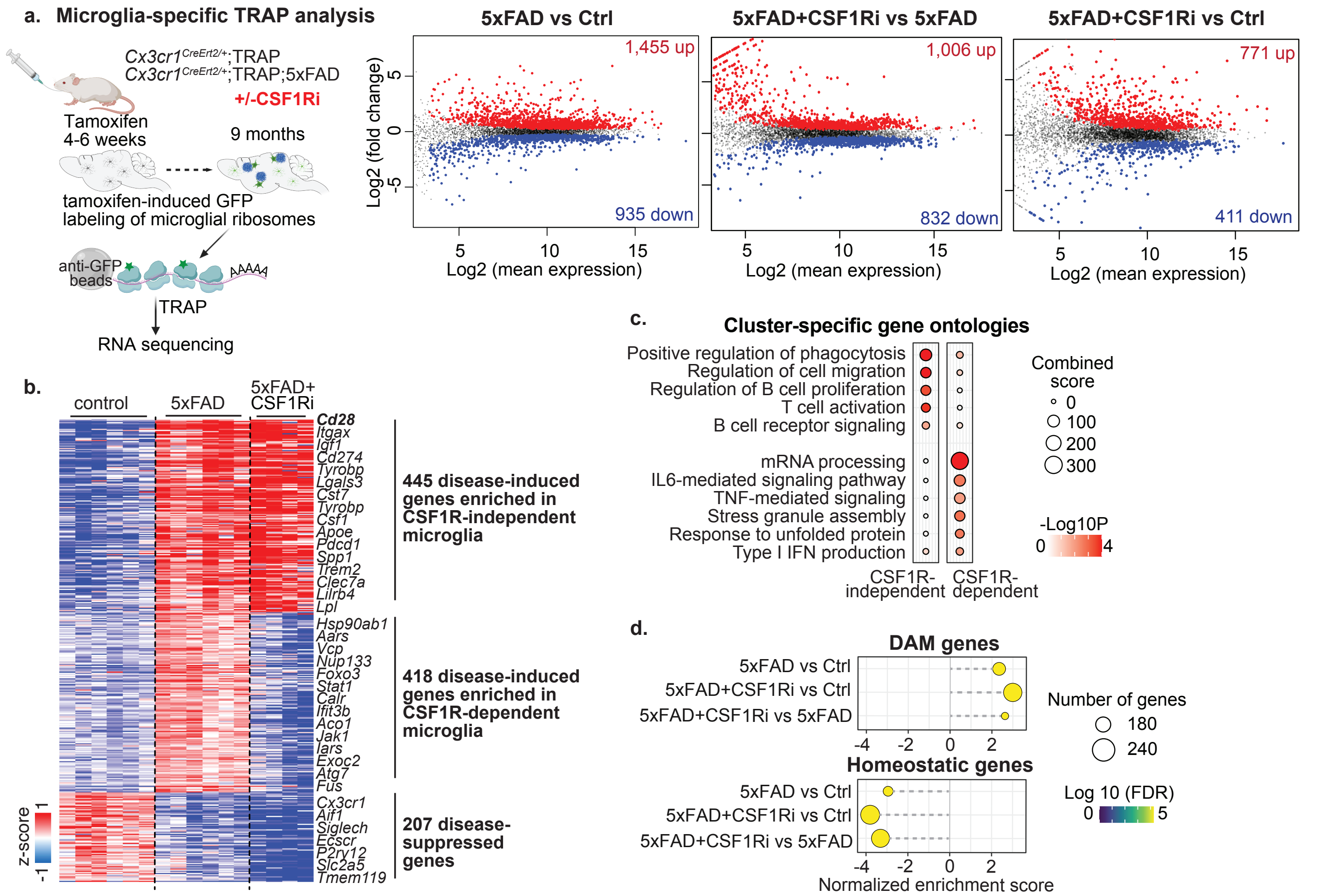

Supplementary Figure 4. Plaque-associated, PU.1^{low} microglia display ribosome association of lymphoid-expressed genes, related to Figure 2. (a-d) Microglia-specific translating ribosome affinity purification (TRAP) sequencing from 6-month-old control (n=6 mice, 3♀, 3♂), 5xFAD (n=6 mice, 3♀, 3♂), and CSF1Ri-treated 5xFAD mice (n=4 mice, 2♀, 2♂). **(a)** Schematic showing experimental design (left) and MA plots (right, representing log-ratio (M) on the y-axis and mean average (A) on the x-axis) show gene expression changes (red, upregulated; blue, downregulated by DESeq2) shown in Supplementary Table 5. **(b)** The CSF1R-resistant microglia show expression of DAM and lymphoid-expressed genes, while CSF1R-dependent microglia express inflammatory and cellular stress response genes. Heatmap shows z-scored variance stabilizing transformations (vsts, color) of genes selected by overlapping and non-overlapping genes between pairwise comparisons (5xFAD vs. control and 5xFAD+CSF1Ri vs. control). **(c)** The balloon plot shows differentially regulated gene ontologies (EnrichR) associated with 418 CSF1Ri-dependent and 445 CSF1Ri-independent microglia-expressed and disease-induced genes in 5xFAD mice, as shown in (b). p-value (color, Fisher's exact test) and combined score (size) are shown. **(d)** The balloon plot represents gene set enrichment analysis (GSEA) gene enrichment analysis of indicated gene sets for disease-associated microglia (DAM) genes identified by *Keren-Shaul et al.*[12]. The normalized enrichment score (x-axis), the false discovery rate (FDR, color, modified Kolmogorov-Smirnov test), and the number of genes (size) are shown.

Supplementary Figure 5. PU.1^{low} lymphoid gene-expressing microglia are present in patients with AD, related to Figure 2.

a.

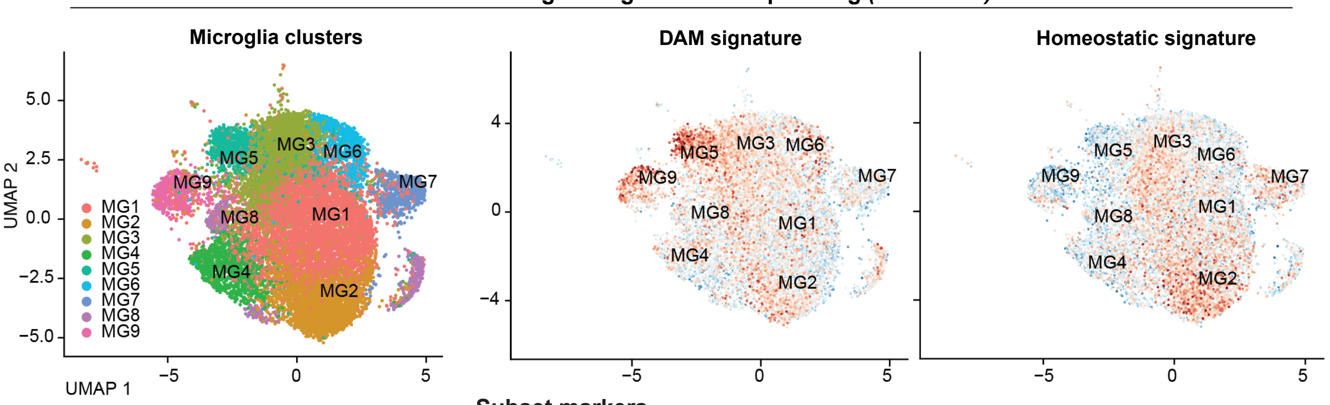

b.

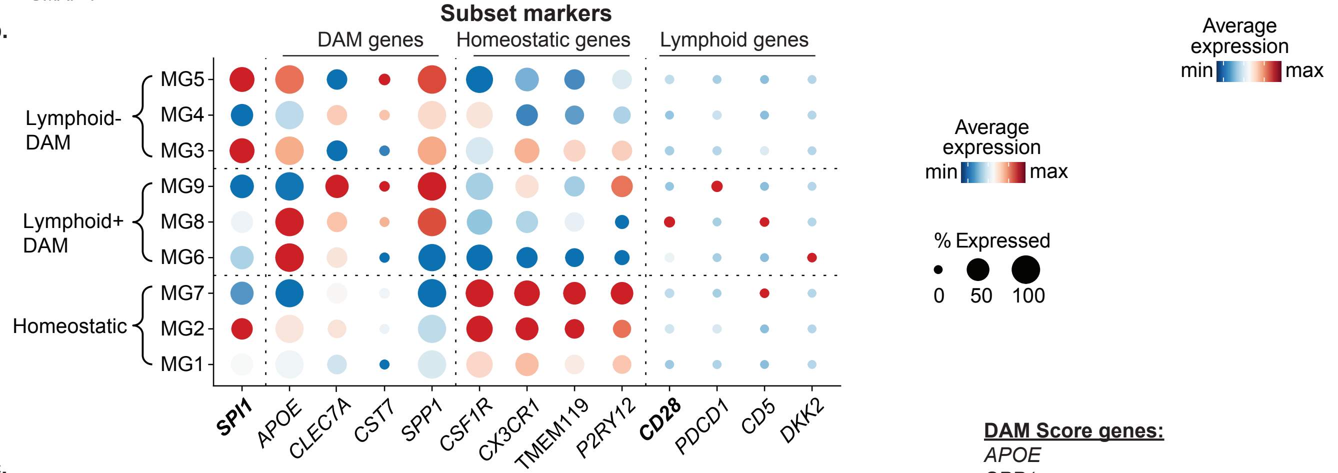

c.

		DAM Score	Homeostatic Score	DAM/Homeostatic Ratio	Lymphoid Score
Lymphoid- DAM	**MG5**	0.7916	0.1884	4.20	-0.00031
	**MG4**	0.5406	0.2694	2.01	-0.00056
	**MG3**	0.6101	0.3917	1.56	-0.00024
Lymphoid+ DAM	**MG9**	0.7497	0.1544	4.86	0.00501
	**MG8**	0.5621	0.2187	2.57	0.00361
	**MG6**	0.5893	0.2451	2.40	0.00085
Homeostatic	**MG7**	0.5162	0.3876	1.33	0.00003
	**MG2**	0.5440	0.4649	1.17	0.00018
	**MG1**	0.5523	0.3607	1.53	-0.00056

DAM Score genes:
APOE
SPP1
CST7
CLEC7A

Homeostatic Score genes:
CSF1R
CX3CR1
TMEM119
P2RY12

Lymphoid Score genes:
CD28
PDCD1
CD5
DKK2

Scaled values
min max

d.

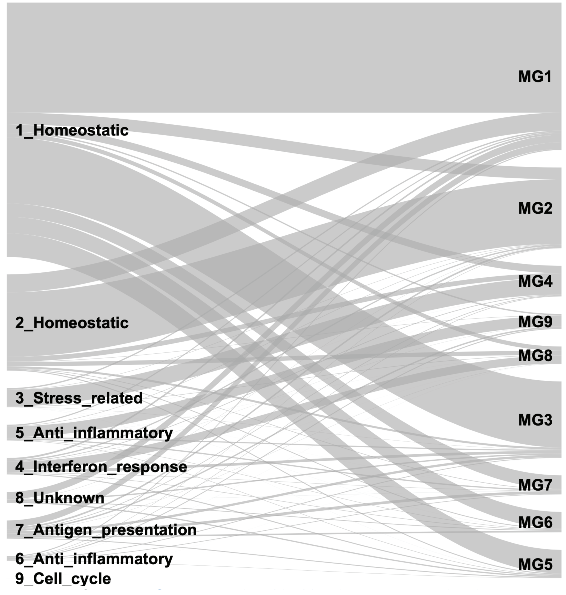

e.

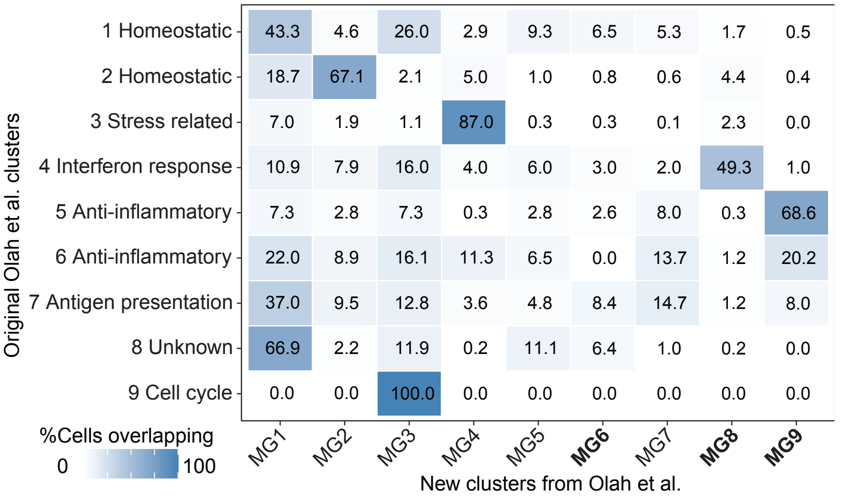

Supplementary Figure 5. PU.1^{low} lymphoid gene-expressing microglia are present in patients with AD, related to Figure 2. (a-e) Reanalysis of human microglia single-nuclei sequencing data from *Olah et al.*[15] **(a)** Left: Uniform manifold approximation and projection (UMAP) visualizations of single-nuclei sequencing data assigned into clusters. Right: Feature plots show the expression of DAM (*APOE, SPP1, CLEC7A, CST7*) and homeostatic signature genes (*CSF1R, CX3CR1, TMEM119, P2RY12)* in UMAP visualizations. **(b)** The dot plot shows the scaled average expression levels per cluster (color) and the percentage of cells (size) from each previously identified cluster. **(c)** Heatmap shows the expression of DAM (*APOE, SPP1, CLEC7A, CST7*), homeostatic (*CSF1R, CX3CR1, TMEM119, P2RY12),* and lymphoid signature genes (*CD28, PDCD1, CD5, DKK2)* per cluster. Colors are scaled per column and represent the numbers shown. Microglia clusters are organized into three new, larger clusters: Homeostatic, Lymphoid+ DAM, and Lymphoid -DAM based on signature DAM/Homeostatic ratio and Lymphoid score. **(d)** Sankey plot depicting the mapping of cells from the original clusters (left) to newly reprocessed clusters (right). Each flow represents the proportion of cells from a given original cluster reassigned to one of the newly defined clusters (MG1–MG9). The width of each flow is proportional to the number of cells transitioning between clusters, illustrating both conserved and redistributed cell identities upon reanalysis. **(e)** Confusion matrix heatmap quantifying the percentage overlap between original and new clusters. Each value represents the percentage of cells from an original cluster (rows) that map to each new cluster (columns). Percentages are shown in blue, with higher values indicating greater concordance. The matrix provides a quantitative summary of cluster correspondence and divergence resulting from reprocessing.

Supplementary Figure 6. PU.1 expression levels govern microglial chromatin accessibility patterns, related to Figure 3.

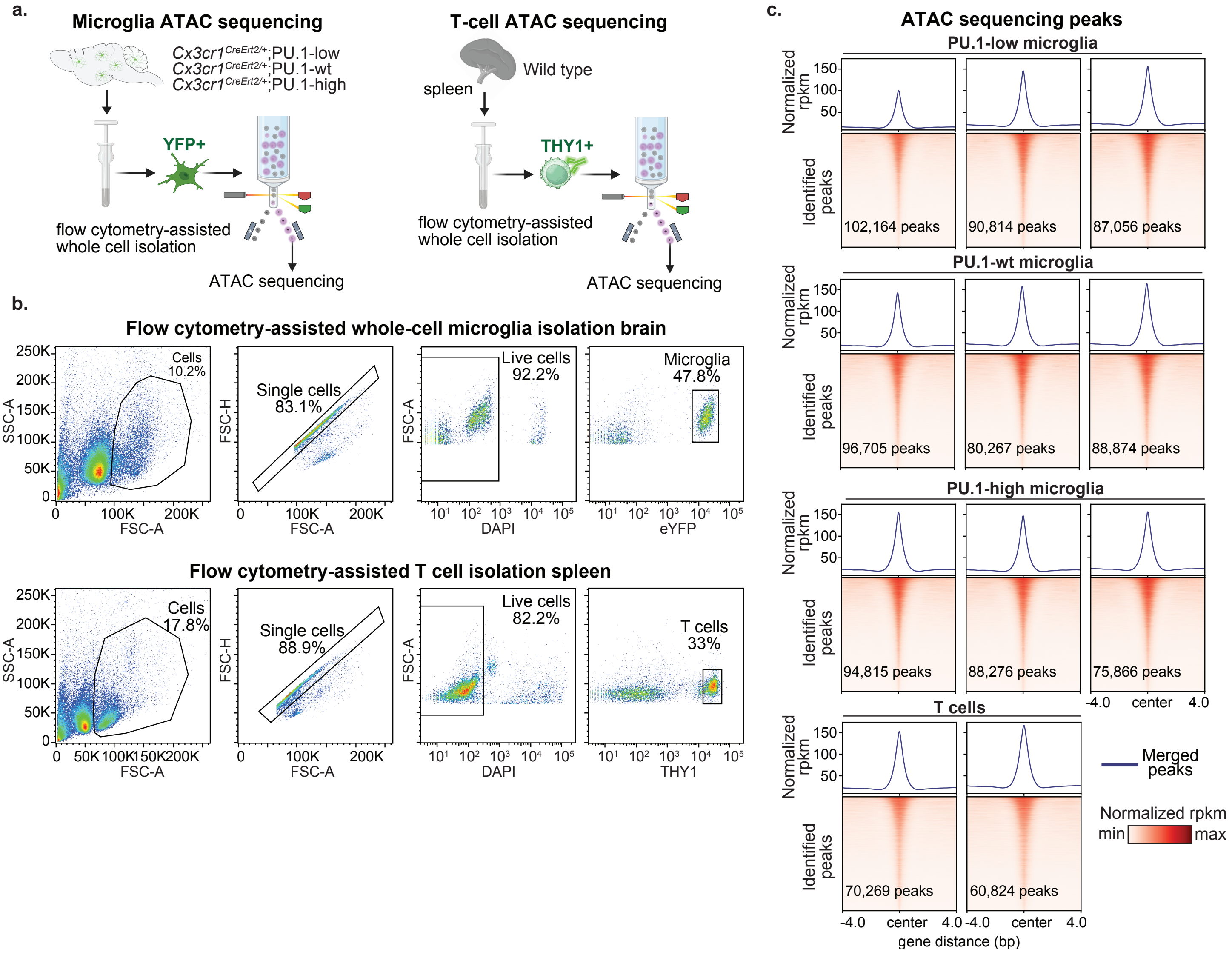

Supplementary Figure 6. PU.1 expression levels govern microglial chromatin accessibility patterns, related to Figure 3. (a) Schematics show the isolation of PU.1-low, PU.1-wild-type (PU.1-wt), and PU.1-high microglia from the brain (left) and wild-type T cells from the spleen (right) by fluorescence-activated cell sorting (FACS) followed by ATAC sequencing. **(b)** Scatter plots show representative FACS plots generated on the BD FACS Aria and visualized with FlowJo v10, using a gating strategy to isolate YFP+ microglia and THY1+ T cells. Each plot shows the percentage of the target population from the parent gate. Cells were gated based on size and granularity using FSC-A vs SSC-A to eliminate debris and clumped cells. Single cells were additionally gated using FSC-H (shown) and SSC-W (not shown, 99.1% and 98.4% of the parent gate in microglia and T cells, respectively). Live cells were gated using DAPI to exclude dead cells. Microglia were gated for YFP expression. T cells were gated for THY1 expression. Values inside the plots represent the percentages from the parent gate (Live cells). SSC-A: side scatter area, FSC-A: forward scatter area, FSC-H: forward scatter height. SSC-W: side scatter width. **(c)** ATAC sequencing of microglia from PU.1-low, PU.1-wt, and PU.1-high microglia from the brain (n=3 mice/group) and T-cells from the spleen (n=2 mice). 9- to 10-month-old ♂ mice were used. 50,000 cells used per sample. The profile plots (top) and heatmaps (bottom) show normalized rpkm signals**.** Data are centered on +4Kb around called peaks in each sample. Peaks are ranked by signal intensity with the same minimum and maximum range for the profile plot and heatmap.

Supplementary Figure 7. PU.1 levels govern microglia transcriptional responses to amyloid pathology in 5xFAD mice: MERFISH analysis, related to Figure 4.

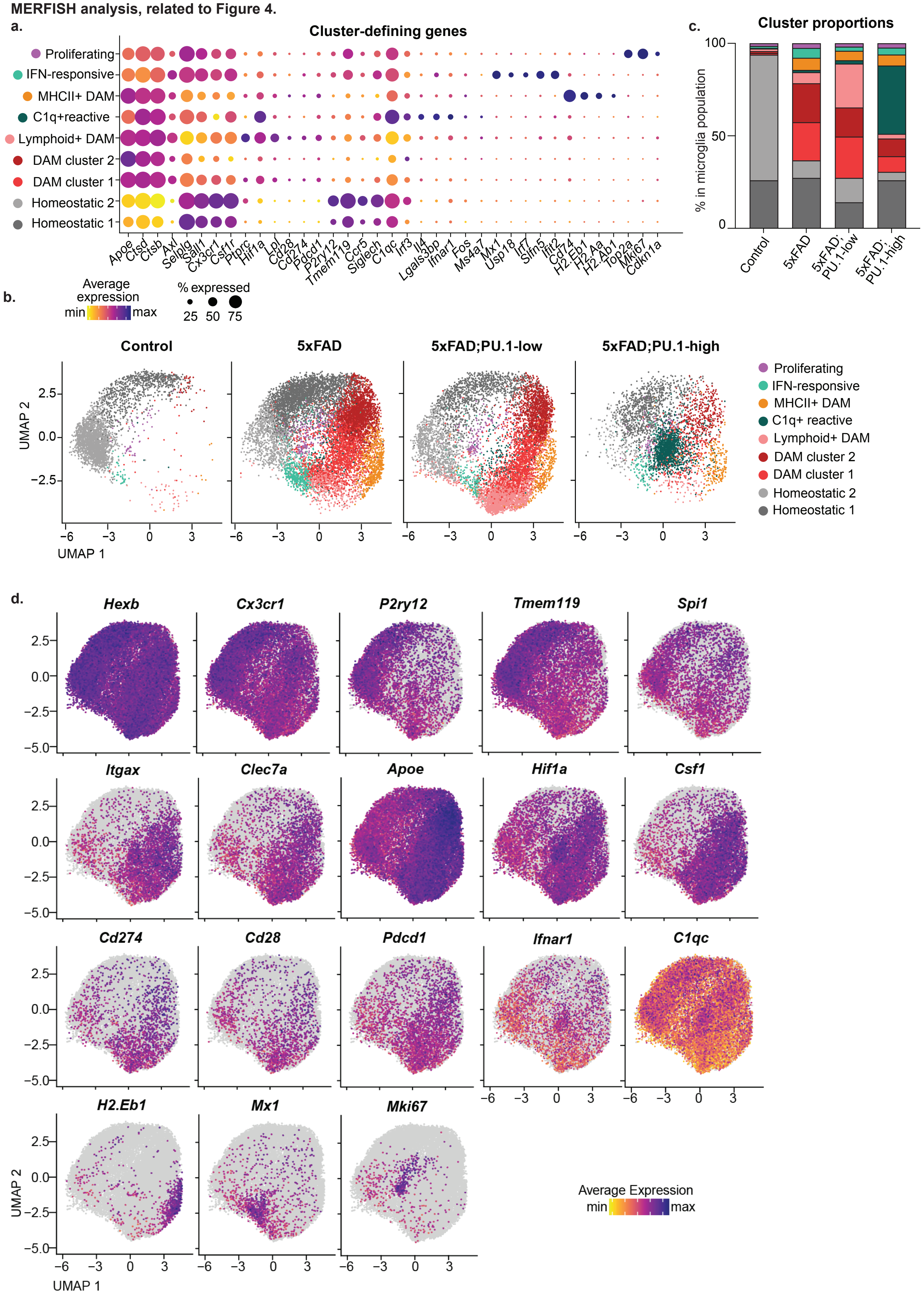

Supplementary Figure 7. PU.1 levels govern microglia transcriptional responses to amyloid pathology in 5xFAD mice: MERFISH analysis, related to Figure 4. (a-d) Multiplexed error-robust fluorescent in situ hybridization (MERFISH) in 8-month-old wild-type (n=2 ♂ mice), 5xFAD (n=3 ♂ mice), 5xFAD-PU.1-low (n=2 ♂ mice), and 5xFAD-PU.1-high mice (n=1♂ mouse). Please also see Supplementary Fig. 3. **(a)** The dot plot shows the scaled average expression of cluster markers (shown in Supplementary Table 4) per cluster (color) and the percentage of cells (size) from each microglia cluster identified by automated clustering with manually annotated populations indicated. **(b)** Uniform manifold approximation and projection (UMAP) visualizations show microglia assigned into automated clusters with manually annotated populations indicated. **(c)** The bar graphs show the change in the percentage of populations in different genotypes. **(d)** Feature plots show the expression of indicated genes in UMAP visualizations of microglia.

Supplementary Figure 8. CD28 suppresses broad interferon response in microglia, related to Figure 5.

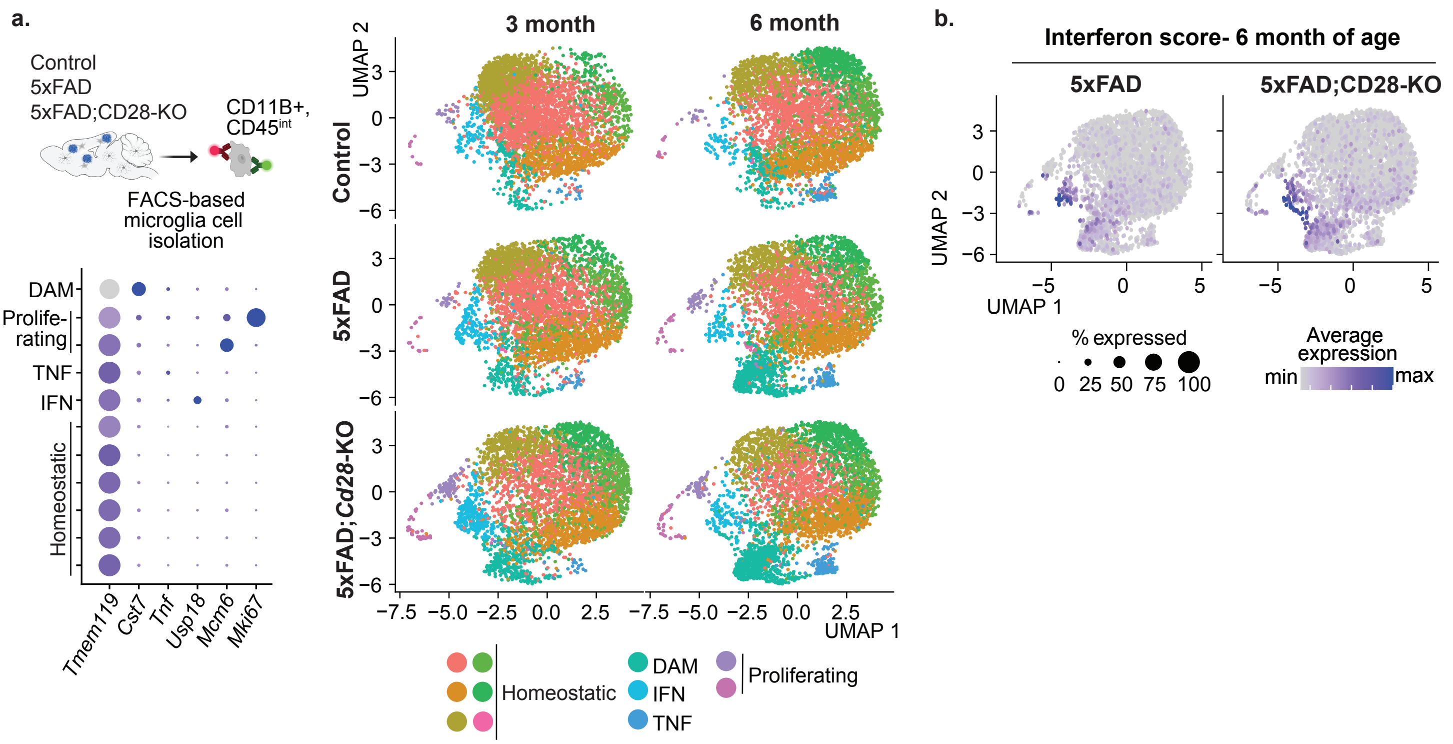

Supplementary Figure 8. CD28 suppresses broad interferon response in microglia, related to Figure 5. (a-b) CD28 regulates inflammatory microglia responses in 5xFAD mice. Single-cell sequencing of CD45^{low} CD11B+ forebrain microglia from 3- and 6-month-old wild-type, 5xFAD, and 5xFAD;CD28-KO mice (control: n=1 at 3 and 6 months, 5xFAD and 5xFAD;CD28-KO: n=2 mice at 3 months, 5xFAD and 5xFAD;CD28-KO: n=1 at 6 months, all ♂). HOM: Homeostatic. Prolif: Proliferating. TNF: TNF-expressing microglia. IFN: Interferon-responsive microglia. DAM: disease-associated microglia. **(a)** Top left: Schematic showing microglia isolation for single-cell sequencing. Right: Uniform manifold approximation and projection (UMAP) visualizations (left) of microglia assigned into clusters. Disease-associated microglia (DAM) population is indicated with a dotted line. Bottom left: The dot plot shows the scaled average expression of cluster markers per cluster (color) and the percentage of cells (size) shown in Supplementary Table 12. **(b)** Feature plots show the expression of interferon genes (*Ifitm3, Stat1, Ifit3, Isg15, Ifit2, Ifi27l2a, Ifi204, Irf7, Usp18, Stat2, Ifitm2, Rsad2, Isg15, Ifit1, Bst2, Isg20,* and *Xaf1*) in UMAP visualizations of single-cell sequencing of microglia from 6-month-old mice.

Supplementary Figure 9. Original Western blots 1.

Extended Data Figure 4b

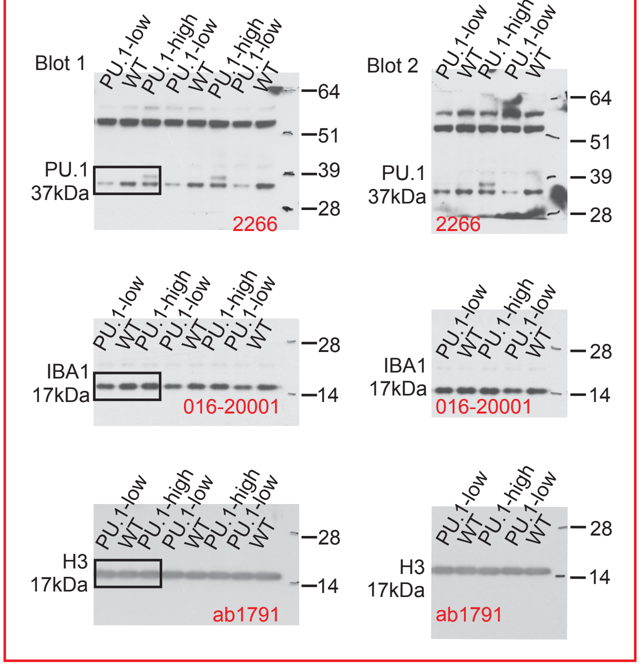

Extended Data Figure 5a

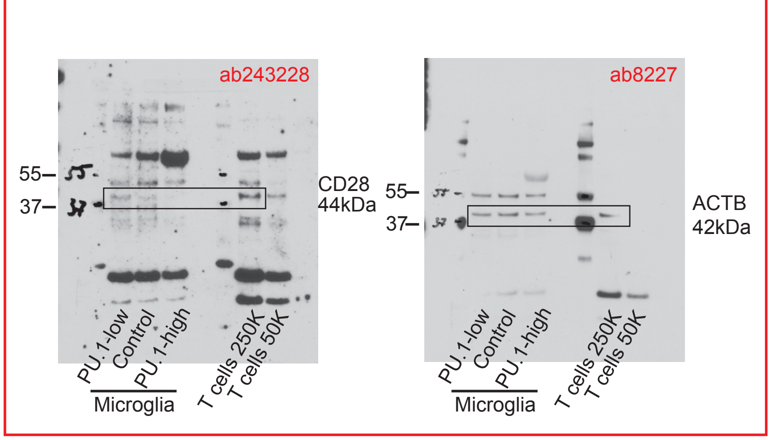

Extended Data Figure 5b

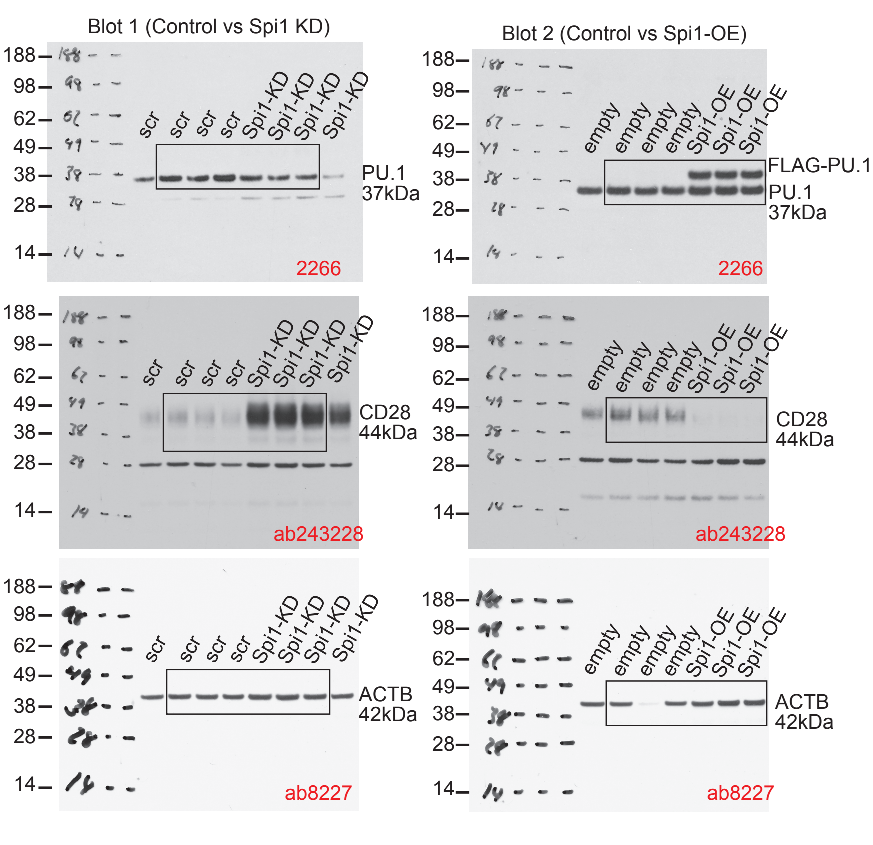

Supplementary Figure 9. Original Western blots 1. All samples were run on a 4-12% NuPAGE Bis-Tris gel and exposed on KODAK film and scanned unless otherwise specified. Some membranes were cut prior to antibody staining to allow for the simultaneous detection of proteins running at different sizes on the same membrane. Representative blots, as shown in the indicated figures, are boxed.

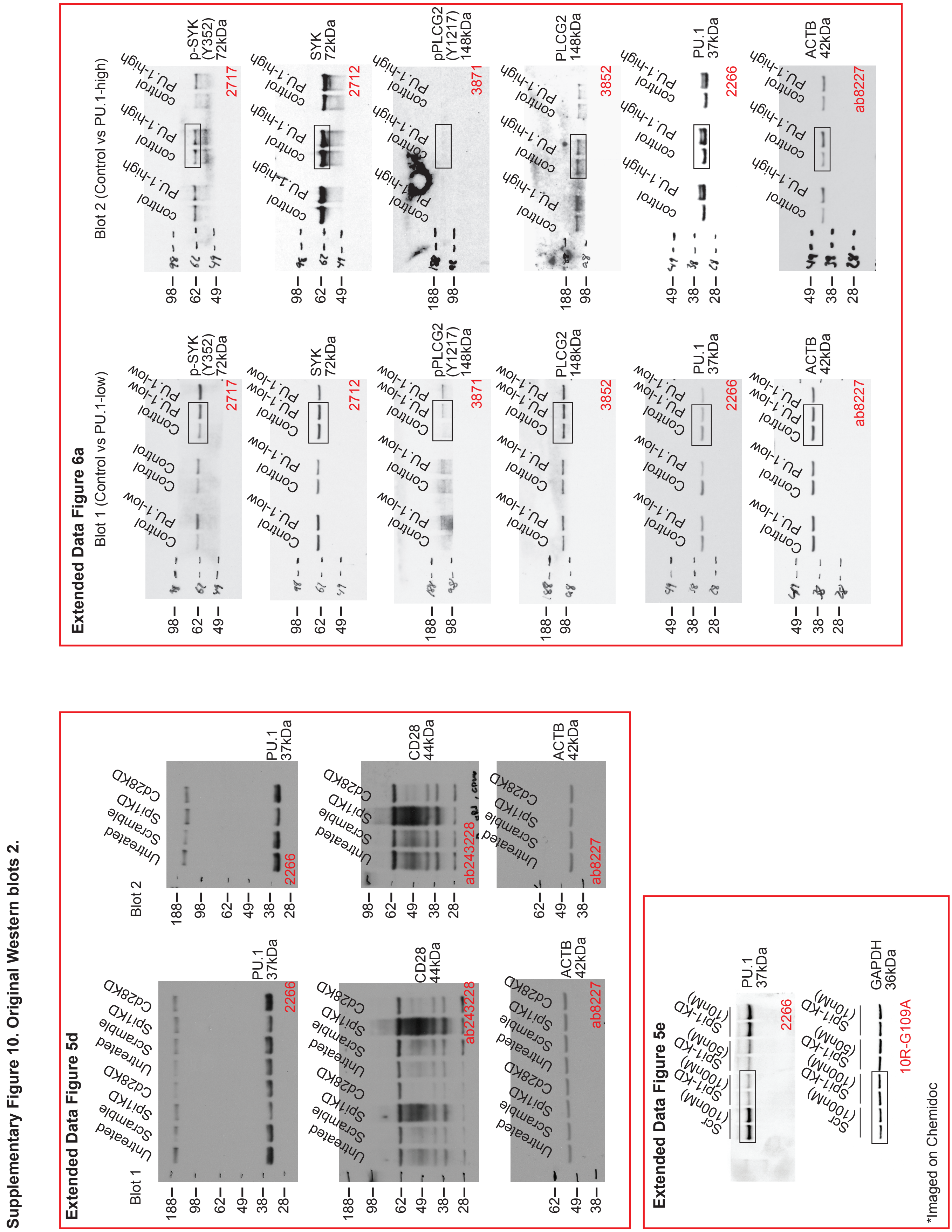

Supplementary Figure 10. Original Western blots 2. All samples were run on a 4-12% NuPAGE Bis-Tris gel and exposed on KODAK film and scanned unless otherwise specified. Some membranes were cut prior to antibody staining to allow for the simultaneous detection of proteins running at different sizes on the same membrane. Representative blots, as shown in the indicated figures, are boxed.

Supplementary Figure 11. Original Western blots 3.

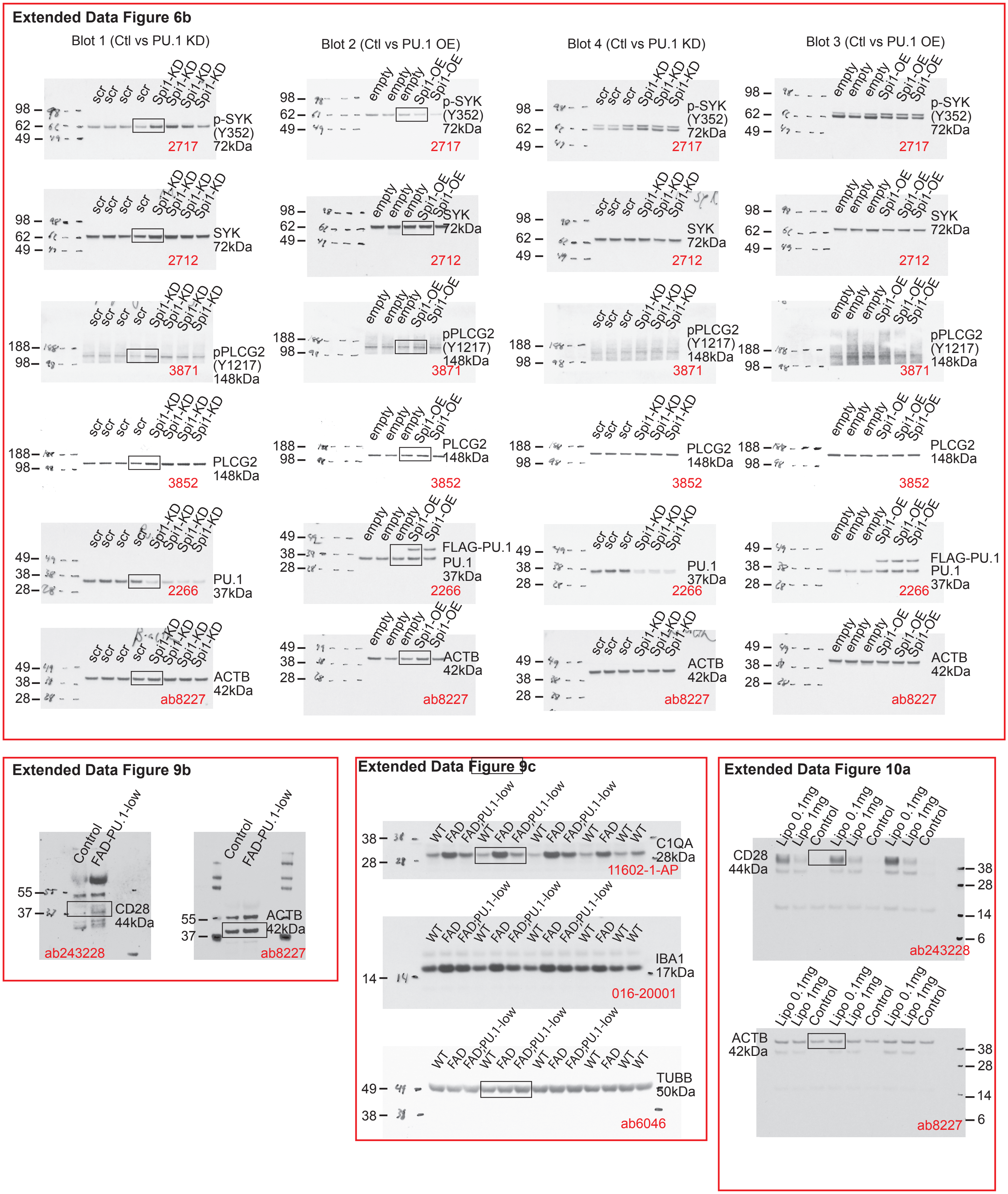

Supplementary Figure 11. Original Western blots 3. All samples were run on a 4-12% NuPAGE Bis-Tris gel and exposed on KODAK film and scanned unless otherwise specified. Some membranes were cut prior to antibody staining to allow for the simultaneous detection of proteins running at different sizes on the same membrane. Representative blots, as shown in the indicated figures, are boxed.

Case	Figure	Age	Sex	Clincial diagnosis	Clinical cognitive status	Braak stage	ADNC	Primary neuropathological diagnosis
**1**	**1c**	85	M	HFrEF	None	Stage III (B2)	Intermediate	Alzheimer's disease neuropathologic change (ADNC), intermediate (A3B2C1)
**2**	**1c**	91	F	AD/ dementia	Dementia	Stage VI (B3)	Intermediate	Alzheimer's disease neuropathologic change (ADNC), intermediate (A2B3C3)
**3**	**1c**	79	M	Advanced dementia	Dementia	Stage IV (B2)	Intermediate	Alzheimer's disease neuropathologic change (ADNC), intermediate (A2B2C1) & cerebrovascular disease (severe)
**Case**	**Figure**	**Age at skin biopsy/ PBMC collection**	**Sex**	**Disease status**	**Source of hiPSC reprogramming**	**Braak stage**	**APOE genotype**	**GRS**
**ID1**	**Ext 5e-f**	76	F	Control	Fibroblasts	-	3/3	0.01831
**ID7**	**Ext 5e-f**	83	M	Control	PBMCs	-	3/3	0.0093

Supplementary Table 2. Human demographics data. M: Male; F: Female. HFrEF: Heart Failure with Reduced Ejection Fraction. AD: Alzheimer’s disease. PBMC: peripheral blood mononuclear cell. hiPSC: human induced pluripotent stem cell. ADNC: Alzheimer's disease neuropathologic change. GRS: Genetic risk score.
