# Supplementary material for: Lymphoid gene expression supports neuroprotective microglia function
Source: Nature. 2025 Nov 5;648(8092):157–65. doi: 10.1038/s41586-025-09662-z (PMC12675299; doi:10.1038/s41586-025-09662-z)
Supplement: Supplementary file 2 — Reporting Summary [file 41586_2025_9662_MOESM2_ESM.pdf]

nature portfolio

Corresponding author(s): Anne Schaefer, Pinar Ayata

Last updated by author(s): August 14, 2025

Reporting Summary

Nature Portfolio wishes to improve the reproducibility of the work that we publish. This form provides structure for consistency and transparency in reporting. For further information on Nature Portfolio policies, see our Editorial Policies and the Editorial Policy Checklist.

Statistics

For all statistical analyses, confirm that the following items are present in the figure legend, table legend, main text, or Methods section.

n/a	Confirmed	
☐	☒	The exact sample size (*n*) for each experimental group/condition, given as a discrete number and unit of measurement
☐	☒	A statement on whether measurements were taken from distinct samples or whether the same sample was measured repeatedly
☐	☒	The statistical test(s) used AND whether they are one- or two-sided<br>*Only common tests should be described solely by name; describe more complex techniques in the Methods section.*
☐	☒	A description of all covariates tested
☐	☒	A description of any assumptions or corrections, such as tests of normality and adjustment for multiple comparisons
☐	☒	A full description of the statistical parameters including central tendency (e.g. means) or other basic estimates (e.g. regression coefficient) AND variation (e.g. standard deviation) or associated estimates of uncertainty (e.g. confidence intervals)
☐	☒	For null hypothesis testing, the test statistic (e.g. *F*, *t*, *r*) with confidence intervals, effect sizes, degrees of freedom and *P* value noted<br>*Give P values as exact values whenever suitable.*
☒	☐	For Bayesian analysis, information on the choice of priors and Markov chain Monte Carlo settings
☐	☒	For hierarchical and complex designs, identification of the appropriate level for tests and full reporting of outcomes
☐	☒	Estimates of effect sizes (e.g. Cohen's *d*, Pearson's *r*), indicating how they were calculated

Our web collection on statistics for biologists contains articles on many of the points above.

Software and code

Policy information about availability of computer code

Data collection	RNA/DNA quality check: 2100 Expert Software (v8.02.07.SI532), TapeStation Software<br>qPCR: StepOne Software (v2.3)<br>RNA/DNA sequencing: NextSeq System Suite, Illumina bcl2fastq2 Conversion Software v2.17<br>Imaging : Zen black 2012 software (v8.1), Zen Blue software (v3.8), NDP viewing software, Aperio iQC Software, NIS-Elements<br>Flow cytometry: FACSDiva<br>Proteomics: Xcalibur (version 4.4.16.14)<br>LTP: pClamp software (v10.7),<br>Mouse behavior: EthoVision (v9), Fusion (v5.0)<br>BCA: SkanIt Software 5.0 for Microplate Readers RE, ver. 5.0.0.42
Data analysis	Flow cytometry: FlowJo (v10.10)<br>Single Cell RNA-Seq: 10X Cell Ranger (v2.1.0), RStudio (2023.12.1+402), Seurat (v5.0.2)<br>MERFISH analysis: MERSCOPE visualizer software (Vizgen), Vizgen analyzing pipeline, CellPose, RStudio R 4.2.2, Seurat 5.0.1, and custom-made scripts.<br>Imaging analysis: ImageJ (v2.1.0), Zen 2011 software, Zen2012 (v8.1), Fiji, CellProfiler version 4.2.1, CellProfiler Analyst version 3.0.4, QuPath (v0.5.1), Imaris (v10)<br>Proteomics: DIA-NN (version 1.8), Perseus (version 1.6.15.0) and R (version 4.1.2)<br>Bulk RNA/DNA sequencing analysis: Trim Galore! (v0.6.6), FastQC v0.11.9, FastQC (v0.11.8), featureCounts (v2.0.0), HISAT2 package (v2.2.0), Bowtie2 (v2.2.8), Picard (v2.2.4), MACS2 (v2.1.0), Subread (v2.0.1), deepTools (v3.2.1), DESeq2 package (v1.36.0), GSEA 4.2.3 software, Ingenuity Pathway Analysis software (01-22-01), EnrichR, heatmaply (v1.2.1), ChIPseeker (v1.30.3), GenomicRanges (v1.46.1)<br>Data representation: GraphPad Prism (v9.1.2), R 4.2.1

The code for MERFISH analysis is available at https://github.com/SchaferLabUMassChan/Ayata-et-al_2025.
The code to analyze Olah et al.'s human single-cell sequencing data is available at https://github.com/tulsi92/pu1_paper_supp_fig7.
The code to analyze Green et al.'s human single-cell sequencing data and AD Knowledge Portal can be found at https://github.com/maggieeiggam/pu1_paper/tree/main.

For manuscripts utilizing custom algorithms or software that are central to the research but not yet described in published literature, software must be made available to editors and reviewers. We strongly encourage code deposition in a community repository (e.g. GitHub). See the Nature Portfolio guidelines for submitting code & software for further information.

Data

Policy information about availability of data

All manuscripts must include a data availability statement. This statement should provide the following information, where applicable:

- Accession codes, unique identifiers, or web links for publicly available datasets
- A description of any restrictions on data availability
- For clinical datasets or third party data, please ensure that the statement adheres to our policy

Sequencing and MERFISH data can be downloaded from the National Center for Biotechnology Information Gene Expression Omnibus (GEO): MERFISH: GSE275026; TRAP sequencing: GSE274896; single-cell sequencing: GSE296768; single-nuclei sequencing: GSE296523; iMgl sequencing: GSE296769; and ATAC sequencing: GSE296641. Raw LC-MS/MS data used for proteomics have been deposited to the Proteomehange Consortium via the PRIDE partner repository, with the dataset identifier PXD063383. Upon request, the lead contacts can provide any additional information required to reanalyze the data reported in this paper.

Research involving human participants, their data, or biological material

Policy information about studies with human participants or human data. See also policy information about sex, gender (identity/presentation), and sexual orientation and race, ethnicity and racism.

Reporting on sex and gender

This study did not involve humans, only postmortem samples from individuals. Demographic information, including sex, has been reported in Table 2. For PU.1 immunostaining, we used de-identified post-mortem brain tissue from 79- to 91-year-old individuals diagnosed with AD (n=3, 1♀, 2♂). For iMgl study, we used fibroblasts from a 76-year-old healthy ♂ or PBMCs from an 83-year-old healthy ♀.

Reporting on race, ethnicity, or other socially relevant groupings

This study does not report on race, ethnicity, or other socially relevant groupings, to minimize the likelihood of unintentional identification of de-identified samples.

Population characteristics

The covariate-relevant population characteristics of the human research participants are described in Supplementary Table 2. For PU.1 immunostaining, we used de-identified post-mortem brain tissue from 79- to 91-year-old individuals diagnosed with AD (n=3, 1♀, 2♂). For iMgl study, we used fibroblasts from a 76-year-old healthy ♂ or PBMCs from an 83-year-old healthy ♀.

Recruitment

This study did not recruit participants, only de-identified postmortem samples from individuals.

Ethics oversight

Postmortem human brain tissue samples for immunohistochemistry were obtained from patients who had enrolled in and provided consent for a brain donation program through the Neuropathology Brain Bank & Research CoRe at Mount Sinai. These samples were collected by ethical guidelines and institutional review board (IRB) approval at the Icahn School of Medicine at Mount Sinai, ensuring the privacy and dignity of the donors while supporting ongoing neurological research. Tissue was obtained from donors who had provided written informed consent for research use either directly or via their next of kin. Three post-mortem brain samples (frontal cortex) from patients affected by Alzheimer's Neuropathological Changes (ADNC). Neuropathological assessments were performed at the respective centers using standardized criteria, including Consortium to Establish a Registry for Alzheimer’s Disease (CERAD) neuritic plaque assessment and Braak neurofibrillary tangle staging. This study did not recruit participants, only de-identified postmortem brain samples from individuals. Research with de-identified autopsy material does not meet the federal regulatory definition of human subject research as defined in 45 CFR part 46. However, HIPAA requirements still apply.

Human samples for induced pluripotent stem cell (hiPSC) lines were generated by the Icahn School of Medicine at Mount Sinai, UCI ADRC, WashU ADRC Induced Pluripotent Stem Cell Core from subject fibroblasts or peripheral blood mononuclear cells (PBMCs) with approved Institutional Review Boards (IRB) and Human Stem Cell Research Oversight (hSCRO) committee protocols at the Icahn School of Medicine at Mount Sinai, UCI ADRC, WashU ADRC Induced Pluripotent Stem Cell Core. The consent for reprogramming human somatic cells to hiPSC was obtained through the hSCRO protocol 19-04, 2013-9561, and 2017-1061. Informed consent was received by each of the participants who donated fibroblasts or PBMCs.

Note that full information on the approval of the study protocol must also be provided in the manuscript.

Field-specific reporting

Please select the one below that is the best fit for your research. If you are not sure, read the appropriate sections before making your selection.

☒ Life sciences ☐ Behavioural & social sciences ☐ Ecological, evolutionary & environmental sciences

For a reference copy of the document with all sections, see nature.com/documents/nr-reporting-summary-flat.pdf

Life sciences study design

All studies must disclose on these points even when the disclosure is negative.

Sample size	We do not include a justification of sample size for this study. We used the minimum number of animals needed to reliably detect the expected effect size with an alpha rate set at .05 in a standardly powered experiment and based on extensive laboratory experience and literatures in the field. There were additional practical constraints related to the availability of samples and appropriate controls in the vitro experiments, such as primary cell yield from animals and limitations in treatment or read-out methods, such as siRNA treatment or mass spectrometry. For human studies high-quality sample availability was the limiting factor. These considerations collectively informed our sample size, which we deemed sufficient to observe biologically meaningful trends within the context of the study's aims. Sample sizes are sufficient to detect significant changes based on previous studies employing similar methodologies and experimental designs in the field.
Data exclusions	Data were only formally excluded if identified as a statistically significant outlier by Grubbs' outlier test. If an outlier was identified by Grubbs's test, this outlier is removed from the dataset and the test is iterated until no outliers are detected.
Replication	All attempts of replications were successful. Each experiment was reproduced independently with similar results. Reproducibility has been either indicated in the Figure Legends, or is shown as a quantification.
Randomization	For all experiments involving treatment with CSF1R or PLC inhibitors, animals or tissue culture wells were randomly assigned to groups. In other experiments, the mice were allocated into groups based on either genotype or age. We did not compare human populations (control vs. AD), but rather only within individuals with AD.
Blinding	Experimenters were blinded during imaging and behavioral experiments. Gene expression and Western blot analyses were not performed blind because they rely on quantitative readouts lacking any subjectivity that could be influenced by knowledge of the experimental condition. Sample processing was done blinded or by batch analysis.

Behavioural & social sciences study design

All studies must disclose on these points even when the disclosure is negative.

Study description	This study is not on behavioural & social sciences.
Research sample	N/A
Sampling strategy	N/A
Data collection	N/A
Timing	N/A
Data exclusions	N/A
Non-participation	N/A
Randomization	N/A

Ecological, evolutionary & environmental sciences study design

All studies must disclose on these points even when the disclosure is negative.

Study description	This study is not on ecological, evolutionary & environmental sciences.
Research sample	N/A
Sampling strategy	N/A
Data collection	N/A
Timing and spatial scale	N/A
Data exclusions	N/A
Reproducibility	N/A
Randomization	N/A

Blinding	N/A

Did the study involve field work? ☐ Yes ☒ No

Reporting for specific materials, systems and methods

We require information from authors about some types of materials, experimental systems and methods used in many studies. Here, indicate whether each material, system or method listed is relevant to your study. If you are not sure if a list item applies to your research, read the appropriate section before selecting a response.

Materials & experimental systems

n/a	Involved in the study
☐	☒ Antibodies
☐	☒ Eukaryotic cell lines
☒	☐ Palaeontology and archaeology
☐	☒ Animals and other organisms
☒	☐ Clinical data
☒	☐ Dual use research of concern
☒	☐ Plants

Methods

n/a	Involved in the study
☒	☐ ChIP-seq
☐	☒ Flow cytometry
☒	☐ MRI-based neuroimaging

Antibodies

Antibodies used

Immunohistochemistry:

NP-tau (AT8, 1:500; Thermo Fisher Scientific, MN1020B); Aβ (biotinylated, anti-Aβ1–16, mouse monoclonal, 2µg/ml generated in-house, clone number: HJ3.4), PU.1 1:50 (clone 9G7, Cell Signaling 2258S), IBA1 1:500 (Thermo Fisher Scientific, PA5-27436), β-amyloid 1:4000 (clone 4G8, BioLegend, #800701), and secondary antibody Multimer HRP OminiMAP-Anti Mouse and Multimer HRP OminiMAP-Anti Rabbit (760-4310 & 760-4311, Roche Diagnostics, 1:1)

MERFISH:

β-amyloid (Purified azide-free anti-β-amyloid, clone: 6E10, #803004, Biolegend 1:100), secondary antibody (Vizgen, Anti-Mouse Aux 4 #20300101 1:100)

Immunofluorescence:

β-amyloid (6E10, 1:500, BioLegend, 803004); IBA1 (1:500, Wako 019-19741); CD11B (1:1000, MCA711GT, Biorad) PU.1 (1:200 Cell Signaling #2258); CD11b (1:500 Thermo #14-0112-85); IBA1 (1:750, Synaptic Systems, #234 009); Bassoon (1:500, Enzo Lifesciences, ADI-VAM-PS003-F); VGluT2 (1:2000, Synaptic Systems, 135404); C1Q (1:500, Abcam, ab182451); turboGFP (1:5000, Evrogen, AB513); CD28 (1:500, Cell Signaling #38774); Alexa Fluor 488-, 546-, and 568-labeled goat anti-mouse, goat anti-rat, goat anti-chicken, goat anti-rabbit, or donkey anti-goat IgGs (H+L); 1:500, Life Technologies); Tyramide SuperBoostTM Kit goat anti-rabbit IgG ThermoFisher #B40922, Tyramide Conjugate Invitrogen #B40956, 1:1, according to manufacurer's instructions)

Immunoblotting:

SYK (1:500, Cell Signaling #2712), Phospho-SYK Y352 (1:500, Cell Signaling #2717), PLCG2 (1:500, Cell Signaling #3852), Phospho-PLCG2 Y1217 (1:500, Cell Signaling #3871), PU.1 (1:500, Cell Signaling #2266), CD28 (1:500, Abcam ab243228), ACTB (1:2000, Abcam ab8227), H3 (1:5000, Abcam ab1791), TUBULIN (1:1000 ab6046), IBA1 (1:500, Wako, 016-20001), C1QA (1:500, Proteintech, 11602-1-AP). Secondary antibodies: horseradish-peroxidase-conjugated anti- mouse (Cat#31438, 1:10,000, Life Technologies), horseradish-peroxidase-conjugated anti-rabbit IgG secondary antibody (NA934V, 1:10,000, GE), horseradish-peroxidase-conjugated anti-sheep IgG secondary antibody (A16041, 1:10,000, Invitrogen), horseradish-peroxidase-conjugated anti-rat IgG secondary antibody (31470, 1:10,000, Invitrogen).

TRAP:

mouse monoclonal anti-GFP (clone 19F7 (cat# HtzGFP19F7) and clone 19C8 (cat# HtxGFP-19C8), Antibody & Bioresource Core Facility Memorial Sloan Kettering Cancer Center)

Flow Cytometry:

APC-CD11B (rat anti-mouse, eBioscience, 17-0112-82, 1:100); PerCP-Cy5.5-CD45 (rat anti-mouse, Invitrogen, 45-0451-82, 1:100).

Validation

IHC/Immunostaining, MERFISH antibodies: β-amyloid (clone 4G8, BioLegend, #800701), IBA1 (1:500, Wako 019-19741 and Thermo Fisher Scientific, PA5-27436), CD11B (14-0112-85), NP-tau (MN1020B), PU.1 (2258), Bassoon (ADI-VAM-PS003-F), VGluT2 (135404), C1Q (ab182451), turboGFP (AB513) are validated for immunostaining of microglia in mouse on the manufacturers website. 6E10 (803004) has been validated in multiple publications for use in mouse including Ennerfeldt et al 2022. PU.1 (2258) was additionally

validated in house with knock out mice. CD28 antibody was validated on CD28-KO mouse. All secondary antibodies are validated for immunostaining on the manufacturers website as well as by hundreds of publications.

Immunoblotting antibodies: SYK (2712), Phospho-SYK Y352 (2717), PLCG2 (3852), Phospho-PLCG2 Y1217 (3871), PU.1 (2266), CD28 (ab243228), Actin (ab8227), H3 (ab1791), IBA1 (016-20001), TUBULIN (ab6046), C1Qa (11602-1-AP), are verified for immunoblotting in mouse on the company website. PU.1 and PLCG2 were additionally validated in house with knock out mice. PU.1 and CD28 were additionally validated in house with knock down cell lines.

Mouse monoclonal anti-GFP (19F7 and 19C8, Antibody & Bioresource Core Facility Memorial Sloan Kettering Cancer Center) were verified for TRAP in Heiman et al. 2008 and Doyle et al. 2008.

APC-CD11B (eBioscience); PerCP-Cy5.5-CD45 (Invitrogen) are verified for FACS on the company website. Per the manufacturer's website, APC-CD11B (clone M1/70) antibody has been tested by flow cytometric analysis of mouse splenocytes, while the PerCP-Cy5.5-CD45 (clone 30-F11) antibody has been tested by flow cytometric analysis of mouse bone marrow cells and splenocytes.

Specifics on manufacturer validation can be accessed on the company website using the product catalogue numbers listed above.

Eukaryotic cell lines

Policy information about cell lines and Sex and Gender in Research

Cell line source(s)

The BV2 mouse microglial cell line was kindly provided by Marc Diamond (UT Southwestern Medical Center). The BV2 cell line has been maintained by the Goate Lab for the last 15 years and in the Schaefer Lab for the last 10 years, and stored long-term in liquid nitrogen. BV2 cells have been authenticated based on their characteristic microglia-like morphology, positive immunolabeling for microglia markers CD11b and Iba1 and PU.1 as well as gene and protein expression analysis using RNA seq and proteomic analysis. Mycoplasma contamination testing is done on a regular basis every 3 months and all cell lines tested negative for mycoplasma contamination.

Human iPSCs were generated by Icahn School of Medicine at Mount Sinai, UCI ADRC, WashU ADRC Induced Pluripotent Stem Cell Core from subject fibroblasts or PBMCs.

Authentication

The cell lines have been authenticated as indicated above based on their characteristic microglia-like morphology, positive immunolabeling for microglia markers CD11b and Iba1 and PU.1 as well as gene and protein expression analysis using RNA sequencing and proteomic analysis..

Mycoplasma contamination

Mycoplasma contamination testing is done on a regular basis every 3 months and all cell lines tested negative for mycoplasma contamination.

Commonly misidentified lines
(See ICLAC register)

No commonly misidentified cell lines were used.

Palaeontology and Archaeology

Specimen provenance

This study did not involve palaeontology and archaeology.

Specimen deposition

N/A

Dating methods

N/A

☐ Tick this box to confirm that the raw and calibrated dates are available in the paper or in Supplementary Information.

Ethics oversight

N/A

Note that full information on the approval of the study protocol must also be provided in the manuscript.

Animals and other research organisms

Policy information about studies involving animals; ARRIVE guidelines recommended for reporting animal research, and Sex and Gender in Research

Laboratory animals

Mice were housed in cages with two to five animals per cage, with a 12-hour light/dark cycle (lights on from 0700 to 1900 hours), a constant temperature (23ºC), and ad libitum access to food and water. The humidity was on average 38%, with a high of 58% and a low of 31% in a 24 hour period. All animal protocols were approved by IACUC at Icahn School of Medicine at Mount Sinai and performed according to NIH guidelines.

Mice of C57Bl/6 background were used. For lines that were generated in other strains, lines were backcrossed for >5 generations to C57/B6 mice. Male and female mice of all genotypes up to 20 months were used for all experiments performed in this study (except for the survival curve). Whenever male and female mice were used together, the ratio in control and treatment groups were equal. Whenever possible, littermate controls were used. For microglia isolation experiments, neonatal pups (post natal day 10) were used.

The following lines were used in this study: Spi1fl/+ (Jackson Laboratory, 006922), Cx3cr1creErt2/+ (Jackson Laboratory, 021160),

Cd28fl/fl (Jackson Laboratory, 024282), and Sykfl/fl (Jackson Laboratory, 017309).

Plcg2fl/+ mice were provided by Takeshi Inoue and Tomohiro Kurosaki (Riken Institute, Japan).

Eef1a1LSL.eGFPL10a/+ mouse was generously provided by A. Domingos (Instituto Gulbenkian de Ciência, PT) and Jeff Friedman (Rockefeller University).

Mx1::GFP mouse was generously provided by Dr. Adolfo García-Sastre (Icahn School of Medicine at Mount Sinai).

Spi1-overexpressing mice were generated in house as described in the manuscript methods.

Wild animals

This study did not involve wild animals.

Reporting on sex

TRAP sequencing: control (3♀, 3♂), 5xFAD (3♀, 3♂ on control diet or 2♀, 2♂ on PLX5622 CSF1Ri diet), PU.1-low (4♀, 2♂), PU.1-high mice (3♀, 1♂) , 5xFAD-PU.1-low (1♀, 3♂), 5xFAD-PU.1-high (1♀, 2♂)
10X single-cell sequencing: Control-3m (1♂), Control-6m (1♂), Control-8m (2♂), 5xFAD-3m (2♂), 5xFAD-6m (1♂), 5xFAD-8m (2♂), 5xFAD-CD28-KO-3m (2♂), 5xFAD-CD28-KO-6m (1♂)
10X single-nuclei sequencing: 5xFAD (1♀) on PLX5622 CSF1Ri diet (2♂), 5xFAD (1♀), 5xFAD-PU.1-low (1♂), 5xFAD-PU.1-high (1♂)
Amyloid plaque and tau immunohistochemistry: 5xFAD (7♀, 5♂) and 5xFAD; PU.1-low (4♀, 5♂)
Plaque and amyloid imaging: 5xFAD-3m (1♀, 5♂), 5xFAD-6m (2♀, 3♂), 5xFAD-CD28-KO-3m (2♀, 3♂), 5xFAD-CD28-KO-6m (4♀, 3♂)
PU.1 immunofluorescence in signaling knockouts: 5xFAD (3♀, 2♂), 5xFAD; SYK-KO (1♀, 2♂), 5xFAD; PLCG2-KO (2♀, 1♂), control (2♀, 2♂), SYK-KO (2♀, 1♂), PLCG2-KO (3♂)
Synapse imaging: control (4♀, 6♂), 5xFAD (7♀, 9♂), 5xFAD+CSF1Ri (3♀, 3♂), 5xFAD; PU.1-low (4♀, 2♂), and 5xFAD-PU.1-low;PLCγ2-low mice (1♀, 3♂)
LTP: (3♀, 2♂), 5xFAD (4♀, 2♂), and 5xFAD; PU.1-low mice (1♀, 4♂)
Open field: control (9♀, 6♂), 5xFAD (6♀, 6♂), and 5xFAD; PU.1-low (7♀, 6♂)
NOR: control (5♀, 5♂), 5xFAD (6♀, 5♂), and 5xFAD; PU.1-low mice (7♀, 8♂)
MERFISH: All ♂
For the remaining imaging and Western blot experiments sex-balanced mixed-sex cohorts were used.

Field-collected samples

This study did not involve samples collected from the field.

Ethics oversight

All protocols were approved by IACUC at Icahn School of Medicine at mount Sinai and were in accordance with NIH guidelines.

Note that full information on the approval of the study protocol must also be provided in the manuscript.

Clinical data

Policy information about clinical studies

All manuscripts should comply with the ICMJE guidelines for publication of clinical research and a completed CONSORT checklist must be included with all submissions.

Clinical trial registration

This study did not involve clinical research.

Study protocol

N/A

Data collection

N/A

Outcomes

N/A

Dual use research of concern

Policy information about dual use research of concern

Hazards

Could the accidental, deliberate or reckless misuse of agents or technologies generated in the work, or the application of information presented in the manuscript, pose a threat to:

No	Yes	
☒	☐	Public health
☒	☐	National security
☒	☐	Crops and/or livestock
☒	☐	Ecosystems
☒	☐	Any other significant area

Experiments of concern

Does the work involve any of these experiments of concern:

No	Yes	
☒	☐	Demonstrate how to render a vaccine ineffective
☒	☐	Confer resistance to therapeutically useful antibiotics or antiviral agents
☒	☐	Enhance the virulence of a pathogen or render a nonpathogen virulent
☒	☐	Increase transmissibility of a pathogen
☒	☐	Alter the host range of a pathogen
☒	☐	Enable evasion of diagnostic/detection modalities
☒	☐	Enable the weaponization of a biological agent or toxin
☒	☐	Any other potentially harmful combination of experiments and agents

Plants

Seed stocks	This study did not involve plants.
Novel plant genotypes	N/A
Authentication	N/A

ChIP-seq

Data deposition

☐ Confirm that both raw and final processed data have been deposited in a public database such as GEO.

☐ Confirm that you have deposited or provided access to graph files (e.g. BED files) for the called peaks.

Data access links<br>*May remain private before publication.*	This study did not involve ChIP-seq.
Files in database submission	N/A
Genome browser session<br>(e.g. UCSC)	N/A

Methodology

Replicates	N/A
Sequencing depth	N/A
Antibodies	N/A
Peak calling parameters	N/A
Data quality	N/A
Software	N/A

Flow Cytometry

Plots

Confirm that:

☒ The axis labels state the marker and fluorochrome used (e.g. CD4-FITC).

☒ The axis scales are clearly visible. Include numbers along axes only for bottom left plot of group (a 'group' is an analysis of identical markers).

☒ All plots are contour plots with outliers or pseudocolor plots.

☒ A numerical value for number of cells or percentage (with statistics) is provided.

Methodology

Sample preparation	For microglia phospho-protein analysis (Supplementary Fig 11b), PU.1-low, wild-type, and PU.1-high mice were euthanized by cervical dislocation, and brain regions were immediately removed. Frozen tissue was mechanically dissociated in glass homogenizers in HBSS supplemented with protease and RNase inhibitors. The homogenate was filtered through a 70-µm mesh filter. Myelin removal was performed using Percoll (pH 7.4) density gradient separation. The homogenate was supplemented with 90% Percoll (17-0891-02, Amersham, Amersham, UK) with PBS (pH 7.4). The resulting homogenate in 21% Percoll gradient was centrifuged at 500g for 15 min at 4°C. The pellet was washed and resuspended in HBSS. Microglia were gated as live cells, YFP+ in Cx3cr1CreErt2/+(Litt) mice (Supplementary Fig. 9b). Roughly 120,000 cells were used per sample.<br><br>For ATAC sequencing, wild-type, PU.1-low, and PU.1-high mice were anesthetized with an intraperitoneal injection of Ketamine/Xylazine, transcardially perfused with 12mL of cold HBSS. The brains were collected, and the cerebellum and olfactory bulb were removed. Tissue was mechanically dissociated in glass homogenizers in HBSS. Myelin removal was performed using Percoll (pH 7.4, GE-17-0891-02) density gradient separation in 30% Percoll centrifuged at 500 g for 15 min at 4°C. The pellet was washed and resuspended in MACS buffer (2 mM EDTA and 0.5% BSA in 1x PBS) with DAPI and sorted on the FACS Aria II (BD Biosciences). Microglia were gated as live cells, YFP+ (Supplementary Fig. 9b).<br>Spleens were harvested and homogenized on ice using a 70 µm strainer. Red blood cells were lysed with RBC lysis buffer (eBioscience, 00-4333-57) for 5 minutes at room temperature, followed by a PBS wash and centrifugation. Cells were resuspended in MACS buffer, incubated with Fc-blocker (Bio-rad, BUF041B, 1:100) on ice for 10 mins, stained with THY1.2 (eBioscience, 17-0902-82, 1:100) and DAPI, and sorted on the FACS AriaII (BD Biosciences). T-cells were gated as live cells expressing THY1+. Roughly 50,000 cells were used per sample.<br><br>For single-cell sequencing and CD28 analysis, Wild-type and 5xFAD mice were euthanized by CO_2 asphyxiation, and brain regions were immediately removed. Fresh tissue was mechanically dissociated in glass homogenizers in HBSS and then supplemented with fluorescently conjugated antibodies (APC-CD11B, eBioscience, 17-0112-82, 1:100; and PerCP-Cy5.5-CD45, Invitrogen, 45-0451-82, 1:100) and incubated for 30 minutes on ice. Cells were washed twice (300g centrifugation for 5 minutes at 4°C) and resuspended in FACS buffer (PBS, 2% BSA). Cells were sorted on the FACS AriaII (BD Biosciences). Microglia were gated as live cells, CD11B+, Cd45med (Supplementary Fig. 1c). Roughly 250,000 (Supplemenary Fig. 10a) and 700,000 (Supplementary Fig. 15b) cells were used per well for CD28 Western blotting.<br><br>For nuclei isolation: mice were euthanized with CO_2 and brain regions were quickly dissected and homogenized in 0.25 M sucrose, 150 mM KCl, 5 mM $MgCl_2$, 20 mM Tricine pH 7.8 supplemented with protease and RNase inhibitors with a glass Dounce homogenizer (1984-10002, Kimble Chase, Vineland, NJ). The buffers were supplemented with 10 µl/ml RNasin, Superasin, and EDTA-free protease inhibitor cocktail (11836170001, Roche). The homogenate was then spun through a 29% iodixanol cushion. The resulting nuclear pellet was resuspended in 0.25 M sucrose, 150 mM KCl, 5 mM $MgCl_2$, 20 mM Tricine pH 7.8, supplemented with 10 µM DyeCycle Ruby (V10304, Invitrogen), and 10% donkey serum (017-000-121, Jackson Immunoresearch, West Grove, PA). Microglial nuclei were sorted in a BD FACS Aria cell sorter by gating for the lowest DyeCycle Ruby, which indicates nuclei singlets and a high GFP signal.
Instrument	FACS AriaII (BD Biosciences)
Software	FACSDiva, FlowJo
Cell population abundance	Cell populations were all indicated in Supplementary Figures 1b, 2e, and 9b.
Gating strategy	Compensation was performed on single-stained samples of UltraComp eBeads (ThermoFisher), unstained beads, and unstained cells. Forward and side scatter was used to gate on a defined population of cells to exclude debris and also select single cells. Live cells were determined as DyeCycle Violet negative. Gates to define positive and negative cells were determined using unstained samples.

☒ Tick this box to confirm that a figure exemplifying the gating strategy is provided in the Supplementary Information.

Magnetic resonance imaging

Experimental design

Design type	This study did not involve MRI.

Design specifications | N/A

Behavioral performance measures | N/A

Acquisition

Imaging type(s) | N/A

Field strength | N/A

Sequence & imaging parameters | N/A

Area of acquisition | N/A

Diffusion MRI ☐ Used ☒ Not used

Preprocessing

Preprocessing software | N/A

Normalization | N/A

Normalization template | N/A

Noise and artifact removal | N/A

Volume censoring | N/A

Statistical modeling & inference

Model type and settings | N/A

Effect(s) tested | N/A

Specify type of analysis: ☐ Whole brain ☐ ROI-based ☐ Both

Statistic type for inference (See Eklund et al. 2016) | N/A

Correction | N/A

Models & analysis

n/a	Involved in the study
☒	☐ Functional and/or effective connectivity
☒	☐ Graph analysis
☒	☐ Multivariate modeling or predictive analysis
