# Supplementary material for: Lymphoid gene expression supports neuroprotective microglia function
Source: Nature. 2025 Nov 5;648(8092):157–65. doi: 10.1038/s41586-025-09662-z (PMC12675299; doi:10.1038/s41586-025-09662-z)
Supplement: Supplementary file 3 — Supplementary Table 1. Single-cell sequencing from wild-type and 5xFAD mice.Supplementary Table 2. Human demographics data. Supplementary Table 3. Single-nucleus sequencing from 5xFAD and 5xFAD+CSF1Ri microglia. Supplementary Table 4. MERFISH from 5xFAD–PU.1-low–wt–high microglia. Supplementary Table 5. TRAP sequencing from 5xFAD and 5xFAD+CSF1Ri microglia. Supplementary Table 6. TRAP sequencing from PU.1-low–wt–high microglia. Supplementary Table 7. ATAC sequencing from PU.1-low–wt–high microglia and T cells. Supplementary Table 8. Proteomics from PU.1-low–wt–high BV2 cells. Supplementary Table 9. RNA sequencing from PU.1-low iMgls. Supplementary Table 10. Single-nucleus sequencing from 5xFADPU.1-low–wt–high microglia. Supplementary Table 11. TRAP-seq from 5xFADPU.1-low–wt–high microglia. Supplementary Table 12. Single-cell sequencing from 5xFAD–CD28KO microglia. Supplementary Table 13. Single-nucleus sequencing from 5xFAD–TIM-3-KO microglia from Kimura et al.49. [file 41586_2025_9662_MOESM3_ESM.zip › 2024-08-16775C-s3/List of Supplementary Data.docx]

**List of Supplementary Data**

Supplementary Information

Supplementary Table 1 - Single-cell sequencing from WT, 5xFAD

Supplementary Table 3 - Single-nuclei sequencing from 5xFAD, 5xFAD+CSF1Ri microglia

Supplementary Table 4 - MERFISH from 5xFAD-PU.1-low-wt-high microglia

Supplementary Table 5 - TRAP sequencing from 5xFAD, 5xFAD+CSF1Ri microglia

Supplementary Table 6 - TRAP sequencing from PU.1-low-wt-high microglia

Supplementary Table 7 - ATAC sequencing from PU.1-low-wt-high microglia, T cells

Supplementary Table 8 - Proteomics from PU.1-low-wt-high BV2 cells

Supplementary Table 9 - RNA sequencing from PU.1-low iMgls

Supplementary Table 10 - Single-nuclei sequencing from 5xFADPU.1-low-wt-high microglia

Supplementary Table 11 - TRAP seq from 5xFADPU.1-low-wt-high microglia

Supplementary Table 12 - Single-cell sequencing from 5xFAD-CD28KO microglia

Supplementary Table 13 - Single-nuclei sequencing from 5xFAD-TIM-3-KO microglia from Kimura et al.
